# Tuning Photodynamic Properties of BODIPY Dyes, Porphyrins’ Little Sisters

**DOI:** 10.3390/molecules26144194

**Published:** 2021-07-10

**Authors:** Wojciech Krzemien, Monika Rohlickova, Miloslav Machacek, Veronika Novakova, Jaroslaw Piskorz, Petr Zimcik

**Affiliations:** 1 Department of Inorganic and Analytical Chemistry, Poznan University of Medical Sciences, Grunwaldzka 6, 60-780 Poznan, Poland; krzemestro@gmail.com; 2 Faculty of Pharmacy in Hradec Kralove, Charles University, Ak. Heyrovskeho 1203, 50003 Hradec Kralove, Czech Republic; steklam@faf.cuni.cz (M.R.); machamil@faf.cuni.cz (M.M.); novavero@faf.cuni.cz (V.N.)

**Keywords:** photodynamic therapy, singlet oxygen, BODIPY, fluorescence, heavy atom effect

## Abstract

The photodynamic properties of a series of non-halogenated, dibrominated and diiodinated BODIPYs with a phthalimido or amino end modification on the phenoxypentyl and phenoxyoctyl linker in the *meso* position were investigated. Halogen substitution substantially increased the singlet oxygen production based on the heavy atom effect. This increase was accompanied by a higher photodynamic activity against skin melanoma cancer cells SK-MEL-28, with the best compound reaching an EC_50_ = 0.052 ± 0.01 µM upon light activation. The dark toxicity (toxicity without light activation) of all studied dyes was not detected up to the solubility limit in cell culture medium (10 µM). All studied BODIPY derivatives were predominantly found in adiposomes (lipid droplets) with further lower signals colocalized in either endolysosomal vesicles or the endoplasmic reticulum. A detailed investigation of cell death indicated that the compounds act primarily through the induction of apoptosis. In conclusion, halogenation in the 2,6 position of BODIPY dyes is crucial for the efficient photodynamic activity of these photosensitizers.

## 1. Introduction

Nature is indisputably a source of many amazing compounds that have become templates for organic chemists. Among them, porphyrins, which may be found in heme, chlorophyll, or bacteriochlorophyll, have evolved into versatile chromophores with a wide range of applications such as, in solar cells [1,2], bioimaging [3], chemosensing [4], and photodynamic therapy (PDT) [5,6]. PDT is a minimally invasive modality for the treatment of cancer, age-related macular degeneration, therapy of acne, or for killing infectious microorganisms [7]. It involves three harmless components—a photosensitizer (PS), visible light, and molecular oxygen. PS is activated by light absorption to a singlet excited state and then undergoes intersystem crossing to the triplet state. From this state, it can transfer the obtained energy to a ground state triplet oxygen, thus forming a highly reactive singlet oxygen [8], which is responsible for cell death. Interestingly, the first clinically approved PS was just a mixture of porphyrin oligomers sold under the trade name Photofrin^®^ [9]. Since then, several other porphyrin analogues have been clinically approved by the Food and Drug Administration (e.g., verteporfin in 2000) or by the European Medicine Agency (e.g., temoporfin in 2001, redaporfin in 2015, padeliporfin in 2017). Suitable photodynamic properties of porphyrins arise from their structure, composed of four pyrrole rings connected by methine bridges. Consequently, the free base porphyrin possesses 22-π electrons, of which 18-π electrons obey Hückel’s rule. Such a highly aromatic structure is responsible for the two main absorption bands in the visible region—an intense Sorret band located around 400 nm and a weak Q band close to the biological window of tissues (typically 500–650 nm, extinction coefficients < 10^4^ M^−1^ cm^−1^). The weak absorption at the Q band, which is typically used for excitation of PSs in PDT, makes the use of a high dosage of light or excessive concentrations of PSs a necessity, to induce the desired curative effect of PDT. That is why many synthetic analogues are being intensively investigated worldwide. Aside from well-known phthalocyanines [10], smaller porphyrin analogues, i.e., boron-dipyrromethene derivatives (BODIPY, 4,4-difluoro-4-bora-3a,4a-diaza-*s*-indacene according to IUPAC), have attracted the attention of scientists in the last decades [11].

BODIPY dyes consist of only two pyrrole units linked via a methine bridge resembling a half-cut porphyrin and are, therefore, known also as “Porphyrins’ Little Sister” [12,13,14]. Complexation with a BF_2_ unit provides a rigid planar tricyclic system, prevents *cis*/*trans* isomerization, and consequently allows π-electron delocalization along the carbon-nitrogen backbone [15]. Due to their intense fluorescence quantum yields, sharp excitation and emission spectra, and high photo- and chemostability, BODIPYs have been investigated mostly for chemical sensing [16,17] or diagnostic imaging [18,19]. Disclosed structure-activity relationships have revealed the possibility of increasing singlet oxygen production at the expense of fluorescence emission and also the possibility of shifting the main absorption band close to the biological window of tissues. Thanks to that, the BODIPYs became suitable PSs in PDT [20,21]. Although the activation of PSs in the red or near-infrared region is typically declared to be suitable for deeper penetration of light into biological tissues (light is less scattered, so there is lower interference with endogenous chromophores), the absorption in the region around 500 nm can also be advantageous. For example, excitation of PSs by green light was shown to prevent deep tissue damage to surrounding healthy cells in some superficial skin tumors [22].

**Scheme 1:**
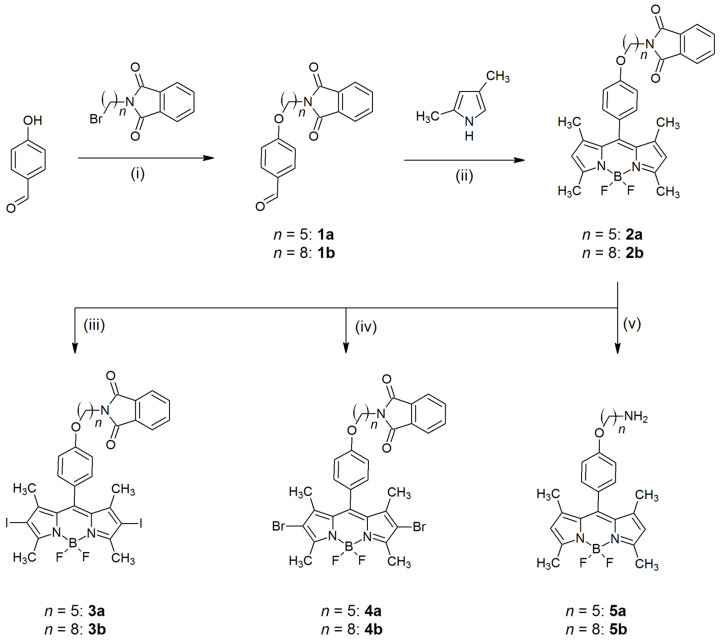
Synthesis of BODIPYs **2–5**. Reagents and conditions: (i) K_2_CO_3_, DMF, 70 °C; (ii) 1. CF_3_COOH, CH_2_Cl_2_, 1 h; 2. DDQ, CH_2_Cl_2_, 1 h; 3. DIPEA, BF_3_•Et_2_O, r.t., 6 h; (iii) I_2_, HIO_3_, C_2_H_5_OH, 70 °C, 30 min or NIS, CH_2_Cl_2_, r.t., 3 h (**3b**), (iv) NBS; CH_2_Cl_2_, DMF, r.t., 7 h (**4a**) or NBS; CH_2_Cl_2_, r.t., 17 h (**4b**), (v) NH_2_NH_2_•H_2_O, C_2_H_5_OH, reflux, 6 h.

This project aims to contribute to the studies on structure-activity relationships of BODIPY-based PSs from the point of view of spectral, photophysical, and in vitro anticancer activity against skin cancer cells (SK-MEL-28). The target compounds designed for this study are shown in Scheme 1. Different halogens (-I or -Br) were introduced in positions 2 and 6 of the BODIPY core to study the possibility of increasing singlet oxygen production based on the heavy atom effect, and compare them with corresponding control non-halogenated BODIPYs. A phthalimide or primary amino group was attached through a phenyloxypentyl or phenyloxyoctyl bridge into the *meso*-position of the BODIPY framework. Phthalimide has been reported to have a wide range of biological activities, including anti-inflammatory, analgesic, and cytotoxic effects [23,24,25], which may further support treatment by PDT. The primary amine may, on the other hand, increase the hydrophilicity of the molecule, which is generally beneficial in biomedical applications.

## 2. Results

### 2.1. Synthesis and Characterization

Two series of the BODIPY dyes which varied in the length of the alkyl linker between the peripheral phthalimido or amino group, and the phenoxyl substituent at the *meso* position of the BODIPY core, were obtained (Scheme 1). Firstly, new benzaldehyde derivatives 4-[5-(1,3-dihydro-1,3-dioxo-2*H*-isoindol-2-yl)pentyloxy]benzaldehyde (**1a**) and 4-[8-(1,3-dihydro-1,3-dioxo-2*H*-isoindol-2-yl)octyloxy]benzaldehyde (**1b**) were synthesized by the alkylation reactions of 4-hydroxybenzaldehyde with *N*-(5-bromopentyl)phthalimide and *N*-(8-bromooctyl)phthalimide, for **1a** and **1b**, respectively. The reaction conditions, using potassium carbonate in DMF at 70 °C, followed the procedure for the known phthalimidylpropyl analogue given by Dick et al. [26]. The next step was the acid-catalyzed condensation reactions of **1a** and **1b** with 2,4-dimethylpyrrole and complexation with boron trifluoride, following a previously elaborated procedure [27], and led to the novel BODIPYs bearing phthalimidylpentyloxyphenyl (**2a**) and phthalimidyloctyloxyphenyl (**2b**) substituents at the *meso* position. Subsequent reactions with iodine and iodic acid in ethanol gave disubstituted analogues possessing iodine atoms at positions 2 and 6 of the BODIPY core, with the yield of 69% and 61%, for **3a** and **3b**, respectively. In addition, an alternative iodination procedure with *N*-iodosuccinimide (NIS) in dichloromethane was used in the case of **3b**, and led to the enhancement of the reaction yield to 79%. The bromine atoms were also introduced at the 2 and 6 positions of the BODIPY core by the reactions of **2a** and **2b** with *N*-bromosuccinimide (NBS) in dichloromethane, providing brominated derivatives **4a** and **4b**. The BODIPYs **2a**, **2b** were also treated with hydrazine monohydrate to give primary amines **5a** and **5b**, resulting from the decomposition of the phthalimidyl substituent (Scheme 1). Any attempt to prepare primary amines from the brominated or iodinated compounds failed and only complex mixtures of compounds were obtained.

Obtained compounds were isolated and purified using crystallization and chromatographic methods, and then characterized by UV-Vis spectrophotometry, high-resolution mass spectrometry (HRMS), and various NMR techniques, including ^1^H NMR, ^13^C NMR, ^1^H-^1^H COSY, ^1^H-^13^C HSQC, and ^1^H–^13^C HMBC. The ^1^H NMR spectrum of **2a** in CDCl_3_ revealed two doublets of doublets at 7.85 and 7.72 ppm from the protons of the phthalimidyl group, two doublets at 7.14 and 6.97 ppm from four phenyl protons, and a singlet at 5.97 ppm from protons at positions 2 and 6 of the BODIPY core. In the higher field region, corresponding with aliphatic protons, five multiplet signals, which can be assigned to the protons of the pentyl chain, and two intensive singlets attributed to the protons of four methyl groups in the 1,3,5 and 7 positions of the BODIPY core, were observed. The ^1^H and ^13^C NMR spectra signals were assigned to the particular hydrogen and carbon atoms, based on the 2D NMR techniques as COSY, HSQC, and HMBC (see Supplementary Material). In the ^1^H NMR spectrum of derivatives **3a** and **4a**, only one significant change, such as the disappearance of the signal at 5.97 ppm from the protons at positions 2 and 6 of the BODIPY ring, was observed. It confirmed that hydrogens at these positions in **2a** were substituted by iodine or bromine atoms in the structures of **3a** and **4a**. Furthermore, the lack of any signals in the region of 7.7–7.9 ppm in the spectrum of **5a** confirmed the decomposition of the phthalimidyl group upon the reaction with hydrazine monohydrate. In the ^1^H NMR spectra of the BODIPYs **2b**–**5b** bearing octyl chain, similar signals to the described above pentyl series (**2a**–**5a**) were observed. The main difference was the presence of an additional signal from six protons resonating at about 1.4 ppm, which confirmed the extension of the alkyl chain by three methylene groups (see Supplementary Material).

### 2.2. Spectral Features and Photophysical Properties

**Figure 1:**
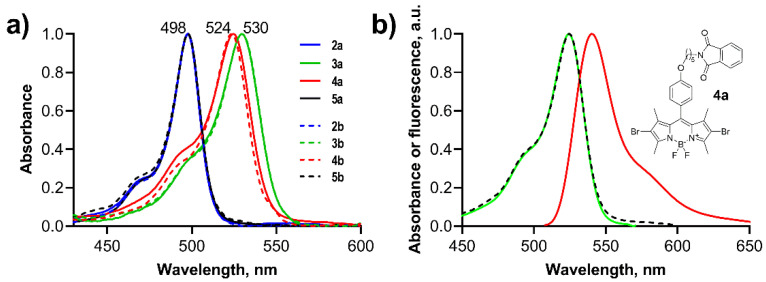
(**a**) Normalized absorption spectra of target compounds in MeOH (c = 1 μM); (**b**) Normalized absorption (black, dashed), emission (red), and excitation (green) spectra of **4a** in MeOH (c = 1 μM).

Spectral and photophysical properties of all target BODIPY dyes **2a,b**–**5a,b** were investigated in MeOH, and the obtained data are summarized in Table 1 and depicted in Figure 1. Compounds showed strong absorption in the area of 450–550 nm. As mentioned above, such an absorption profile of photosensitizers has been described to be advantageous for curing superficial cancer, because green light prevents deep tissue damage, thus, reducing the risk of perforation [22]. The main absorption bands of **2a,b**–**5a,b** in MeOH were sharp and narrow, demonstrating the resistance towards aggregation. The position of the absorption maximum was sensitive to the substituents in the 2,6-positions. The attachment of bromine or iodine caused the significant bathochromic shift of absorption maxima of 25 or 30 nm, respectively, due to contribution to the π-system of the BODIPY core, in accordance with the literature reports [28]. The spectra were also collected in a serum-containing cell culture medium (Figure S12) where a very small red shift (about 5–7 nm) was observed along with substantial broadening of the main absorption band of more lipophilic phthalimide-containing compounds **2a,b**–**4a,b**. To assess the photostability of the studied compounds, photobleaching experiments in the serum-containing cell culture medium were also performed. A small decrease in the absorption due to photodecomposition during irradiation was observed (Figure S13). The maximum decrease was to approximately 87% of the original value after 1 h of irradiation for compound **2a**. A closer analysis did not reveal any trends and relationships between the decomposition rate and the structure. However, since a decrease after 15 min of irradiation (used in the in vitro experiments) did not exceed 5% in any case, the compounds may be considered sufficiently stable and suitable for use in photodynamic treatments under investigational conditions.

Upon excitation, all the studied derivatives dissipated energy by fluorescence emission and singlet oxygen generation. Fluorescence emission spectra mirrored the absorption ones with only small Stokes shifts of maximally 17 nm, indicating negligible changes in the structure of compounds in excited states. Fluorescence excitation spectra resembled the shape of the corresponding absorption spectra, thus proving that the parent compound is responsible for the emission, and confirming the monomeric character of the compounds in solution. Fluorescence quantum yields (*Φ*_F_) and quantum yields of singlet oxygen production (*Φ*_Δ_) were determined in MeOH (Table 1). The ratio between *Φ*_Δ_ and *Φ*_F_ for each compound of the series followed the heavy atom rule well; thus, strong enhancement of a spin-forbidden process induced by the presence of an atom of a high atomic number was observed for halogenated compounds. Iodinated **3a**, **3b** had the highest *Φ*_Δ_ in the series (about 0.76), followed by the brominated derivatives **4a**, **4b** with a *Φ*_Δ_ value of approximately 0.55, and unsubstituted derivatives (**2a**, **2b**, **5a**, **5b**) with a *Φ*_Δ_ value lower than 0.07. This is in agreement with the literature data for structurally related BODIPY dyes [29,30]. Two different methods were employed for the determination of *Φ*_F_, i.e., an absolute method by an integrating sphere and a comparative method using rhodamin-G6 as a reference. The obtained values corresponded to each other, which confirmed the accuracy of the measurements. *Φ*_F_ values increased according to the 2,6-substitution in a series where -I < -Br < -H; reaching values of about 0.50 for the most fluorescent non-halogenated derivatives. Fluorescence lifetimes (*τ***_F_**) of **2a, b**–**5a, b** were in the range 0.17–3.27 ns and resembled the trend of *Φ*_F_. There were no significant differences in the photophysical data between compounds bearing a pentyl (“a” series) or octyl (“b” series) linker as well as between non-halogenated BODIPYs bearing a phthalimide (**2a, b**) or amino group (**5a, b**) as the end modification of the aliphatic linker.

From the above-mentioned facts, iodine-substituted derivatives **3a** and **3b** seem to be the most potent photosensitizers for photodynamic therapy with the most efficient intersystem crossing capacity. On the other hand, brominated species **4a** and **4b** may benefit from reasonably high singlet oxygen production with preserved fluorescence emission enabling easy localization of the drug in tissues.

### 2.3. In Vitro Assessment of Biological Activity

**Figure 2:**
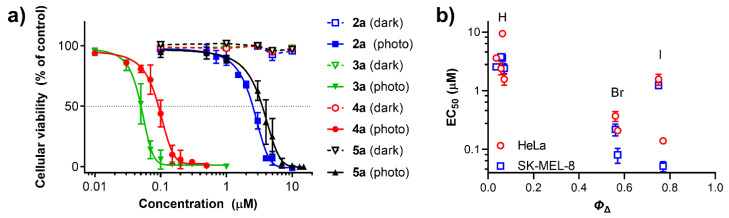
(**a**) Photodynamic activity (full lines, full symbols) and dark toxicity (dashed lines, empty symbols) of **2a**–**5a** on SK-MEL-28 cell line. At least four independent experiments each in triplicate were performed. Irradiation conditions: λ > 455 nm, 15.3 mW cm^−2^, 15 min, 13.7 J cm^−2^; (**b**) dependence of EC_50_ values of all investigated compounds (**2a**,**b**–**5a**,**b**) on their *Φ*_Δ_ values. Red circle (HeLa), blue square (SK-MEL-28).

The in vitro assessment of photodynamic activity after activation by light (λ > 455 nm, total light dose 13.7 J cm^−2^) was performed on a human cervical carcinoma (HeLa) cells and in more detail on melanoma cell lines (SK-MEL-28). The selection of the cell lines was driven primarily by spectral characteristics of the BODIPYs and their absorption around 500 nm. This leads to a rather shallow penetration of light into tissues, and that is why they are suitable primarily for superficial skin cancers (SK-MEL-28). The results were then also confirmed on the second cancer line—human cervical carcinoma (HeLa). The photodynamic activity was expressed as the half-maximal effective concentration (EC_50_, Figure 2a, Figures S14 and S15, Table 2). Results correlated well with the photophysical data determined in MeOH. Compounds with halogens in positions 2 and 6, in which the Φ_Δ_ value was much higher than for corresponding non-halogenated analogues, generally had much better photodynamic activities (Figure 2b). This highlighted the importance of the heavy atom effect in the design of novel PSs from the group of BODIPY dyes. Similar results were found for both series “a” and “b” irrespective of the length of the linker, except for **3b**, in which the photodynamic activity on both cell lines was lower than would correspond to structural assumptions. At this moment, we have no clear explanation of the atypical behavior of this compound. There was also no significant difference between compounds bearing a different end-modification (phthalimide (**2a, b**) or amino (**5a, b**) group), and both types of compounds were characterized by similar EC_50_ values. Despite the generally nonselective character of PDT, approximately 1.5–2.5× higher activity was observed against SK-MEL-28 cells than against HeLa cells with the exception of **2b** and **5b**. This difference is, however, small and most likely connected with the rather different growth rate of the cells. The inherent toxicity of the studied BODIPY dyes without light activation (i.e., dark toxicity) was determined on SK-MEL-28 cells and expressed as the half-maximal toxic concentration (TC_50_, Figure 2a, Figure S15, Table 2). The dark toxicity of all compounds was higher than 10 μM, which was the limit of solubility in the cell culture medium. Phototherapeutic indices (i.e., ratios between TC_50_ and EC_50_ values) ranged from >3 up to >192 for the most effective derivatives, thus giving a sufficient window to induce a curative effect with limited toxicity to non-irradiated cells.

Direct comparison with the literature data of various BODIPY dyes is problematic due to the different experimental conditions used. The following comparison must therefore, only be taken as approximation. Brominated BODIPYs were studied in vitro by Atilgan et al., who reported EC_50_ values on K562 human erythroleukemia cells of less than 0.2 μM for the best derivative of the series (red LED irradiation at 625 nm, fluence rate 2.5 mW cm^−2^ for 4 h) [33]. A biological evaluation of iodinated and non-iodinated BODIPY dyes carried out on human androgen-sensitive prostate adenocarcinoma cell line (photodiode of a specific wavelength, total light dose 2 J cm^−2^) revealed EC_50_ values comparable to the values of this study, i.e., dozens of nM and units of μM for iodinated and non-halogenated dyes, respectively [34]. Another in vitro PDT study of an iodinated BODIPY published by Caruso et al. on the human ovarian carcinoma cell line SKOV3, showed EC_50_ values in the range of 0.6–8 nM (irradiation by a green LED, total light dose 25.2 J cm^−2^) [35]. Further, unsymmetrical distyryl iodinated BODIPY dyes investigated against HT29 human colorectal carcinoma cells showed EC_50_ values in the range 0.015–1.0 μM (*λ* > 610 nm, 48 J cm^−2^) [36].

The obtained data for studied BODIPYs can also be compared to clinically approved PSs [37]. In comparison with these PSs, the studied BODIPY dyes appeared to be of medium or high PDT activity (see Table 2). The best derivatives of the series (**3a**, **4a**, and **4b**) had 3× higher EC_50_ values than temoporfin, marketed in the European Union under the tradename Foscan^®^. On the other hand, they have a 10× improved PDT activity than the sulfonated hydroxyaluminium Pc (S_3_AlOHPc), which has been approved in Russia under the tradename Photosens^®^. Looking to structurally related porphyrins, which may be considered as two BODIPY molecules together, the well-known protoporphyrin IX (PpIX) had an EC_50_ value of 0.80 ± 0.13 μM, which lies in the middle of the values determined for the studied BODIPY dyes.

**Figure 3:**
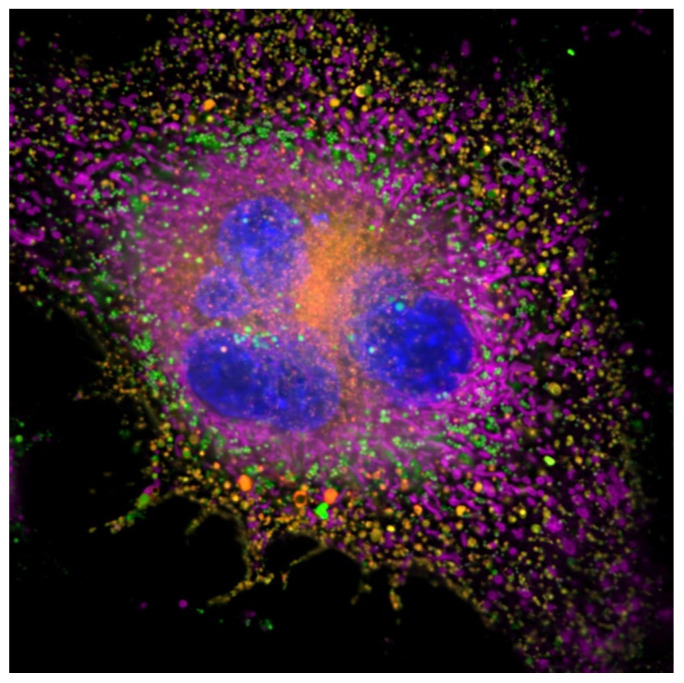
Subcellular localization of **4b**. Live-cell imaging of SK-MEL-28 cells after 12 h incubation with 1 μM **4b**. Intravital staining was used to label mitochondria (pink), nuclei (blue) and endolysosomal compartments (red). Compound **4b** displays its intrinsic fluorescence (green) and is localized to lysosomes (orange spots—colocalization with red probe for lysosomes) and adiposomes (lipid droplets); green—no colocalization with any applied organelle-specific probes). For further data from subcellular localization, see Figures S16–S19.

Determination of subcellular localization of PS is important as the primary target of oxidative damage upon irradiation is crucial for triggering cell death. Interestingly, accumulation inside cells differed slightly within the series as confirmed on SK-MEL-28 cells by fluorescence microscopy (Figure 3 and Figures S16–S19). All compounds showed a strong fluorescence signal colocalized with lipid droplets (adiposomes) and a weak fluorescence signal colocalized either with lysosomes (**3ab**, **4ab**, **5ab**) or the endoplasmatic reticulum (**2ab**). Similar localization in lipid droplets has also been reported for other lipophilic BODIPYs [38,39].

**Figure 4:**
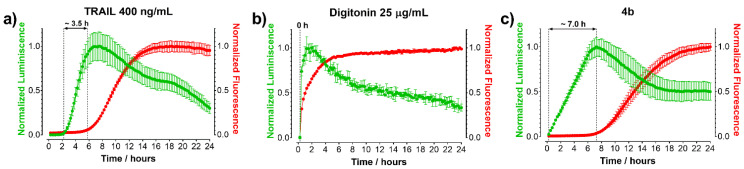
Cell death determination. Annexin V (green) and DNA-binding probe (red) binding profiles for TRAIL ((**a**) apoptosis control), digitonin ((**b**) necrosis control), and **4b** at EC_85_ (**c**).

Precise determination of cell death requires the utilization of several different methods. For the purpose of this study, the predominant and presumable type of cell death was revealed by the kinetic measurement of phosphatidylserine exposure to the outer leaflet of the cytoplasmic membrane and the loss of membrane integrity. Gradual exposure of phosphatidylserine during apoptosis can be measured by fluorophore-labeled Annexin V. This technique is commonly used in flow cytometry and fluorescence microscopy as an end-point analysis. In our study, real-time measurement of Annexin binding using Annexin fused with complementary NanoBiT^®^ Luciferase subunits (LgBiT and SmBiT) was used. While in solution, both subunits have a low affinity for each other producing a very low luminescence signal (that has to be subtracted using cell-free samples). Upon binding to phosphatidylserine on the cellular membrane both subunits appear in a close vicinity which leads to the complementation of functional luciferase and produces strong luminescence. A necrotic type of cell death can be characterized by permeabilization of both the cytoplasmic and nuclear membrane (apoptotic cells have an intact cytoplasmic membrane) that allows binding of certain cell-impermeant probes with an affinity for DNA, leading to an increase in fluorescence. Surveying time-dependent increases in both signals allows apoptotic and non-apoptotic (necrotic) cell death to be distinguished, as apoptosis is characterized by a substantial delay between increases in the luminescence and fluorescence (secondary necrosis) signals. In primary necrosis, an increase in both signals occurs simultaneously or in short succession. To confirm this hypothesis in our experimental setup, positive controls were included–namely TRAIL (400 ng/mL; inducer of extrinsic-pathway apoptosis), paclitaxel (1 μM; inducer of intrinsic-pathway apoptosis) and digitonin (25 μg/mL; membrane disrupting necrosis inducer). Both apoptosis inducers showed a substantial delay in the fluorescence signal surge—approximately 3.5 h and 11.5 h for TRAIL and paclitaxel, respectively. As expected, a simultaneous and immediate rise in luminescence and fluorescence was observed by digitonin (Figure 4 and Figure S21). To evaluate which type of cell death is triggered by the action of the studied BODIPYs, SK-MEL-28 cells were incubated for 12 h with concentrations corresponding to the respective EC_85_ values. The irradiation protocol was identical to the one used for the evaluation of phototoxicity. Activation of all studied photosensitizers by light leads to apoptotic cell death as marked by an ~7 h lag between luminescence and fluorescence accruement (Figure 4 and Figure S20).

## 3. Materials and Methods

### 3.1. Synthesis

#### 3.1.1. General

The reactions were carried out in glassware dried under an argon atmosphere. The reported reaction temperatures refer to the external bath temperatures. The solvents were rotary evaporated under vacuum at or below 60 °C. Flash column chromatography was carried out on Fluorochem silica gel with a particle size of 20–45 µm (Fluorochem, Hadfield, UK) and Merck 60 with particles 40–63 µm (Merck, Darmstadt, Germany). For the separations of compounds containing the primary amine groups, aluminium oxide gel 50–200 µm (Acros Organics, Geel, Belgium) and 40–160 µm (Sigma-Aldrich (Merck, Darmstadt, Germany)) were used. Thin-layer chromatography (TLC) was performed on SiliCycle 60 F254 or Merck Kieselgel 60 F254 plates and Merck neutral aluminium oxide 60 plates and visualized under UV light (λ_max_ 254 and 365 nm) (Merck, Darmstadt, Germany). The nuclear magnetic resonance (NMR) analysis was performed at room temperature using Bruker Avance and JEOL ECA spectrometers at 400, 500, and 600 MHz for the ^1^H spectra, and at 101, 126, and 151 MHz for the ^13^C spectra (Bruker, Billerica, MA, USA; JEOL, Tokyo, Japan). The following abbreviations for multiplicities were used: s—singlet, d—doublet, dd—double doublet, t—triplet, m—multiplet. The additional two-dimensional spectra, including ^1^H-^1^H COSY, ^1^H-^13^C HSQC, and ^1^H-^13^C HMBC, were used to assign the chemical shift values to the individual carbon and hydrogen atoms. The HRMS spectra were carried out by a high-resolution QTOF mass spectrometer Impact HD (Bruker, Billerica, USA). The UV-vis spectra were recorded using a Shimadzu UV-2600 spectrophotometer (Shimadzu Europe, Prague, Czech Republic). Infrared spectra were measured on a Nicolet 6700 (ATR mode) (Thermo Fisher Scientific, Waltham, MA, USA).

##### 4-[5-(1,3-Dihydro-1,3-dioxo-2*H*-isoindol-2-yl)pentyloxy]benzaldehyde (**1a**)

4-Hydroxybenzaldehyde (2.44 g, 20 mmol), K_2_CO_3_ (3.59 g, 26 mmol), and *N*-(5 bromopentyl)phthalimide (5.92 g, 20 mmol) were dissolved in 10 mL of DMF and stirred at 70 °C for 24 h. A reaction mixture was then poured out onto a mixture of ice-water and the resulting pale yellow precipitate was filtered off, dried and then dissolved in boiled ethyl acetate. The solution was filtered, cooled, and crystallized using *n*-pentane to give a white crystalline solid of **1a** (5.38 g, 80%), *R*_f_ = 0.09 (dichloromethane). ^1^H NMR (400 MHz, CDCl_3_) δ 9.86 (s, 1H, CHO), 7.83 (dd, *J* = 5, 3 Hz, 2H, H-phthalimidyl), 7.80 (d, *J* = 9 Hz, 2H, H-phenyl), 7.71 (dd, *J* = 5, 3 Hz, 2H, H-phthalimidyl), 6.96 (d, *J* = 9 Hz, 2H, H-phenyl), 4.03 (t, *J* = 6 Hz, 2H, -O-CH_2_-), 3.72 (t, *J* = 7 Hz, 2H, N-CH_2_), 1.91–1.82 (m, 2H), 1.81–1.72 (m, 2H), 1.59–1.47 (m, 2H). ^13^C NMR (101 MHz, CDCl_3_) δ 190.8, 168.4, 164.0, 133.9, 132.1, 131.9, 129.8, 123.2, 114.7, 67.9, 37.7, 28.5, 28.2, 23.2. HRMS (ES): *m*/*z* (Δ ppm)—with the intensity as a percentage of the maximal value. Calcd for C_20_H_19_NO_4_ 337.1314. Found [M]^+•^ 337.1311—<1% (0.9 ppm), [M + H]^+^ 338.1397—16% (1.5 ppm), [M + Na]^+^ 360.1222—100% (2.8 ppm).

##### 4-[8-(1,3-Dihydro-1,3-dioxo-2*H*-isoindol-2-yl)octyloxy]benzaldehyde (**1b**)

4-Hydroxybenzaldehyde (1.71 g, 14.0 mmol), K_2_CO_3_ (2.51 g, 18.2 mmol), and *N*-(8-bromoctyl)phthalimide (4.73 g, 14.0 mmol) were dissolved in DMF (10 mL) and stirred at 70 °C for 4 h. A reaction mixture was then poured out onto a mixture of ice-water, and the resulting creamy-white precipitate was filtered off, dried, and dissolved in boiled ethyl acetate. Next, the obtained solution was filtered, cooled, and crystallized using *n*-hexane to give a white crystalline solid of **1b** (3.42 g, 64%), *R*_f_ = 0.13 (dichloromethane). ^1^H NMR (500 MHz, CDCl_3_) δ 9.87 (s, 1H, -CHO), 7.83 (dd, *J* = 3, 5 Hz 2H, H-phthalimidyl), 7.81 (d, *J* = 9 Hz, 2H, H-phenyl), 7.70 (dd, *J* = 3, 5 Hz, 2H, H-phthalimidyl), 6.98 (d, *J* = 9 Hz, 2H, H-phenyl), 4.02 (t, *J* = 7 Hz, 2H, -O-CH_2_-), 3.68 (t, *J* = 7 Hz, 2H, N-CH_2_), 1.84–1.75 (m, 2H), 1.72–1.65 (m, 2H), 1.49–1.41 (m, 2H), 1.36 (s, 6H). ^13^C NMR (126 MHz, CDCl_3_) δ 190.8, 168.5, 164.3, 133.9, 132.2, 132.0, 130.0, 129.8, 123.2, 114.8, 68.3, 38.0, 29.1, 29.0, 28.5, 26.7, 25.8. HRMS (ES): *m*/*z* (Δ ppm)—with the intensity as a percentage of the maximal value. Calcd for C_23_H_25_NO_4_ 380.1862. Found [M + H]^+^ 380.1870—22% (2.1 ppm), [M + Na]^+^ 402.1688—100% (1.7 ppm), [M + K]^+^ 418.1425—3% (1.0 ppm).

##### 1,3,5,7-Tetramethyl-8-{4-[5-(phthalimidyl)pentyloxy]phenyl}-4,4-difluoro-4-bora-3a,4a-diaza-s-indacene (**2a)**

2,4-Dimethylpyrrole (0.31 mL, 3 mmol) and compound **1a** (0.51 g, 1.5 mmol) were dissolved in 60 mL of dichloromethane, and 2 drops of trifluoroacetic acid were added. After 1 h, DDQ (0.68 g, 3.0 mmol) dissolved in 30 mL of dichloromethane was added. After another hour, DIPEA (3.92 mL, 22.5 mmol) was added, and BF_3_•Et_2_O (3.7 mL; 30 mmol) was added dropwise. The reaction was carried out for 6 h at room temperature. Next, 100 mL of water was added, and the resulting two-phase mixture was filtered through Celite followed by extraction with water (2 × 100 mL). The collected organic layer was dried with anhydrous MgSO_4_ and evaporated. The solid residue was chromatographed using silica gel as a solid phase and dichloromethane as the eluent. The collected fractions were evaporated, and the residue was dissolved in dichloromethane and crystallized using *n*-pentane to give an orange solid of **2a** (0.45 g, 55%), *R*_f_ = 0.28 (dichloromethane). ^1^H NMR (400 MHz, CDCl_3_) δ 7.85 (dd, *J* = 5, 3 Hz, 2H, H-phthalimidyl), 7.72 (dd, *J* = 5, 3 Hz, 2H, H- phthalimidyl), 7.14 (d, *J* = 9 Hz, 2H, H-phenyl), 6.97 (d, *J* = 9 Hz, 2H, H-phenyl), 5.97 (s, 2H, H-pyrrole), 4.00 (t, *J* = 6 Hz, 2H, -O-CH_2_-), 3.74 (t, *J* = 7 Hz, 2H, -CH_2_-N-), 2.54 (s, 6H, -CH_3_), 1.92–1.84 (m, 2H), 1.83–1.64 (m, 2H), 1.61–1.52 (m, 2H), 1.43 (s, 6H, -CH_3_). ^13^C NMR (101 MHz, CDCl_3_) δ 168.4, 159.6, 155.2, 143.2, 141.9, 133.9, 132.1, 131.8, 129.1, 126.9, 123.2, 121.0, 115.0, 67.7, 37.8, 28.8, 28.4, 23.4, 14.6, 14.5. IR (ATR) ν 29505, 2862, 1772, 1703s, 1546, 1511 cm^−1^. UV-vis (MeOH) λ (ε) 498 nm (58,000 M^−1^ cm^−1^). HRMS: *m*/*z* (Δ ppm)—with the intensity as a percentage of the maximal value. Calcd for C_32_H_32_BF_2_N_3_O_3_ 555.2505. Found [M + H]^+^ 556.2581—9% (0.4 ppm), [M − F]^−^ 536.2533—36% (2.2 ppm), [M + Na]^+^ 578.2415—100% (2.2 ppm).

##### 1,3,5,7-Tetramethyl-8-{4-[8-(phthalimidyl)octyloxy]phenyl}-4,4-difluoro-4-bora-3a,4a-diaza-s-indacene (**2b**)

2,4-Dimethylpyrrole (0.62 mL, 6 mmol) and compound **1b** (1.14 g, 3 mmol) were dissolved in dichloromethane (100 mL), and 2 drops of trifluoroacetic acid were added. After 1 h, DDQ (1.36 g, 6 mmol) dissolved in dichloromethane (50 mL) was added. After another hour, DIPEA (7.84 mL, 45 mmol) was added, and BF_3_•Et_2_O (7.41 mL; 60 mmol) was added dropwise. The reaction mixture was stirred at room temperature for 6 h. Next, 150 mL of water was added, and the resulting two-phase mixture was Celite-filtrated and extracted with water (2 × 100 mL). The organic layer was collected, dried with anhydrous Na_2_SO_4_, and evaporated. Chromatographic separation was performed using silica gel and a hexane-dichloromethane mixture (1:1), followed by dichloromethane alone. The solid residue from evaporated fractions was dissolved in dichloromethane and crystallized using *n*-hexane to give an orange precipitate of **2b** (0.42 g, 23%), *R*_f_ = 0.42 (dichloromethane). ^1^H NMR (600 MHz, CDCl_3_) δ 7.84 (dd, *J* = 5, 3 Hz, 2H, H-phthalimidyl), 7.70 (dd, *J* = 5, 3 Hz, 2H, H-phthalimidyl), 7.14 (d, *J* = 8.5 Hz, 2H, H-phenyl), 6.98 (d, *J* = 8.5 Hz, 2H, H-phenyl), 5.96 (s, 2H, H-pyrrole), 3.99 (t, *J* = 7 Hz, 2H, -O-CH_2_-), 3.68 (t, *J* = 7 Hz, 2H, -CH_2_-N-), 2.54 (s, 6H, -CH_3_), 1.83–1.77 (m, 2H), 1.72–1.65 (m, 2H), 1.51–1.44 (m, 2H), 1.43 (s, 6H), 1.38 (s, 6H). ^13^C NMR (151 MHz, CDCl_3_) δ 168.5, 159.8, 155.3, 143.3, 142.1, 133.9, 132.3, 131.9, 129.2, 126.9, 123.2, 121.1, 115.2, 68.2, 38.1, 29.3, 29.1, 28.6, 26.8, 26.0, 14.6. IR (ATR) ν 2934, 2860, 1774, 1708s, 1550, 1524 cm^−1^. UV-vis (MeOH) λ (ε) 498 nm (92,000 M^−1^ cm^−1^). HRMS (ES): *m*/*z* (Δ ppm)—with the intensity as a percentage of the maximal value. Calcd for C_35_H_38_BF_2_N_3_O_3_ 597.2974. Found [M]^+•^ 597.2979—0.4% (0.8 ppm), [M − F]^−^ 578.2998—9% (1.4 ppm), [M + Na]^+^ 620.2882—86% (1.6 ppm), [M + K]^+^ 636.2621—0.5% (1.6 ppm).

##### 2,6-Diiodo-1,3,5,7-tetramethyl-8-{4-[5-(phthalimidyl)pentyloxy]phenyl}-4,4-difluoro-4-bora-3a,4a-diaza-s-indacene (**3a**)

Iodic acid (106 mg, 0.6 mmol) was dissolved in 1 mL of water and added to a solution of BODIPY **2a** (166 mg, 0.3 mmol) and iodine (115 mg, 0.45 mmol) in 30 mL of ethanol. The reaction was carried out at 70 °C for 30 min and evaporated to dryness. Chromatographic separation was performed using silica gel and a hexane-dichloromethane phase (1:1). The collected fractions were evaporated, and the dry residue was dissolved in dichloromethane and crystallized with *n*-pentane to give **3a** as a red-violet solid (165 mg, 69%), *R*_f_ = 0.47 (dichloromethane). ^1^H NMR (500 MHz, CDCl_3_) δ 7.86 (dd, *J* = 5, 3 Hz, 2H, H-phthalimidyl), 7.73 (dd, *J* = 5, 3 Hz, 2H, H-phthalimidyl), 7.11 (d, *J* = 9 Hz, 2H, H-phenyl), 7.00 (d, *J* = 9 Hz, 2H, H-phenyl), 4.02 (t, *J* = 6 Hz, 2H, -O-CH_2_-), 3.75 (t, *J* = 7 Hz, 2H, -CH_2_-N-), 2.64 (s, 6H, -CH_3_), 1.93–1.86 (m, 2H), 1.84–1.76 (m, 2H), 1.62–1.57 (m, 2H), 1.44 (s, 6H, -CH_3_). ^13^C NMR (126 MHz, CDCl_3_) δ 168.5, 160.0, 156.5, 145.4, 141.7, 134.0, 132.1,131.8, 129.0, 126.5, 123.2, 115.4, 67.8, 37.8, 28.8, 28.4, 23.4, 17.2, 16.0. IR (ATR) ν 2944, 1771, 1706s, 1527 cm^−1^. UV-vis (MeOH) λ (ε) 530 nm (23,000 M^−1^ cm^−1^). HRMS (ES): *m*/*z* (Δ ppm)—with the intensity as a percentage of the maximal value. Calcd for C_32_H_30_BF_2_I_2_N_3_O_3_ 807.0438. Found [M]^+•^ 807.0422—10% (2.0 ppm), [M − F]^−^ 788.0437—35% (2.2 ppm), [M + Na]^+^ 830.0319—100% (1.9 ppm).

##### 2,6-Diiodo-1,3,5,7-tetramethyl-8-{4-[8-(phthalimidyl)octyloxy]phenyl}-4,4-difluoro-4-bora-3a,4a-diaza-s-indacene (**3b**)

Method 1: Iodic acid (70 mg, 0.4 mmol) was dissolved in 1 mL of water and added to a solution of BODIPY **2b** (119 mg, 0.2 mmol) and iodine (76 mg, 0.4 mmol) in 20 mL of ethanol. The reaction was stirred at 70 °C for 30 min and evaporated to dryness. Chromatographic separation was performed using silica gel and a hexane-dichloromethane phase (1:1). The collected fractions were evaporated, and the dry residue was dissolved in dichloromethane and crystallized with *n*-hexane to give **3b** as a red-violet solid (104 mg, 61%)

Method 2: BODIPY **2b** (60 mg, 0.1 mmol) and *N*-iodosuccinimide (NIS) (56 mg, 0.25 mmol) were dissolved in 10 mL of dichloromethane and stirred in the dark at room temperature for 3 h. The reaction mixture was extracted with water (30 mL), dried with anhydrous Na_2_SO_4_, and evaporated to dryness. Chromatographic separation was performed using silica gel and a hexane-dichloromethane phase (4:1). The collected fractions were evaporated, and the resulting solid was dissolved in dichloromethane and crystallized with *n*-hexane to give **3b** (67 mg, 79%), *R*_f_ = 0.67 (dichloromethane). ^1^H NMR (600 MHz, CDCl_3_) δ 7.84 (dd, *J* = 5, 3 Hz, 2H, H-phthalimidyl), 7.71 (dd, *J* = 5, 3 Hz, 2H, H-phthalimidyl), 7.11 (d, *J* = 9 Hz, 2H, H-phenyl), 7.01 (d, *J* = 9 Hz, 2H, H-phenyl), 4.01 (t, *J* = 7 Hz, 2H, -O-CH_2_-), 3.69 (t, *J* = 7 Hz, 2H, -CH_2_-N-), 2.63 (s, 6H, -CH_3_), 1.84–1.79 (m, 2H), 1.72–1.66 (m, 2H), 1.52–1.46 (m, 2H), 1.44 (s, 6H), 1.39 (s, 6H). ^13^C NMR (151 MHz, CDCl_3_) δ 168.5, 160.2, 156.6, 145.5, 141.8, 133.9, 132.3, 131.8, 129.1, 126.5, 123.2, 115.5, 85.6, 68.3, 38.1, 29.3, 29.2, 29.1, 28.6, 26.8, 26.0, 17.3, 16.1. IR (ATR) ν 2946, 2853, 1769, 1709s, 1538, 1524 cm^−1^. UV-vis (MeOH) λ (ε) 530 nm (5000 M^−1^ cm^−1^). HRMS (ES): *m*/*z* (Δ ppm)—with the intensity as a percentage of the maximal value. Calcd for C_35_H_36_BF_2_I_2_N_3_O_3_ 849.0907. Found [M]^+•^ 849.0899—0.5% (0.9 ppm), [M + Na]^+^ 872.0790—0.6% (1.7 ppm), [M − F]^−^ 830.0908—2.5% (1.8 ppm).

##### 2,6-Dibromo-1,3,5,7-tetramethyl-8-{4-[5-(phthalimidyl)pentyloxy]phenyl}-4,4-difluoro-4-bora-3a,4a-diaza-s-indacene (**4a**)

BODIPY **2a** (222 mg, 0.4 mmol) was dissolved in 30 mL of a dichloromethane and dimethylformamide mixture (1:1). Next, the *N*-bromosuccinimide (NBS) (356 mg, 2.0 mmol) in 15 mL of dichloromethane was added dropwise, and the reaction mixture was stirred at room temperature for 7 h. The mixture was extracted with water (2 × 100 mL). The organic layer was dried with anhydrous MgSO_4_ and evaporated. The solid residue was chromatographed using silica gel and a hexane-dichloromethane mixture (from 1:1 to 4:1). The collected fractions were evaporated, then dissolved in dichloromethane and crystallized with *n*-pentane to give a red precipitate of **4a** (55 mg, 19%), *R*_f_ = 0.43 (dichloromethane). ^1^H NMR (500 MHz, CDCl_3_) δ 7.85 (dd, *J* = 5, 3 Hz, 2H, H-phthalimidyl), 7.72 (dd, *J* = 5, 3 Hz, 2H, H-phthalimidyl), 7.11 (d, *J* = 9 Hz, 2H, H-phenyl), 7.00 (d, *J* = 9 Hz, 2H, H-phenyl), 3.75 (t, *J* = 6 Hz, 2H, -O-CH_2_-), 3.75 (t, *J* = 7 Hz, 2H, -CH_2_-N-), 2.66–2.56 (m, 6H), 1.93–1.86 (m, 2H), 1.86–1.73 (m, 2H), 1.62–1.56 (m, 2H), 1.47–1.41 (m, 6H, -CH_3_). ^13^C NMR (126 MHz, CDCl_3_) δ 168.5, 160.0, 153.7, 142.4, 140.7, 134.0, 132.1, 130.8, 129.7, 129.1, 126.2, 124.4, 123.2, 115.3, 111.7, 67.8, 37.8, 28.8, 28.4, 23.4, 14.4, 13.9, 13.7. IR (ATR) ν 2938, 1775, 1710s, 1539 cm^−1^. UV-vis (MeOH) λ (ε) 525 nm (52,000 M^−1^ cm^−1^). HRMS (ES): *m*/*z* (Δ ppm)—with the intensity as a percentage of the maximal value. Calcd for C_32_H_30_BBr_2_F_2_N_3_O_3_ 712.0793. Found [M + H]^+^ 712.0787—1% (0.8 ppm), [M + Na] 734.0611—51% (0.3 ppm).

##### 2,6-Dibromo-1,3,5,7-Tetramethyl-8-{4-[8-(phthalimidyl)octyloxy]phenyl}-4,4-difluoro-4-bora-3a,4a-diaza-s-indacene (**4b**)

BODIPY **2b** (119 mg, 0.2 mmol) was dissolved in 15 mL of dichloromethane, and NBS (107 mg, 0.6 mmol) in dichloromethane (8 mL) was added dropwise. The reaction mixture was stirred at room temperature for 17 h and then extracted with brine (30 mL). The organic layer was dried with anhydrous Na_2_SO_4_ and evaporated. The solid residue was chromatographed using silica gel and a hexane-dichloromethane phase (4:1). The collected fractions were evaporated, then dissolved in dichloromethane and crystallized with *n*-hexane to give a red precipitate of **4b** (80 mg, 53%), *R*_f_ = 0.65 (dichloromethane). ^1^H NMR (600 MHz, CDCl_3_) δ 7.84 (dd, *J* = 5, 3 Hz, 2H, H-phthalimidyl), 7.70 (dd, *J* = 5, 3 Hz, 2H, H-phthalimidyl), 7.11 (d, *J* = 9 Hz, 2H, H-phenyl), 7.01 (d, *J* = 9 Hz, 2H, H-phenyl), 4.01 (t, *J* = 7 Hz, 2H, -O-CH_2_-), 3.69 (t, *J* = 7 Hz, 2H, -CH_2_-N-), 2.60 (s, 6H), 1.85–1.79 (m, 2H), 1.72–1.66 (m, 2H), 1.51–1.46 (m, 2H), 1.42 (s, 6H), 1.39 (s, 6H). ^13^C NMR (151 MHz, CDCl_3_) δ 168.5, 160.2, 153.7, 142.6, 140.8, 133.9, 132.3, 130.9, 129.1, 126.2., 123.2, 115.4, 111.7, 68.3, 38.1, 29.3, 29.2, 29.1, 28.6, 26.8, 26.0, 14.0, 13.7. IR (ATR) ν 2942, 1768, 1708s, 1535 cm^−1^. UV-vis (MeOH) λ (ε) 523 nm (11,000 M^−1^ cm^−1^). HRMS (ES): *m*/*z* (Δ ppm)—with the intensity as a percentage of the maximal value. Calcd for C_35_H_36_BBr_2_F_2_N_3_O_3_ 753.1185. Found [M]^+•^ 753.1185—0.04% (0 ppm), [M + Na]^+^ 776.1082—0.2% (0 ppm), [M−F]^−^ 734.1202—0.4% (0.1 ppm), [M + K]^+^ 792.0849—0.02% (3.4 ppm).

##### 1,3,5,7-Tetramethyl-8-[4-(5-aminopentyloxy)phenyl]-4,4-difluoro-4-bora-3a,4a-diaza-s-indacene (**5a**)

BODIPY **2a** (222 mg, 0.4 mmol) was dissolved in 30 mL of ethanol, followed by the addition of hydrazine monohydrate (0.92 mL, 19 mmol). The reaction mixture was refluxed for 6 h, then cooled to about 5 °C and filtered to remove the white precipitate that appeared. The filtrate was evaporated and chromatographed using aluminium oxide gel and a dichloromethane-methanol phase (from 10:1 to 4:1). Evaporated fractions were crystalized from ethyl acetate–*n*-pentane to give **5a** as a brown solid (22 mg, 13%), *R*_f_ = 0.15 (dichloromethane-methanol, 4:1). ^1^H NMR (600 MHz, CD_3_OD) δ 7.18 (d, *J* = 9 Hz, 2H, H-phenyl), 7.08 (d, *J* = 9 Hz, 2H, H-phenyl), 6.05 (s, 2H, H-pyrrole), 4.08 (t, *J* = 6 Hz, 2H, -O-CH_2_-), 3.01–2.95 (m, 2H, -CH_2_NH_2_), 2.48 (s, 6H, -CH_3_), 1.91–1.86 (m, 2H), 1.80–1.74 m, 2H), 1.66–1.60 (m, 2H), 1.44 (s, 6H). ^13^C NMR (151 MHz, CD_3_OD) δ 160.0, 155.1, 143.2, 142.4, 131.7, 129.2, 126.8, 120.8, 115.0, 67.4, 39.4, 28.5, 27.1, 22.9, 13.4, 13.2. IR (ATR) ν 1929, 1609, 1544, 1509 cm^−1^. UV-vis (MeOH) λ (ε) 498 nm (79,000 M^−1^ cm^−1^). HRMS (ES): *m*/*z* (Δ ppm)—with the intensity as a percentage of the maximal value. Calcd for C_24_H_30_BF_2_N_3_O 426.2528. Found [M + H]^+^ 426.2529—100% (0.2 ppm), [M − F]^−^ 406.2466—54% (0 ppm).

##### 1,3,5,7-Tetramethyl-8-[4-(8-aminooctyloxy)phenyl]-4,4-difluoro-4-bora-3a,4a-diaza-s-indacene (**5b**)

BODIPY **2b** (119 mg, 0.2 mmol) was dissolved in ethanol (15 mL), and then hydrazine monohydrate (0.46 mL, 9.5 mmol) was added. The reaction was refluxed for 6 h, then cooled to about 5 °C and filtered to remove the white precipitate that appeared. The filtrate was evaporated and chromatographed using aluminium oxide gel and dichloromethane-methanol phase 4:1. Evaporated fractions were crystallized from ethyl acetate–*n*-hexane to give **5b** as a brown solid (52 mg, 56%), *R*_f_ = 0.26 (dichloromethane-methanol, 4:1).^1^H NMR (600 MHz, MeOD) δ 7.16 (d, *J* = 9 Hz, 2H, H-phenyl), 7.06 (d, *J* = 9 Hz, 2H, H-phenyl), 6.05 (s, 2H, H-pyrrole), 4.04 (t, *J* = 6 Hz, 2H, -O-CH_2_-), 2.93 (d, *J* = 8 Hz, 2H), 2.47 (s, 6H), 1.85–1.78 (m, 2H), 1.71–1.65 (m, 2H), 1.56–1.49 (m, 2H), 1.45 (s, 6H), 1.43 (s, 6H). ^13^C NMR (151 MHz, CD_3_OD) δ 161.4, 156.4, 144.6, 143.8, 133.0, 130.75, 128.0, 122.1, 116.4, 69.2, 40.8, 30.3, 30.1, 28.6, 27.4, 27.1, 14.7, 14.5. IR (ATR) ν 2929, 2860, 1544, 1509 cm^−1^. UV-vis (MeOH) λ (ε) 498 nm (10,000 M^−1^ cm^−1^). HRMS (ES): *m*/*z* (Δ ppm)—with the intensity as a percentage of the maximal value. Calcd for C_27_H_36_BF_2_N_3_O 468.2998. Found [M + H]^+^ 468.2997—40% (0.4 ppm), [M − F]^−^ 448.2934—100% (0.5 ppm), [M + Na]^+^ 490.2810—0.3% (1.4 ppm).

### 3.2. Photophysical Measurements

The singlet oxygen quantum yields (*Φ*_Δ_) and the fluorescence quantum yields (*Φ*_F_) of tested samples in MeOH were determined using published comparative (relative) methods [40] with rhodamin-G6 used as a reference for fluorescence (*Φ*_F_ = 0.94 in EtOH [31]), and bengal rose as a reference for singlet oxygen determinations (*Φ*_Δ_ = 0.76 in MeOH [32]). Singlet oxygen measurements were performed with 1,3-diphenylisobenzofuran as the chemical scavenger of singlet oxygen by monitoring its decomposition at 417 nm. The *Φ*_F_ values were also determined by an absolute method using an integrating sphere in an FLS1000 fluorimeter (Edinburgh Instruments, Edinburgh, UK). Fluorescence lifetimes were measured on the same fluorimeter upon excitation by HPL510 laser (Edinburgh Instruments, λ = 506.1 nm).

### 3.3. Photobleaching Experiments

The photobleaching experiments were performed in a serum-containing cell culture medium at a concentration of 10 µM. The samples were prepared by direct dilution from the 1 mM DMSO stock solution (0.5 mM in case of **4b**). The quartz cuvette with the sample (2.5 mL) was irradiated by the 500W halogen lamp (Tip) from a distance of 60 cm at room temperature. The light was filtered through a 7 cm water filter to eliminate heat. The absorption spectra were collected every 10 min. The experiment was performed in triplicate.

### 3.4. Cell Culture and Sample Preparation

The human cervical carcinoma (HeLa) and human melanoma (SK-MEL-28) cell lines were purchased from the American Type Cell Culture Collection (ATCC; Manassas, VA, USA). Both cell lines were cultured in Dulbecco’s modified Eagle’s medium (DMEM) without phenol red (Lonza, Verviers, Belgium) and supplemented with 10% heat-inactivated fetal bovine serum (Sigma-Aldrich (Merck, Darmstadt, Germany), 1% penicillin/streptomycin solution (Lonza), 10 mM HEPES buffer (Lonza), and 4 mM ultraglutamine I (alanyl-*L*-glutamine; Lonza). The cell lines were cultured in 75 cm^2^ tissue culture flasks (TPP, Trasadingen, Switzerland) and maintained in a CO_2_ incubator at 37 °C in a humidified atmosphere of 5% CO_2_ and subcultured every 3−4 days. For the cytotoxicity experiments (phototoxicity and dark toxicity), cells were seeded into 96-well plates (TPP) at a density of 7.5 × 10^3^ (HeLa and SK-MEL-28) cells per well for 24 h.

Stock solutions of the investigated compounds were prepared in DMSO at a concentration of 0.5 mM (**4b**) or 1 mM (all other compounds) and were sterilized by filtration through 0.22 μm syringe MCE filters. The stock solutions were diluted by the above-mentioned cell culture medium to the required concentrations. The maximum DMSO concentration in the medium in the experiments was 1% (2% in case of cpd. **4b**). Cytoxicity of DMSO at corresponding concentrations (without studied compounds) was assessed in all cytotoxicity experiments as well—no toxicity against SK-MEL-28 or HeLa cells was observed.

### 3.5. Cytotoxicity Experiments

To assess dark toxicities (inherent toxicities of the studied compounds without irradiation), SK-MEL-28 cells were treated with studied PSs for 24 h in the dark. Viability was evaluated after two washing steps using the Neutral Red (NR; Sigma-Aldrich (Merck, Darmstadt, Germany) uptake assay, based on the ability of the living cells to incorporate NR into their intact lysosomes (concentration of NR in cell culture medium was 30 μg/mL and incubation time with NR was 2 h). The absorbance of soluble NR in cell lysate was measured at λ = 540 nm using a Tecan Infinite 200 M plate reader (Tecan, Männedorf, Switzerland). The viability of each experimental group was expressed as the percentage of the untreated controls incubated under the same conditions (100%). After staining (before lysis), the cells were briefly investigated under the microscope to verify changes in cellular morphology and the presence of NR in lysosomes (orange-red spots in the cells). These observations were in agreement with NR measurements.

For the photodynamic activity assessment experiments (phototoxicity), both HeLa and SK-MEL-28 cells were incubated overnight (12 h) with the studied compounds. The cells were then carefully and swiftly washed twice with a prewarmed sterile phosphate-buffered saline, fresh cell culture medium was added, and cells were subsequently irradiated for 15 min using a 450 W ozone-free Xe lamp (Newport; intensity reduced to 400 W). The lamp was equipped with a long-pass filter (Newport 20CGA-455) and an 8-cm water filter to cut off undesirable wavelengths and heat radiation, respectively (λ > 455 nm, 15.3 mW cm^−2^, 15 min, 13.7 J cm^−2^). Cellular viability was measured after an additional 24 h by the above-mentioned NR uptake assay. At least four independent experiments, each in triplicate, were performed.

The concentrations of the studied compounds that induced a 50% decrease in cellular viability after treatment under the dark conditions (TC_50_, the median toxic concentration) or after the photodynamic treatment (EC_50_, the median effective concentration) were calculated using GraphPad Prism software (version 9.1.2; GraphPad Software, Inc., San Diego, CA, USA) for each independent experiment. The data are presented as the means ± standard deviations of these values.

### 3.6. Subcellular Localization

SK-MEL-28 cells were seeded on Petri dishes suitable for confocal microscopy (WilCo Wells, Amsterdam, The Netherlands) at a density of 7.5 × 10^4^ cells per dish. Incubation with PSs in SCM (1 μM, 2 mL) was performed for 12 h in an incubator at 37 °C with constant humidity and a 5% CO_2_ atmosphere. The medium was removed, and cells were treated according to A), B) or C) as follows. For A) cells were washed twice with prewarmed serum-free medium (SFM) and fresh SFM containing 0.4 μM MitoTracker™ Deep red (Molecular Probes, Thermo Fisher Scientific, Waltham, MA, USA; further referred as “MitoTracker”) and 0.4 μM LysoTracker™ Red (Molecular Probes; further referred as “LysoTracker”) and 10 nM Hoechst 33342 (Molecular Probes; further referred as “Hoechst”) was added for 20 min, then cells were washed twice with SFM and fresh SFM was added. For B) cells were washed as described above and fresh SFM containing 1 μM ER-Tracker Red and 10 nM Hoechst was added for 20 min and then cells were washed as described above and fresh SFM was added. For C) cells were fixed by 4% formaldehyde for 15 min and then washed three times with PBS. Then cells were incubated in PBS containing 10 nM Hoechst and 1× HCS LipidTOX™ Deep Red Neutral Lipid Stain (Molecular Probes; further referred as “LipidTOX”) at room temperature for 30 min. During the incubation time, petri dishes were sealed with plate-sealing film. Specimens were examined with a Nikon Ti-E microscope (Nikon, Tokyo, Japan) and 100× oil immersion objective lens (Nikon CFI Plan Apochromat Lambda 100× Oil, N.A. 1.45). Samples stained with method A or B were placed in a stage-top CO_2_ incubator (Okolab, Italy) during imaging. Microphotographs were taken with an Andor Zyla 5.5 sCMOS camera (Andor Technology, Belfast, United Kingdom) using FITC (PSs), DAPI (Hoechst 33342), Cy3 (LysoTracker and ER-Tracker) and Cy5 (MitoTracker and LipidTOX) filter sets.

### 3.7. Real-Time Monitoring of Annexin V Binding and the Loss of Cell Membrane Integrity

A RealTime-Glo™ Annexin V Apoptosis and Necrosis Assay (Promega, Madison, WI, USA) was utilized for the real-time assessment of cell death. This monitors phosphatidylserine exposure on the outer leaflet of the cytoplasmic membrane (increase in luminescence signal) together with binding of the membrane-impermeable probe to DNA (increase in fluorescence signal) that accompanies a loss of membrane integrity, typical for late stages of apoptosis (secondary necrosis) or necrotic type of cell death. According to the manufacturer, this assay uses Annexin V fusion proteins containing complementary subunits of luciferase (Annexin V-LgBiT and Annexin V-SmBiT). The luminescence signal is low until both subunits bind to phosphatidylserine, and in close vicinity form functional luciferase.

For this assay, SK-MEL-28 cells were seeded onto 96-well white clear-bottom plates (Corning, New York, NY, USA) in a 1:1 mixture of DMEM and CO_2_ independent medium (Gibco; supplemented with 10% FBS, 1% penicillin/streptomycin solution and 4 mM ultraglutamine I). The next day, the medium was replaced with a fresh CO_2_ independent medium, and cells were incubated overnight. The next day, studied PSs dissolved in a CO_2_ independent medium in concentrations corresponding to their EC_85_ were added as described above. The cells were washed with 100 μL of fresh medium, and the whole plate was irradiated as described above using a Xe-lamp (λ > 455 nm, 15.3 mW cm^−2^, 15 min, 13.7 J cm^−2^). A 2× detection reagent was prepared by a 500-fold dilution of each component (Annexin V NanoBiT™ Substrate, CaCl_2_, Necrosis Detection Reagent, Annexin V-SmBiT, and Annexin V-LgBiT) in a CO_2_ independent medium ad hoc (during irradiation of samples) according to the manufacturer’s protocol. After irradiation, positive controls were added to non-treated wells and 100 μL of the 2× detection reagent was immediately added to each well. The whole 96-well plate was put on a vibrating platform shaker (Heidolph Instruments, Schwabach, Germany) for 30 s at 650 rpm to mix. After mixing, samples were placed into a plate reader with a temperature control (Tecan Spark, Männedorf, Switzerland) and measured each 15 min for 24 h—luminescence (200 ms integration time) and fluorescence (λ_exc_ = 485 nm; λ_emi_ = 530 nm, BP_exc/emi_ = 20 nm; 10 flashes per well, 40 μs integration time).

Digitonin (25 μg/mL), TRAIL (400 ng/mL), and paclitaxel (1 μM) were used as positive controls for membrane-disturbing-agent induced necrosis, extrinsic-pathway-mediated apoptosis, and intrinsic-pathway-mediated apoptosis, respectively. Cell-free wells (no-cell controls) were used to subtract background luminescence and fluorescence for each time point. Results are plotted as normalized signals.

## 4. Conclusions

In conclusion, two series of (non)halogenated BODIPY dyes bearing a phthalimido or amino substituent on the phenoxyoctyl or phenoxypentyl linker in the *meso* position were synthesized and characterized. The photophysical assessment of these dyes clearly indicated a significant heavy atom effect of the halogens in positions 2 and 6. While strong singlet oxygen production (*Φ*_Δ_ ~ 0.76) and weak fluorescence (*Φ*_Δ_ ~ 0.03) was observed for diiodinated compounds, data completely the reverse were determined for 2,6-unsubstituted BODIPY dyes (*Φ*_Δ_ ~ 0.06, *Φ*_F_ ~ 0.47). For the dibromo substituted BODIPYs, their photophysical properties were located just between (*Φ*_Δ_ ~ 0.56, *Φ*_F_ ~ 0.21). The photophysics was not affected by the length of the linker or the end modification (phthalimido or amino). All the dyes absorbed in the region around 500 nm, which predestines them for the photodynamic treatment of superficial lesions, where deep penetration of light is not required. This has been confirmed on the melanoma skin cancer cell line SK-MEL-28, where photodynamic activities followed the rate of singlet oxygen production, with the best result for diiodinated **3a** exerting an EC_50_ = 0.052 ± 0.01 µM. The lowest activity was found for the non-halogenated derivatives. The data were confirmed by similar trends on HeLa cells. Owning to their lipophilic character, all studied BODIPY derivatives were predominantly found in adiposomes (lipid droplets) with further lower signals colocalized in either endolysosomal vesicles or the endoplasmic reticulum. Irradiation of SK-MEL-28 cells triggered PDT-induced apoptosis observed for all studied compounds. At the end, it may be concluded that the presence of halogens in positions 2 and 6 of BODIPY dyes is a required prerequisite for their efficient use as PSs in PDT.

## Figures and Tables

**Table 1:** Photophysical data of studied compounds in MeOH ^a^.

Cpd.	X	*λ*_max_, nm (log ε)	*λ*_F_, nm	*Φ*_F (absol)_	*Φ*_F (relat)_^b^	*τ*_F_, ns	*Φ*_Δ_^c^
**2a**	-H	498 (4.77)	507	0.52	0.49	3.27	0.036
**3a**	-I	530 (4.36)	546	0.022	0.024	0.17	0.77
**4a**	-Br	525 (4.72)	540	0.21	0.19	1.37	0.57
**5a**	-H	498 (4.90)	507	0.43	0.44	3.25	0.063
**2b**	-H	498 (4.96)	507	0.44	0.42	2.88	0.057
**3b**	-I	530 (3.70)	547	0.030	0.023	0.17	0.75
**4b**	-Br	523 (4.04)	538	0.23	0.22	1.54	0.56
**5b**	-H	498 (4.00)	507	0.50	0.47	3.22	0.071

^a^ absorption maximum (*λ*_max_), extinction coefficient (ε), emission maximum (*λ*_F_), fluorescence quantum yield determined by relative method (*Φ*_F(relat)_), fluorescence quantum yield determined by absolute method (*Φ*_F(absol)_), fluorescence lifetime (*τ*_F_, λ_exc_ = 506.1 nm), singlet oxygen quantum yield (*Φ*_Δ_); ^b^ Rhodamin-G6 used as reference (*Φ*_F_ = 0.94 in EtOH [31]); ^c^ Bengal rose used as reference (*Φ*_Δ_ = 0.76 in MeOH [32]).

**Table 2:** Comparison of photodynamic parameters of the studied BODIPY dyes and structurally different PSs ^a^.

Cpd.	X	EC_50_ (μM)			TC_50_ (μM)	TC_50_/EC_50_
		HeLa	SK-MEL-28		SK-MEL-28	SK-MEL-28
**2a**	-H	3.60 ± 0.19	2.55 ± 0.14		>10^b^	>3.92
**3a**	-I	0.14 ± 0.01	0.052 ± 0.01		>10 ^b^	>192
**4a**	-Br	0.21 ± 0.02	0.081 ± 0.023		>10 ^b^	>123
**5a**	-H	9.4 ± 0.70	3.6 ± 0.66		>10 ^b^	>2.78
**2b**	-H	2.4 ± 0.53	3.79 ± 0.20		>10 ^b^	>2.64
**3b**	-I	1.57 ± 0.33	1.23 ± 0.14		>10 ^b^	>8.13
**4b**	-Br	0.37 ± 0.07	0.22 ± 0.05		>10 ^b^	>45.5
**5b**	-H	1.57 ± 0.33	2.42 ± 0.46		>10 ^b^	>4.13
Methylene blue ^c^		5.15 ± 1.93			22.7 ± 7.9 ^d^	4.4 ^d^
S_3_AlOHPc ^c^		2.07 ± 0.29			127 ± 7.8 ^d^	60 ^d^
Pp IX ^c^		0.80 ± 0.13			>10 ^b,d^	>10 ^d^
temoporfin ^c^		0.045 ± 0.007			20.6 ± 2.5 ^d^	460 ^d^

^a^ Dark toxicity (TC_50_ values), Photodynamic activity (EC_50_ values), Phototherapeutic indices (TC_50_/EC_50_); Data are presented as the TC_50_ or EC_50_ values ± standard deviations. At least four independent experiments each in triplicate were performed. Irradiation conditions: λ > 455 nm, 15.3 mW cm^−2^, 15 min, 13.7 J cm^−2^; ^b^ The compounds precipitated at concentrations over the listed values; ^c^ Data from [37] (λ > 570 nm, 12.4 mW cm^−2^, 15 min, 11.2 J cm^−2^); ^d^ on HeLa cells.

## Data Availability

The data presented in this study are available in article or supplementary material.

## Supplementary Materials

The following are available online, NMR spectra of the compounds. Absorption, emission, and excitation spectra. Graphs with biological activities. Graphs with the determination of cell death. Figures with subcellular localization.

## Sample Availability

Sample of componds **2a,b**–**5a,b** are available from the authors.
