# Supplementary material for: Tuning Photodynamic Properties of BODIPY Dyes, Porphyrins’ Little Sisters
Source: Molecules. 2021 Jul 10;26(14):4194. doi: 10.3390/molecules26144194 (PMC8305389; doi:10.3390/molecules26144194)
Supplement: Supplementary file 1 [file molecules-26-04194-s001.zip › molecules-1246504-supplementary.pdf]

Supplementary Materials

Tuning Photodynamic Properties of BODIPY Dyes, Porphyrins´ Little Sisters

Wojciech Krzemien [1,2], Monika Rohlickova [2], Miloslav Machacek [2], Veronika Novakova [2], Jaroslaw Piskorz [1*] and Petr Zimcik [2*]

[1] Chair and Department of Inorganic and Analytical Chemistry, Poznan University of Medical Sciences, Grunwaldzka 6, 60-780 Poznan, Poland

[2] Faculty of Pharmacy in Hradec Kralove, Charles University, Ak. Heyrovskeho 1203, 50003, Hradec Kralove, Czech Republic

* Correspondence: piskorzj@ump.edu.pl, zimcik@faf.cuni.cz;

Content

Content S2

NMR spectra S4

4-[5-(1,3-Dihydro-1,3-dioxo-2*H*-isoindol-2-yl)pentyloxy]benzaldehyde (**1a**) S4

4-[8-(1,3-Dihydro-1,3-dioxo-2*H*-isoindol-2-yl)octyloxy]benzaldehyde (**1b**) S6

1,3,5,7-Tetramethyl-8-{4-[5-(phthalimidyl)pentyloxy]phenyl}-4,4-difluoro-4-bora-3a,4a-diaza-s-indacene (**2a**) S8

1,3,5,7-Tetramethyl-8-{4-[8-(phthalimidyl)octyloxy]phenyl}-4,4-difluoro-4-bora-3a,4a-diaza-s-indacene (**2b**) S10

2,6-Diiodo-1,3,5,7-tetramethyl-8-{4-[5-(phthalimidyl)pentyloxy]phenyl}-4,4-difluoro-4-bora-3a,4a-diaza-s-indacene (**3a**) S12

2,6-Diiodo-1,3,5,7-tetramethyl-8-{4-[8-(phthalimidyl)octyloxy]phenyl}-4,4-difluoro-4-bora-3a,4a-diaza-s-indacene (**3b**) S14

2,6-Dibromo-1,3,5,7-tetramethyl-8-{4-[5-(phthalimidyl)pentyloxy]phenyl}-4,4-difluoro-4-bora-3a,4a-diaza-s-indacene (**4a**) S16

2,6-Dibromo-1,3,5,7-tetramethyl-8-{4-[8-(phthalimidyl)octyloxy]phenyl}-4,4-difluoro-4-bora-3a,4a-diaza-s-indacene (**4b**) S18

1,3,5,7-Tetramethyl-8-[4-(5-aminopentyloxy)phenyl]-4,4-difluoro-4-bora-3a,4a-diaza-s-indacene (**5a**) S20

1,3,5,7-Tetramethyl-8-[4-(8-aminooctyloxy)phenyl]-4,4-difluoro-4-bora-3a,4a-diaza-s-indacene (**5b**) S22

HRMS spectra S24

4-[5-(1,3-Dihydro-1,3-dioxo-2*H*-isoindol-2-yl)pentyloxy]benzaldehyde (**1a**) S24

4-[8-(1,3-Dihydro-1,3-dioxo-2*H*-isoindol-2-yl)octyloxy]benzaldehyde (**1b**) S25

1,3,5,7-Tetramethyl-8-{4-[5-(phthalimidyl)pentyloxy]phenyl}-4,4-difluoro-4-bora-3a,4a-diaza-s-indacene (**2a**) S26

1,3,5,7-Tetramethyl-8-{4-[8-(phthalimidyl)octyloxy]phenyl}-4,4-difluoro-4-bora-3a,4a-diaza-s-indacene (**2b**) S27

2,6-Diiodo-1,3,5,7-tetramethyl-8-{4-[5-(phthalimidyl)pentyloxy]phenyl}-4,4-difluoro-4-bora-3a,4a-diaza-s-indacene (**3a**) S28

2,6-Diiodo-1,3,5,7-tetramethyl-8-{4-[8-(phthalimidyl)octyloxy]phenyl}-4,4-difluoro-4-bora-3a,4a-diaza-s-indacene (**3b**) S29

2,6-Dibromo-1,3,5,7-tetramethyl-8-{4-[5-(phthalimidyl)pentyloxy]phenyl}-4,4-difluoro-4-bora-3a,4a-diaza-s-indacene (**4a**) S30

2,6-Dibromo-1,3,5,7-tetramethyl-8-{4-[8-(phthalimidyl)octyloxy]phenyl}-4,4-difluoro-4-bora-3a,4a-diaza-s-indacene (**4b**) S31

1,3,5,7-Tetramethyl-8-[4-(5-aminopentyloxy)phenyl]-4,4-difluoro-4-bora-3a,4a-diaza-s-indacene (**5a**) S32

1,3,5,7-Tetramethyl-8-[4-(8-aminooctyloxy)phenyl]-4,4-difluoro-4-bora-3a,4a-diaza-s-indacene (**5b**) S33

Absorption, emission, excitation spectra S34

Assessment of biological activity S36

Subcellular localization S37

Assessment of cell death S39

NMR spectra

4-[5-(1,3-Dihydro-1,3-dioxo-2*H*-isoindol-2-yl)pentyloxy]benzaldehyde (**1a**)

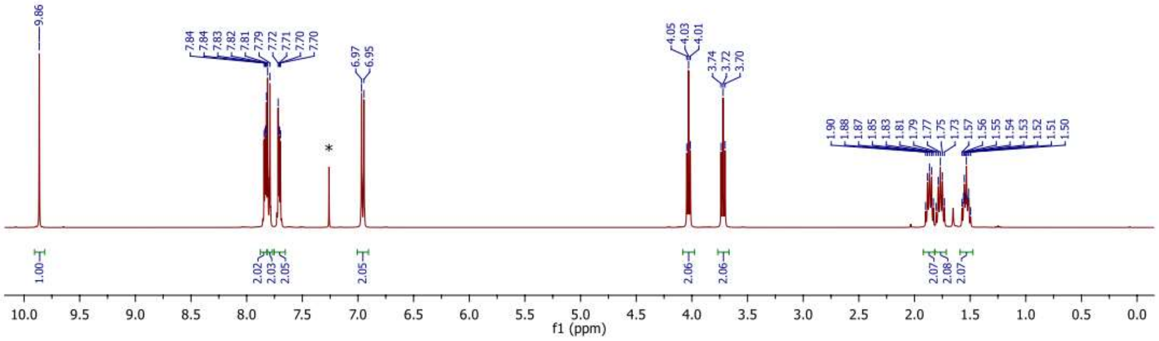

^{1}H NMR spectrum of **1a** (400 MHz, $CDCl_3$). The symbol * indicates chloroform residual peak.

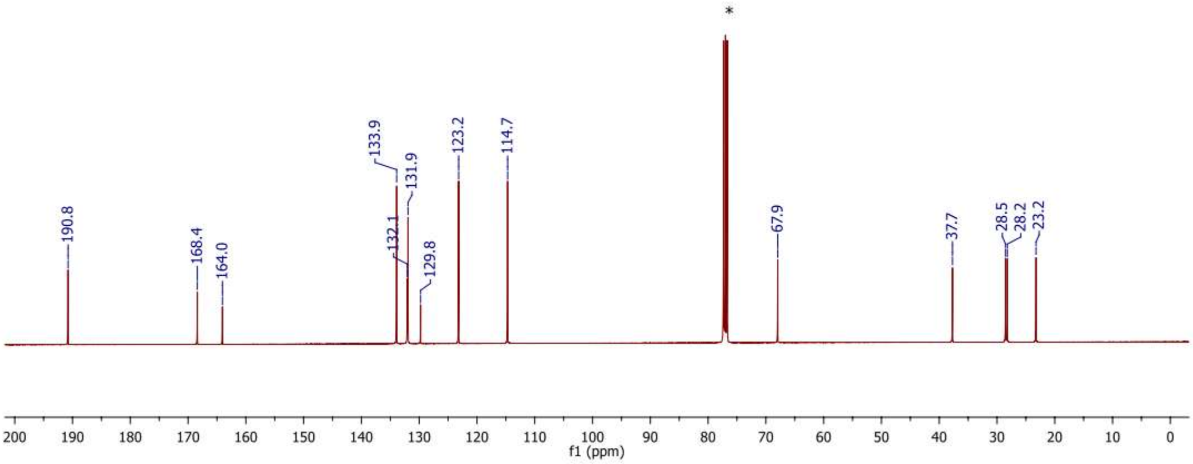

^{13}C NMR spectrum of **1a** (100 MHz, $CDCl_3$). The symbol * indicates chloroform residual peak.

Table S1. ^{1}H and ^{13}C NMR data obtained for **1a** including key correlations determined from ^{1}H-^{1}H COSY, ^{1}H-^{13}C HSQC and ^{1}H-^{13}C HMBC spectra.

δ_H [ppm]	Multiplicity (J_{H-H} w Hz)	^{1}H-^{1}H COSY δ_H [ppm]	^{1}H-^{13}C HSQC δ_C [ppm]	^{1}H-^{13}C HMBC δ_C [ppm]
1.59-1.47	m	1.81-1.72, 1.91-1.82	23.2	28.2, 28.5, 67.9, 37.7
1.81-1.72	m	1.59-1.47, 3.72	28.2	23.2, 28,2, 28,5, 37.7
1.91-1.82	m	1.59-1.47, 4.03	28.5	23.2, 28.2, 28.5, 67.9
3.72	t (7)	1.81-1.72	37.7	23.2, 28.2, 28.5, 168.4
4.03	t (6)	1.91-1.82	67.9	23.2, 28.2, 28.5, 164.0
6.96	d (9)	7.80	114.7	129.8, 164.0
7.71	dd (3; 5)	7.83	133.9	123.2, 132.1, 168.4
7.80	d (9)	6.96	131.9	114.7, 164.0, 190.8
7.83	dd (3; 5)	7.71	123.2	132.1, 133.9, 168.4
9.86	s		190.8	131.9
δ_C [ppm] from ^{13}C NMR: 190.8, 168.4, 164.0, 133.9, 132.1, 131.9, 129.8, 123.2, 114.7, 67.9, 37.7, 28.5, 28.2, 23.2.				

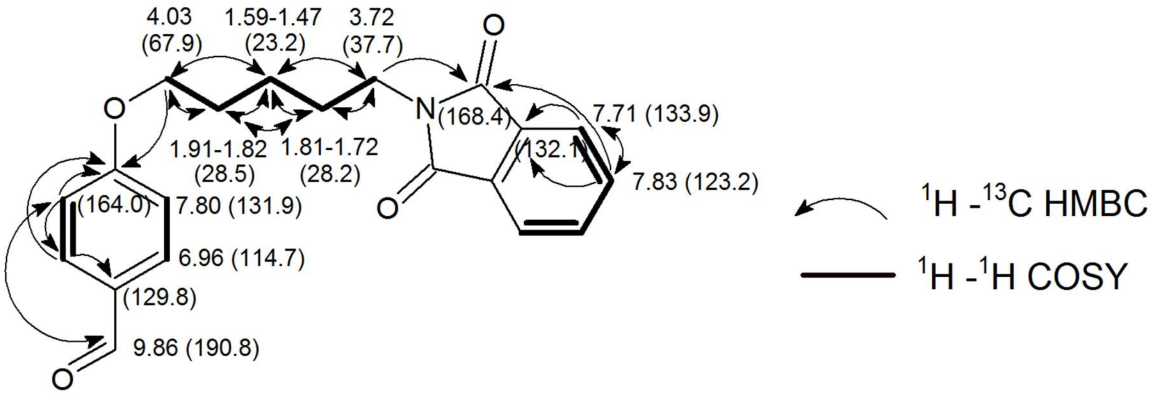

Fig. S1. ^{1}H and (^{13}C) chemical shift values [ppm] and key correlations observed in NMR spectra. Bold lines: ^{1}H-^{1}H COSY, Arrows: ^{1}H-^{13}C HMBC.

4-[8-(1,3-Dihydro-1,3-dioxo-2*H*-isoindol-2-yl)octyloxy]benzaldehyde (**1b**)

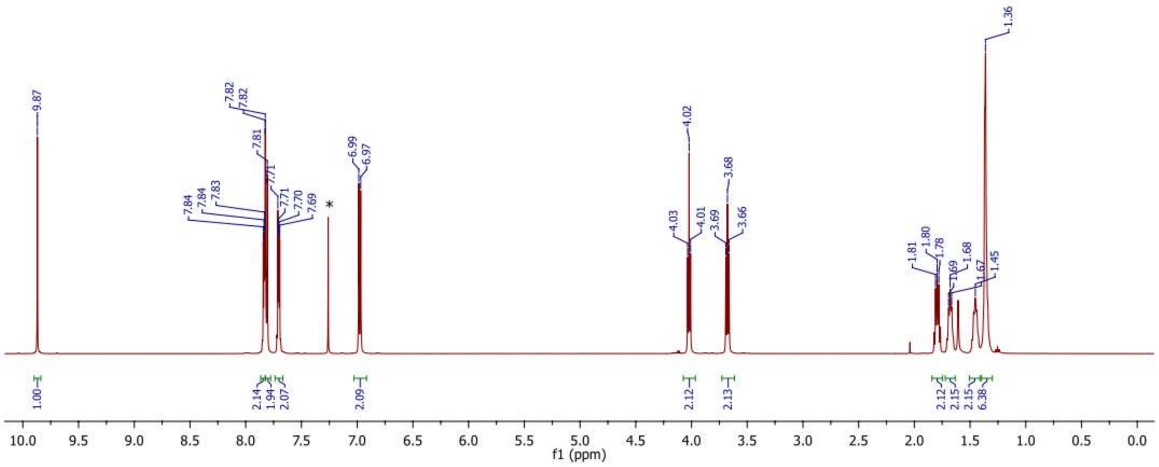

^{1}H NMR spectrum of **1b** (500 MHz, $CDCl_3$). The symbol * indicates chloroform residual peak.

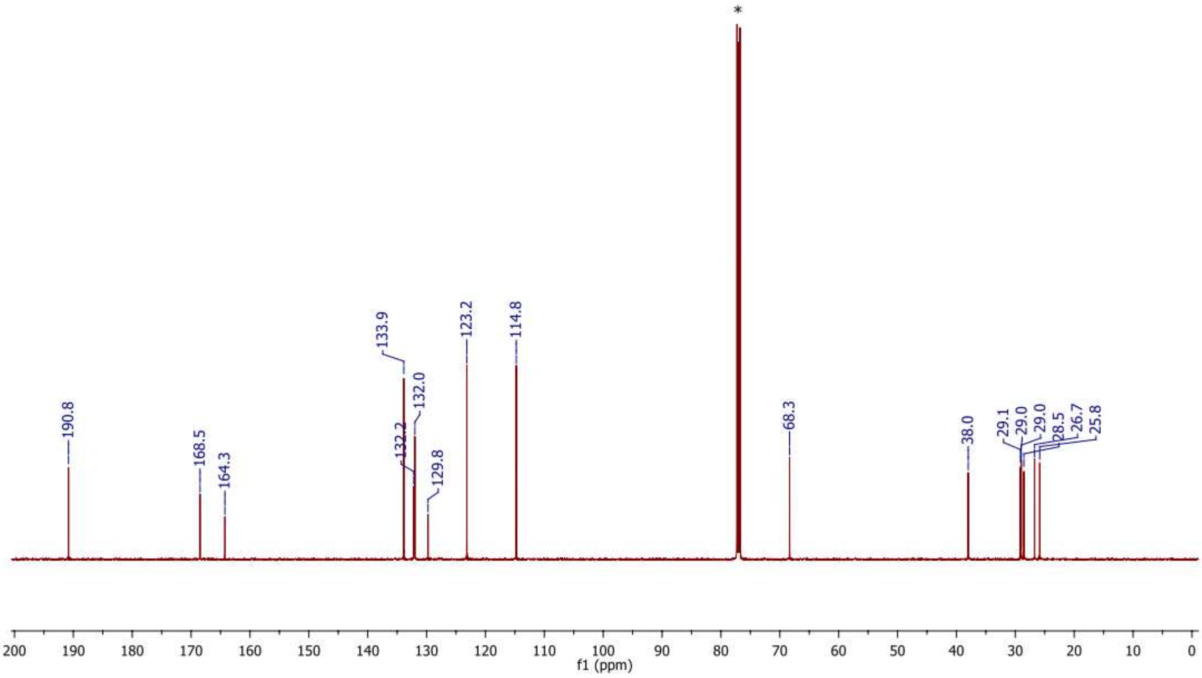

^{13}C NMR spectrum of **1b** (126 MHz, $CDCl_3$). The symbol * indicates chloroform residual peak.

Table S2. ^{1}H and ^{13}C NMR data obtained for **1b** including key correlations determined from ^{1}H-^{1}H COSY, ^{1}H-^{13}C HSQC and ^{1}H-^{13}C HMBC spectra.

δ_H [ppm]	Multiplicity (J_{H-H} w Hz)	^{1}H-^{1}H COSY δ_H [ppm]	^{1}H-^{13}C HSQC δ_C [ppm]	^{1}H-^{13}C HMBC δ_C [ppm]
1.36	s	1.49-1.41, 1.72-1.65, 1.84-1.75,	26.7, 29.1	25.8, 28.5, 29.0, 38.0
1.49-1.41	m	1.36, 1.84-1.75	25.8	29.0, 68.3
1.72-1.65	m	1.36, 3.68	28.5	26.7, 38.0
1.84-1.75	m	1.36, 1.49-1.41, 4.02	29.0	25.8, 29.0, 68.3
3.68	t (7)	1.72-1.65	38.0	26.7, 28.5, 168.5,
4.02	t (7)	1.84-1.75	68.3	25.8, 29.0, 164.3,
6.98	d (9)	7.81	114.8	129.8, 164.3
7.70	dd (3; 5)	7.83	133.9	123.2, 132.2, 168.5
7.81	d (9)	6.98	132.0	114.8, 164.3, 190.8
7.83	dd (3; 5)	7.70	123.2	133.9, 168.4
9.87	s		190.8	132.0
δ_C [ppm] from ^{13}C NMR: 190.8, 168.5, 164.3, 133.9, 132.2, 132.0, 130.0, 129.8, 123.2, 114.8, 68.3, 38.0, 29.1, 29.0, 28.5, 26.7, 25.8				

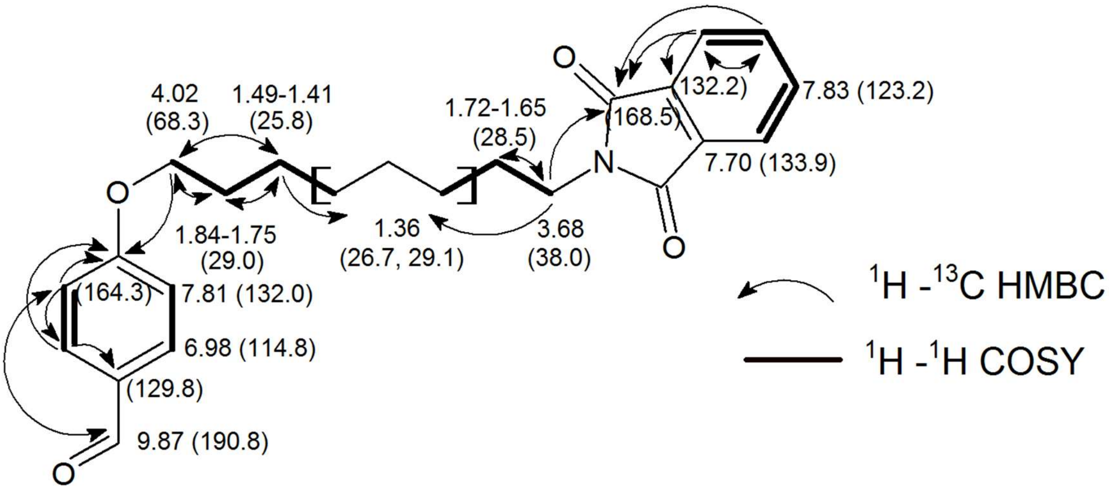

Fig. S2. ^{1}H and (^{13}C) chemical shift values [ppm] and key correlations observed in NMR spectra. Bold lines: ^{1}H-^{1}H COSY, Arrows: ^{1}H-^{13}C HMBC.

1,3,5,7-Tetramethyl-8-{4-[5-(phthalimidyl)pentyloxy]phenyl}-4,4-difluoro-4-bora-3a,4a-diaza-s-indacene (**2a**)

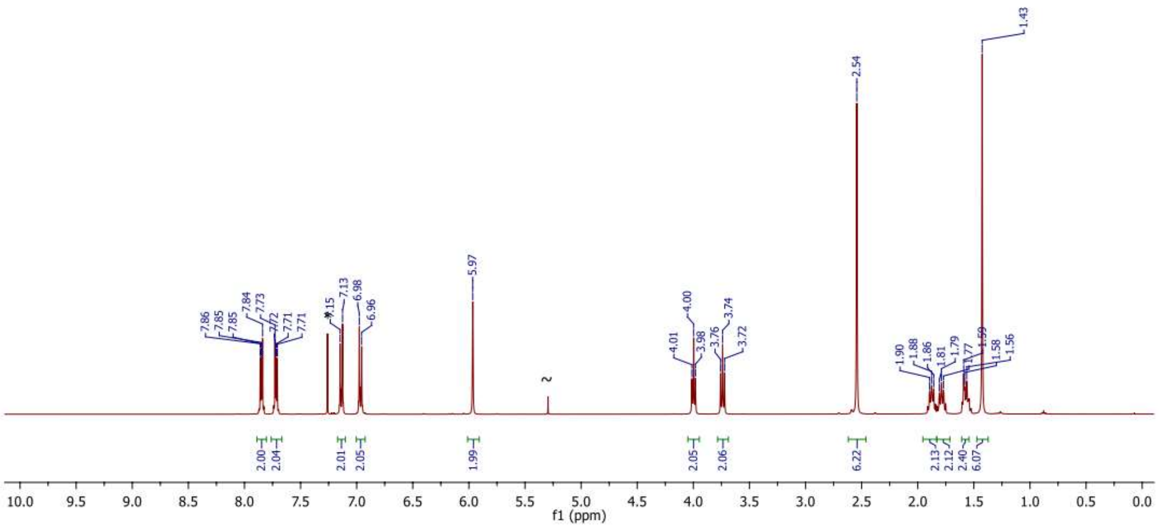

^{1}H NMR spectrum of **2a** (400 MHz, $CDCl_3$). The symbols * and ~ indicate residual peaks of chloroform and dichloromethane, respectively.

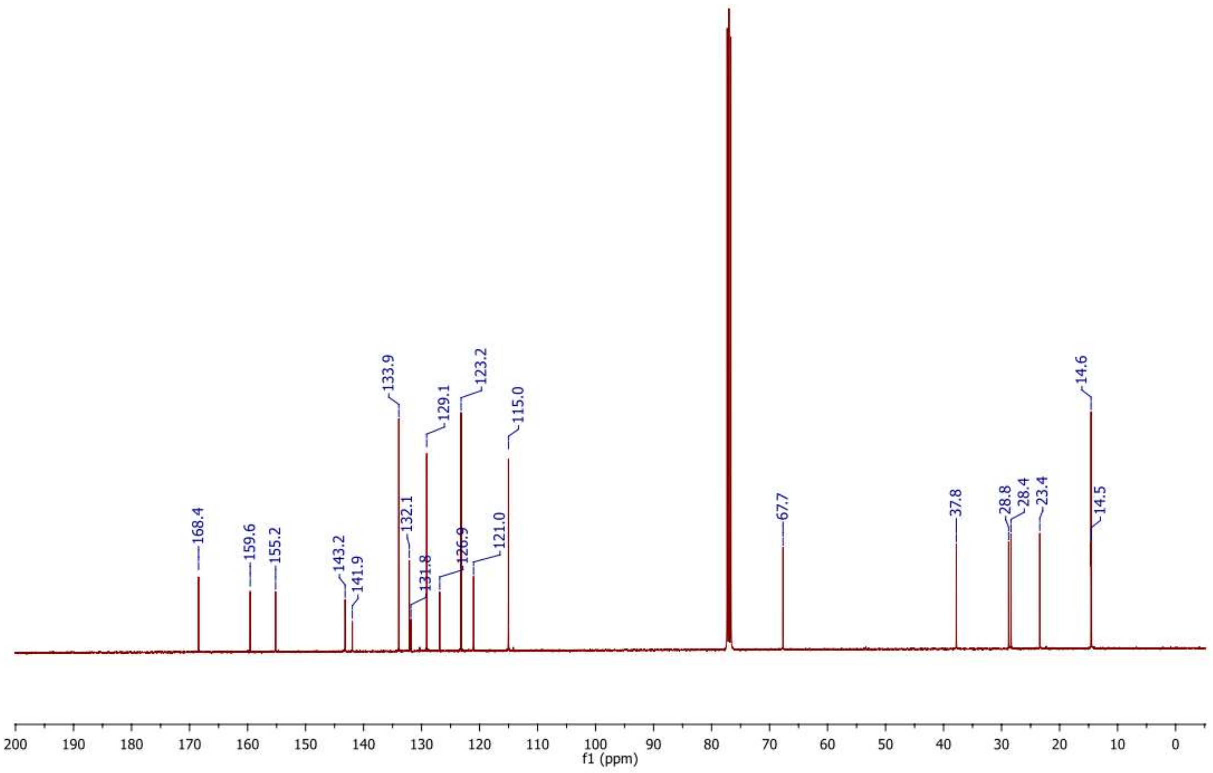

^{13}C NMR spectrum of **2a** (100 MHz, $CDCl_3$).

Table S3. ^{1}H and ^{13}C NMR data obtained for **2a** including key correlations determined from ^{1}H-^{1}H COSY, ^{1}H-^{13}C HSQC and ^{1}H-^{13}C HMBC spectra.

δ_H [ppm]	Multiplicity (J_{H-H} w Hz)	^{1}H-^{1}H COSY δ_H [ppm]	^{1}H-^{13}C HSQC δ_C [ppm]	^{1}H-^{13}C HMBC δ_C [ppm]
1.43	s	2.54, 5.97	14.6/14.5	121.0, 131.8, 143.2, 155.2
1.61-1.52	m	1.83-1.74, 1.92-1.84	23.4	28.4, 28.8, 37.8, 67.7
1.83-1.74	m	1.61-1.52, 3.74	28.4	23.4, 28.8, 37.8
1.92-1.84	m	1.61-1.52, 4.00	28.8	23.4, 28.4, 67.7
2.54	s	1.43, 5.97	14.6/14.5	121.0, 143.2, 155.2
3.74	t (7)	1.83-1.74	37.8	23.4, 28.4, 168.4
4.00	t (6)	1.92-1.84	67.7	23.4, 28.8, 159.6
5.97	s	1.43, 2.54	121.0	14.6/14.5, 131.8, 143.2, 155.2,
6.97	d (9)	7.14	115.0	126.9, 129.1, 159.6
7.14	d (9)	6.97	129.1	115.0, 141.9, 159.6
7.72	dd (3, 5)	7.85	133.9	123.2, 132.1, 168.4
7.85	dd (3, 5)	7.72	123.2	132.1, 133.9, 168.4
δ_C [ppm] from ^{13}C NMR: 168.4, 159.6, 155.2, 143.2, 141.9, 133.9, 132.1, 131.8, 129.1, 126.9, 123.2, 121.0, 115.0, 67.7, 37.8, 28.8, 28.4, 23.4, 14.6, 14.5.				

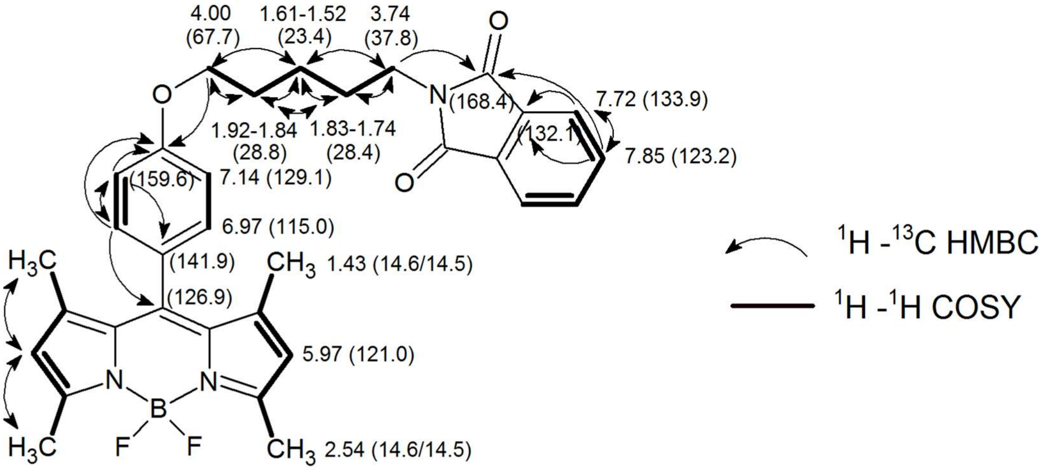

Fig. S3. ^{1}H and (^{13}C) chemical shift values [ppm] and key correlations observed in NMR spectra. Bold lines: ^{1}H-^{1}H COSY, Arrows: ^{1}H-^{13}C HMBC.

1,3,5,7-Tetramethyl-8-{4-[8-(phthalimidyl)octyloxy]phenyl}-4,4-difluoro-4-bora-3a,4a-diaza-s-indacene (**2b**)

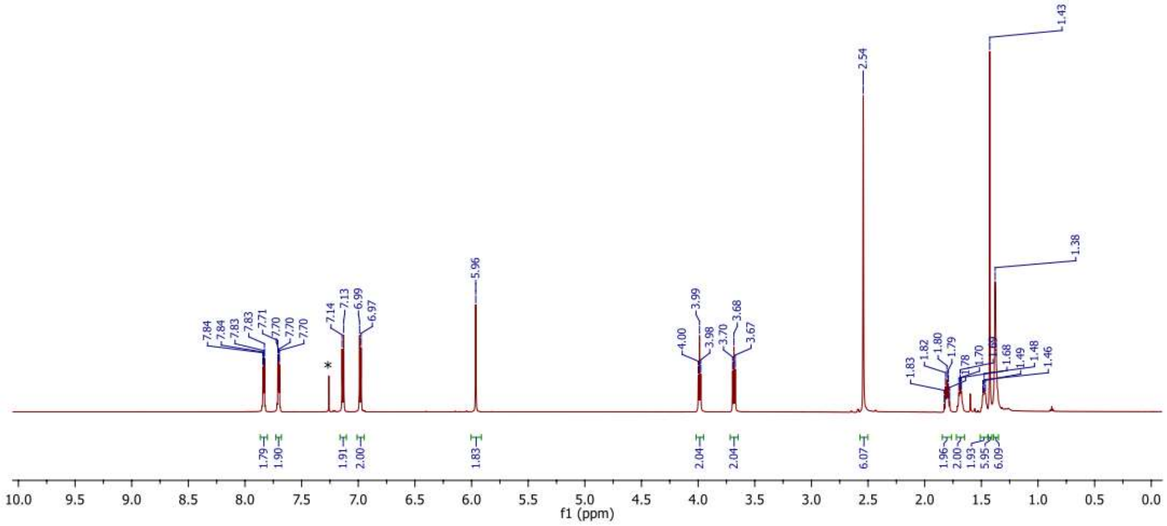

^{1}H NMR spectrum of **2b** (600 MHz, $CDCl_3$). The symbol * indicates a chloroform residual peak.

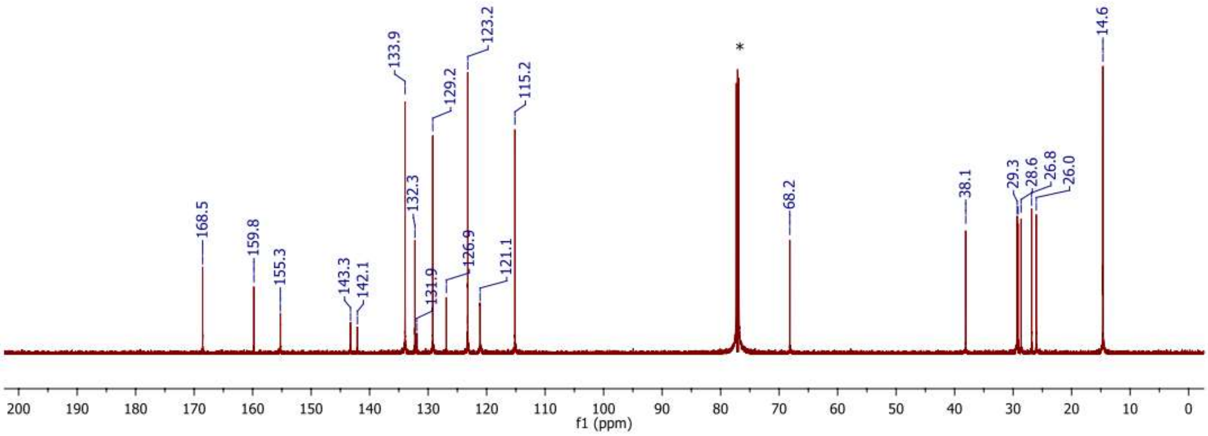

^{13}C NMR spectrum of **2b** (151 MHz, $CDCl_3$). The symbol * indicates a chloroform residual peak.

Table S4. ^{1}H and ^{13}C NMR data obtained for **2b** including key correlations determined from ^{1}H-^{1}H COSY, ^{1}H-^{13}C HSQC and ^{1}H-^{13}C HMBC spectra.

δ_H [ppm]	Multiplicity (J_{H-H} w Hz)	^{1}H-^{1}H COSY δ_H [ppm]	^{1}H-^{13}C HSQC δ_C [ppm]	^{1}H-^{13}C HMBC δ_C [ppm]
1.38	s	1.72-1.65, 1.83-1.74	26.8, 29.1	26.0, 38.1, 29.1
1.43	s	2.54, 5.96	14.6	121.1, 131.9, 143.3, 155.3
1.51-1.44	m	1.83-1.77	26.0	26.8, 29.1, 68.2
1.72-1.65	m	1.38, 3.68	28.6	26.8, 29.1, 38.1
1.83-1.77	m	1.51-1.44, 3.99	29.1	26.0, 29.3, 68.2
2.54	s	1.43	14.6	121.1, 131.9, 142.1, 143.3, 155.3
3.68	t (7)	1.72-1.65	38.1	26.8, 28.6, 168.5
3.99	t (7)	1.83-1.77	68.2	26.0, 29.1, 159.8
5.96	s	1.43	121.1	14.6, 131.9, 143.3, 155.3,
6.98	d (8.5)	7.14	115.2	126.9, 129.2, 159.8
7.14	d (9)	6.98	129.2	115.2, 142.1, 159.8
7.70	dd (3, 5)	7.84	133.9	123.2, 132.3, 168.5
7.84	dd (3, 5)	7.70	123.2	132.3, 133.9, 168.5
δ_C [ppm] from ^{13}C NMR: 168.5, 159.8, 155.3, 143.3, 142.1, 133.9, 132.3, 131.9, 129.2, 126.9, 123.2, 121.1, 115.2, 68.2, 38.1, 29.3, 29.1, 28.6, 26.8, 26.0, 14.6.				

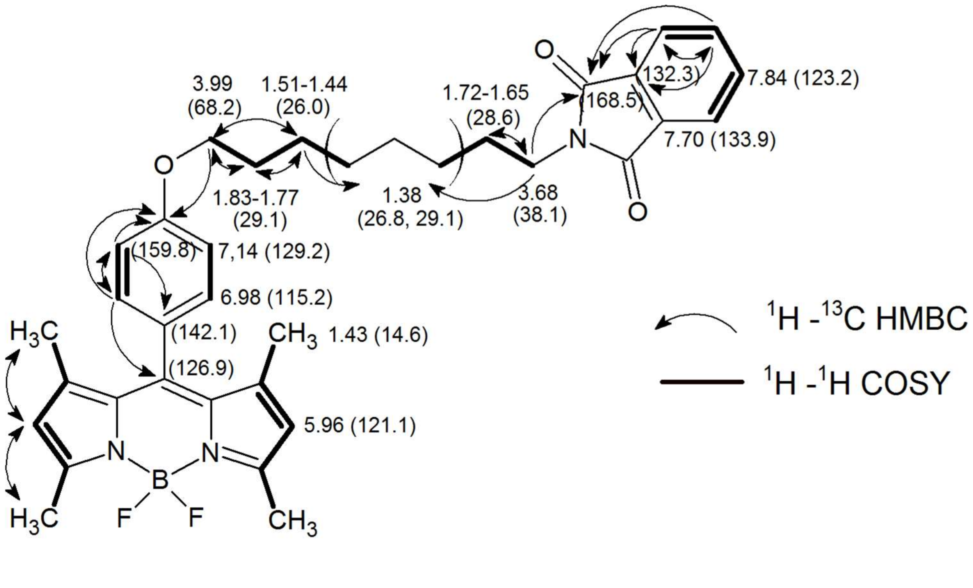

Fig. S4. ^{1}H and (^{13}C) chemical shift values [ppm] and key correlations observed in NMR spectra. Bold lines: ^{1}H-^{1}H COSY, Arrows: ^{1}H-^{13}C HMBC.

2,6-Diiodo-1,3,5,7-tetramethyl-8-{4-[5-(phthalimidyl)pentyloxy]phenyl}-4,4-difluoro-4-bora-3a,4a-diaza-s-indacene (**3a**)

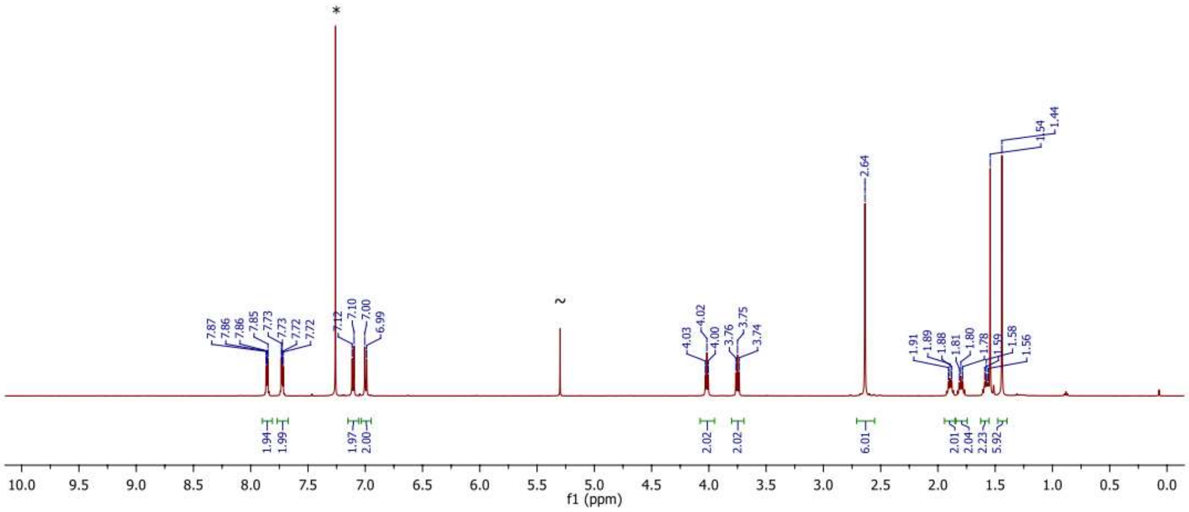

^{1}H NMR spectrum of **3a** (500 MHz, $CDCl_3$). The symbol * and ~ indicate residual peaks of chloroform and dichloromethane, respectively.

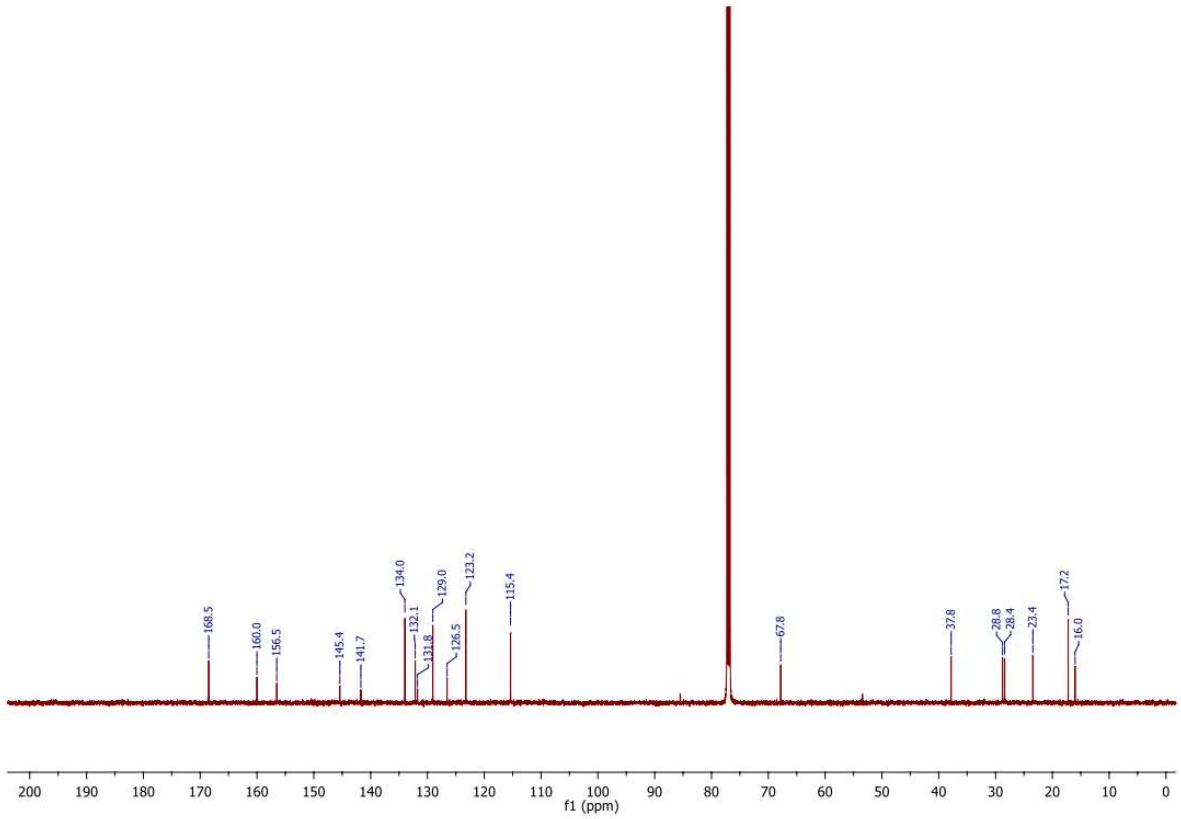

^{13}C NMR spectrum of **3a** (126 MHz, $CDCl_3$).

Table S5. ^{1}H and ^{13}C NMR data obtained for **3a** including key correlations determined from ^{1}H-^{1}H COSY, ^{1}H-^{13}C HSQC and ^{1}H-^{13}C HMBC spectra.

δ_H [ppm]	Multiplicity ($J_{H\text{-}H}$ w Hz)	^{1}H-^{1}H COSY δ_H [ppm]	^{1}H-^{13}C HSQC δ_C [ppm]	^{1}H-^{13}C HMBC δ_C [ppm]
1.44	s	2.64	17.2	131.8, 145.4, 156.5
1.62-1.57	m	1.84-1.76, 1.93-1.86	23.4	28.4, 28.8, 37.8, 67.8
1.84-1.76	m	1.62-1.57, 3.75	28.4	23.4, 28.8, 37.8
1.93-1.86	m	1.62-1.57, 4.02	28.8	23.4, 28.4, 67.8
2.64	s	1.44	16.0	145.4, 156.5
3.75	t (7)	1.84-1.76	37.8	23.4, 28.4, 168.5
4.02	t (6)	1.93-1.86	67.8	23.4, 28.8, 160.0
7.00	d (9)	7.11	115.4	126.5, 160.0
7.11	d (9)	7.00	129.0	141.7, 160.0
7.73	dd (3, 5)	7.86	134.0	123.2, 132.1
7.86	dd (3, 5)	7.73	123.2	132.1, 134.0, 168.5
δ_C [ppm] from ^{13}C NMR: 168.5, 160.0, 156.5, 145.4, 141.7, 134.0, 132.1,131.8, 129.0, 126.5, 123.2, 115.4, 67.8, 37.8, 28.8, 28.4, 23.4, 17.2, 16.0.				

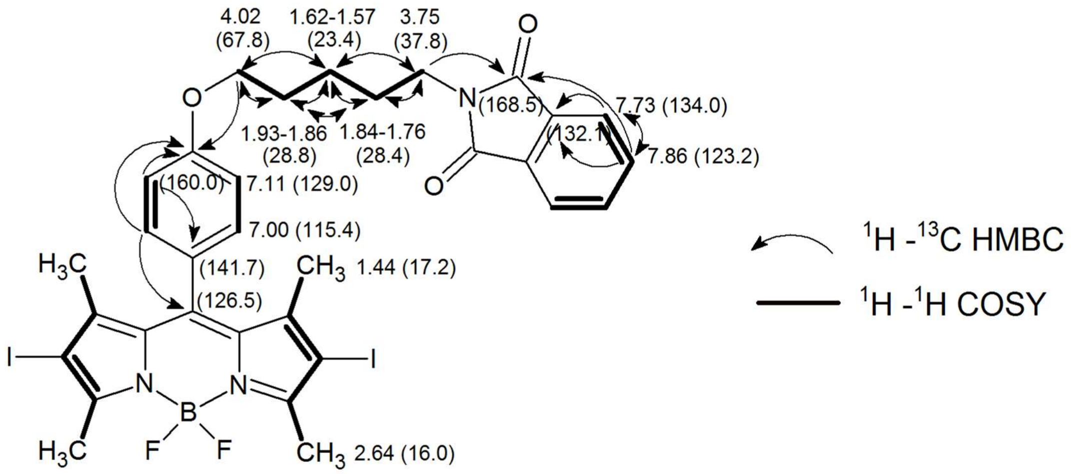

Fig. S5. ^{1}H and (^{13}C) chemical shift values [ppm] and key correlations observed in NMR spectra. Bold lines: ^{1}H-^{1}H COSY. Arrows: ^{1}H-^{13}C HMBC.

2,6-Diiodo-1,3,5,7-tetramethyl-8-{4-[8-(phthalimidyl)octyloxy]phenyl}-4,4-difluoro-4-bora-3a,4a-diaza-s-indacene (**3b**)

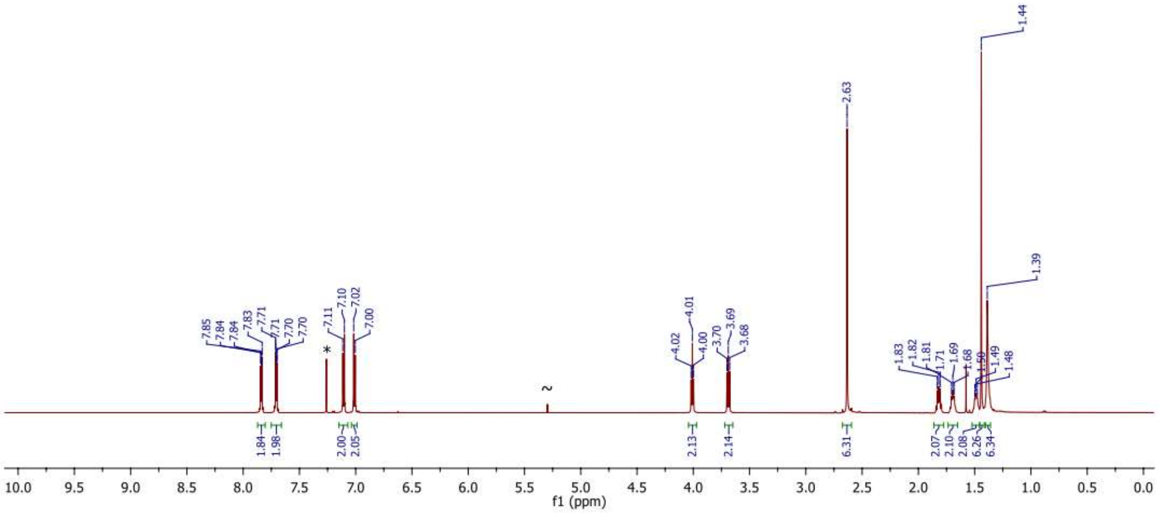

^{1}H NMR spectrum of **3b** (600 MHz, $CDCl_3$). The symbol * and ~ indicate residual peaks of chloroform and dichloromethane, respectively.

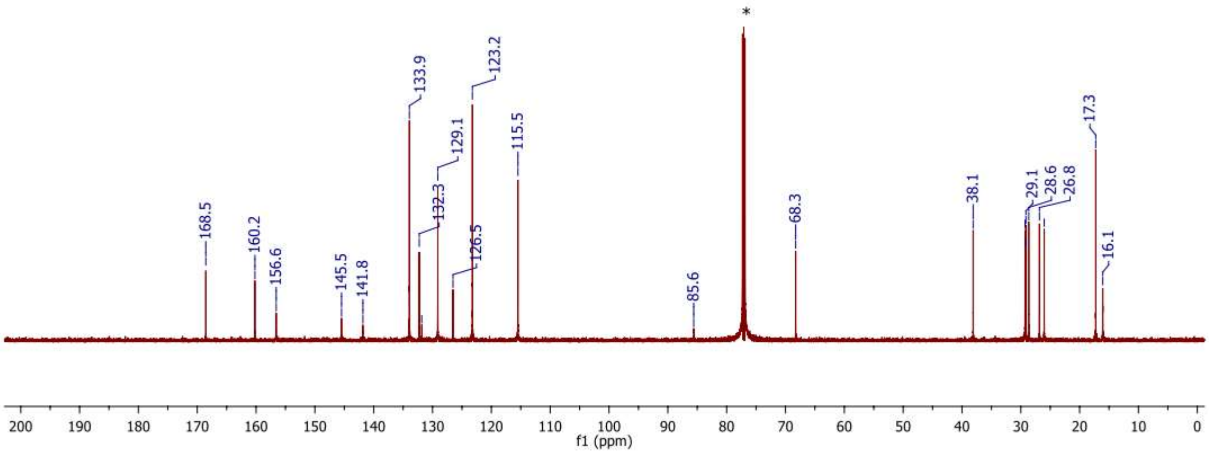

^{13}C NMR spectrum of **3b** (151 MHz, $CDCl_3$). The symbol * indicates a chloroform residual peak.

Table S6. ^{1}H and ^{13}C NMR data obtained for **3b** including key correlations determined from ^{1}H-^{1}H COSY, ^{1}H-^{13}C HSQC and ^{1}H-^{13}C HMBC spectra.

δ_H [ppm]	Multiplicity (J_{H-H} w Hz)	^{1}H-^{1}H COSY δ_H [ppm]	^{1}H-^{13}C HSQC δ_C [ppm]	^{1}H-^{13}C HMBC δ_C [ppm]
1.39	s	1.72-1.66	26.8, 29.2/29.3	26.0, 28.6, 29.1, 38.1
1.44	s	2.63	17.3	16.1, 85.6, 131.8, 141.8, 145.5 156.6
1.52-1.44	m	1.84-1.79	26.0	26.8, 29.1, 68.3
1.72-1.66	m	1.39, 3.69	28.6	26.8, 29.2/29.3, 38.1
1.84-1.79	m	1.52-1.44, 4.01	29.1	26.0, 68.3
2.63	s	1.44	16.1	17.3, 85,6, 131.8 145.5, 156.5
3.69	t (7)	1.72-1.66	38.1	26.8, 28.6, 168.5
4.01	t (7)	1.84-1.79	68.3	26.0, 29.1, 160,2
7.01	d (9)	7.11	115.5	126.5, 129.1, 160,2
7.11	d (9)	7.01	129.1	115.5, 141.8, 160,2
7.71	dd (3, 5)	7.84	133.9	123.2, 132.3, 168.5
7.84	dd (3, 5)	7.71	123.2	132.3, 133.9, 168.5
δ_C [ppm] from ^{13}C NMR: 168.5, 160.2, 156.6, 145.5, 141.8, 133.9, 132.3, 131.8, 129.1, 126.5, 123.2, 115.5, 85.6, 68.3, 38.1, 29.3, 29.2, 29.1, 28.6, 26.8, 26.0, 17.3, 16.1.				

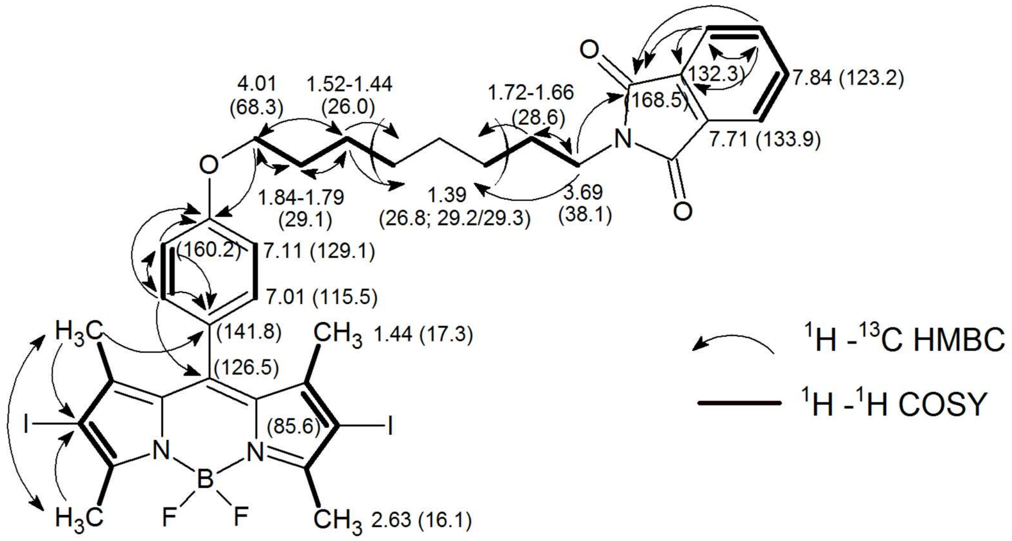

Fig. S6. ^{1}H and (^{13}C) chemical shift values [ppm] and key correlations observed in NMR spectra. Bold lines: ^{1}H-^{1}H COSY, Arrows: ^{1}H-^{13}C HMBC.

2,6-Dibromo-1,3,5,7-tetramethyl-8-{4-[5-(phthalimidyl)pentyloxy]phenyl}-4,4-difluoro-4-bora-3a,4a-diaza-s-indacene (**4a**)

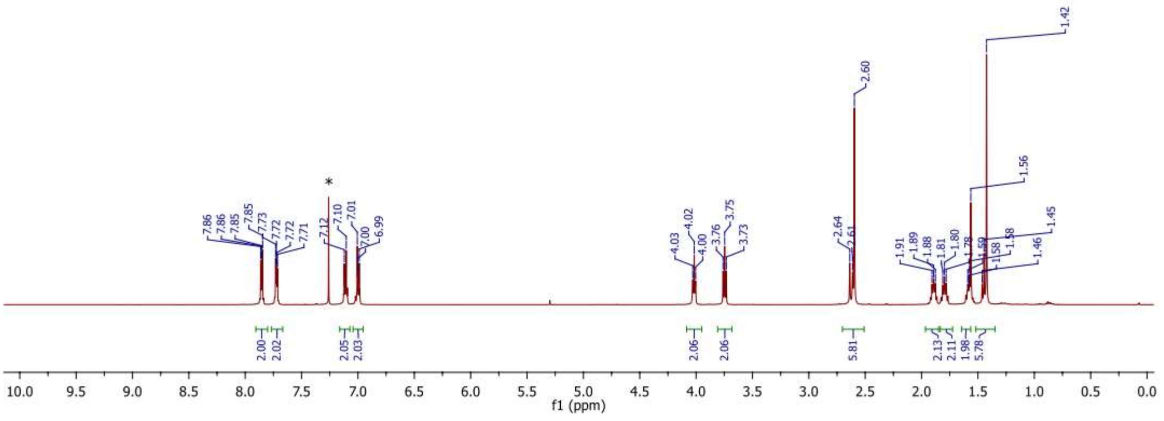

^{1}H NMR spectrum of **4a** (500 MHz, $CDCl_3$). The symbol * indicates a chloroform residual peak.

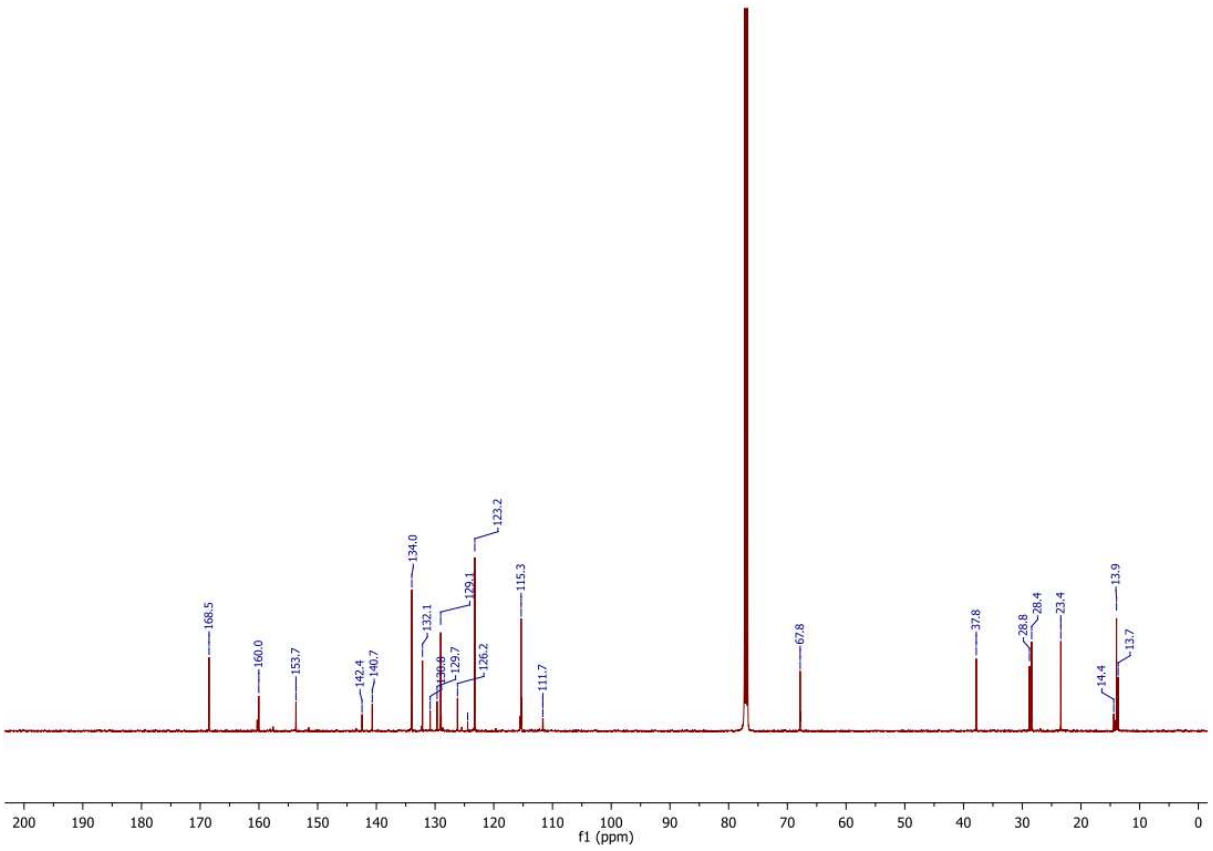

^{13}C NMR spectrum of **4a** (126 MHz, $CDCl_3$).

Table S7. ^{1}H and ^{13}C NMR data obtained for **4a** including key correlations determined from ^{1}H-^{1}H COSY, ^{1}H-^{13}C HSQC and ^{1}H-^{13}C HMBC spectra.

δ_H [ppm]	Multiplicity (J_{H-H} w Hz)	^{1}H-^{1}H COSY δ_H [ppm]	^{1}H-^{13}C HSQC δ_C [ppm]	^{1}H-^{13}C HMBC δ_C [ppm]
1.47-1.41	m	2.66-2.56	13.9	13.7, 111.7, 130.8, 140.7, 153.7
1.62-1.56	m	1.83-1.76, 1.93-1.86,	23.4	28.4, 37.8, 67.8
1.83-1.76	m	1.62-1.56, 3.75	28.4	23.4, 28.8. 37.8
1.93-1.86	m	1.62-1.56, 4.02	28.8	23.4, 28.4, 67.8
2.66-2.56	m	1.47-1.41	13.7	13.9, 111.7. 140.7, 142.4, 153.7
3.75	t (7)	1.86-1.73	37.8	23.4. 28.4, 168.5
4.02	t (6)	1.93-1.86	67.8	23.4, 28.8, 160.0
7.00	d (9)	7.11	115.3	124.4, 126.2. 160.0
7.11	d (9)	7.00	129.1	142.4.160.0
7.72	dd (3. 5)	7.85	134.0	123.2. 132.1, 168.5
7.85	dd (3. 5)	7.72	123.2	132.1, 134.0, 168.5
δ_C [ppm] from 13C NMR (126 MHz. CDCl3) δ 168.5, 160.0, 153.7, 142.4, 140.7, 134.0, 132.1, 130.8, 129.7, 129.1, 126.2, 124.4, 123.2, 115.3, 111.7, 67.8, 37.8, 28.8, 28.4, 23.4, 14.4, 13.9, 13.7				

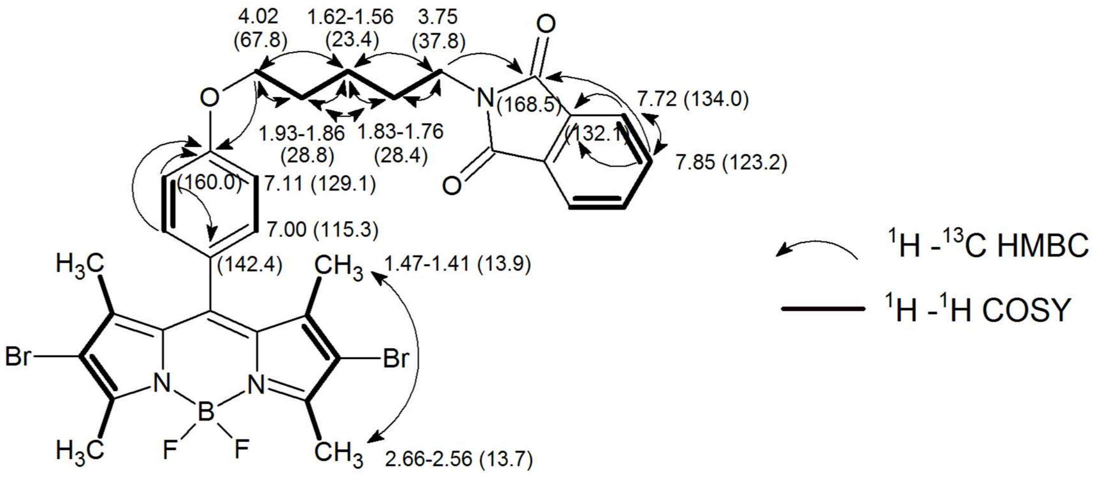

Fig. S7. ^{1}H and (^{13}C) chemical shift values [ppm] and key correlations observed in NMR spectra. Bold lines: ^{1}H-^{1}H COSY. Arrows: ^{1}H-^{13}C HMBC.

2,6-Dibromo-1,3,5,7-tetramethyl-8-{4-[8-(phthalimidyl)octyloxy]phenyl}-4,4-difluoro-4-bora-3a,4a-diaza-s-indacene (**4b**)

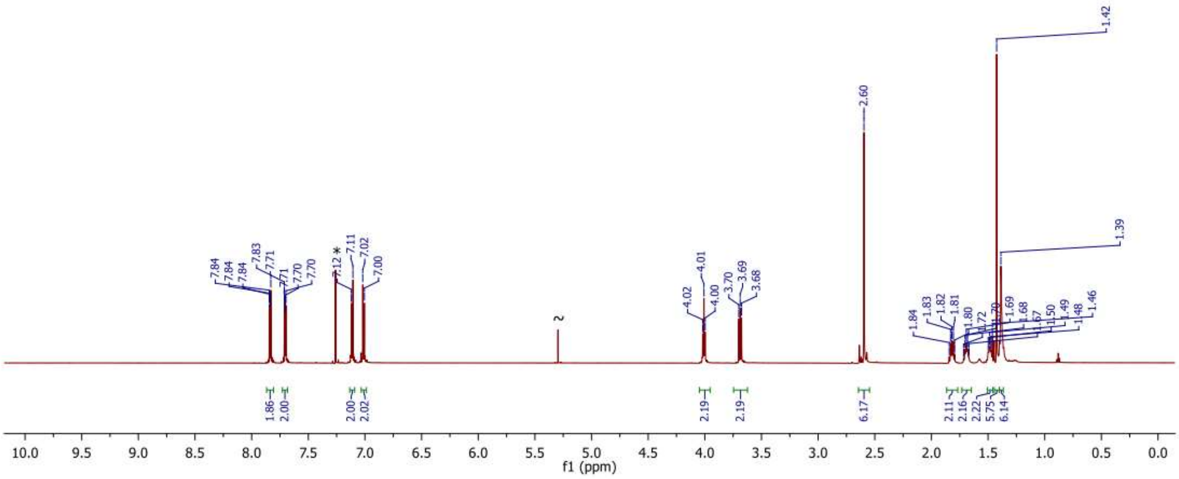

^{1}H NMR spectrum of **4b** (600 MHz, $CDCl_3$). The symbol * and ~ indicate residual peaks of chloroform and dichloromethane, respectively.

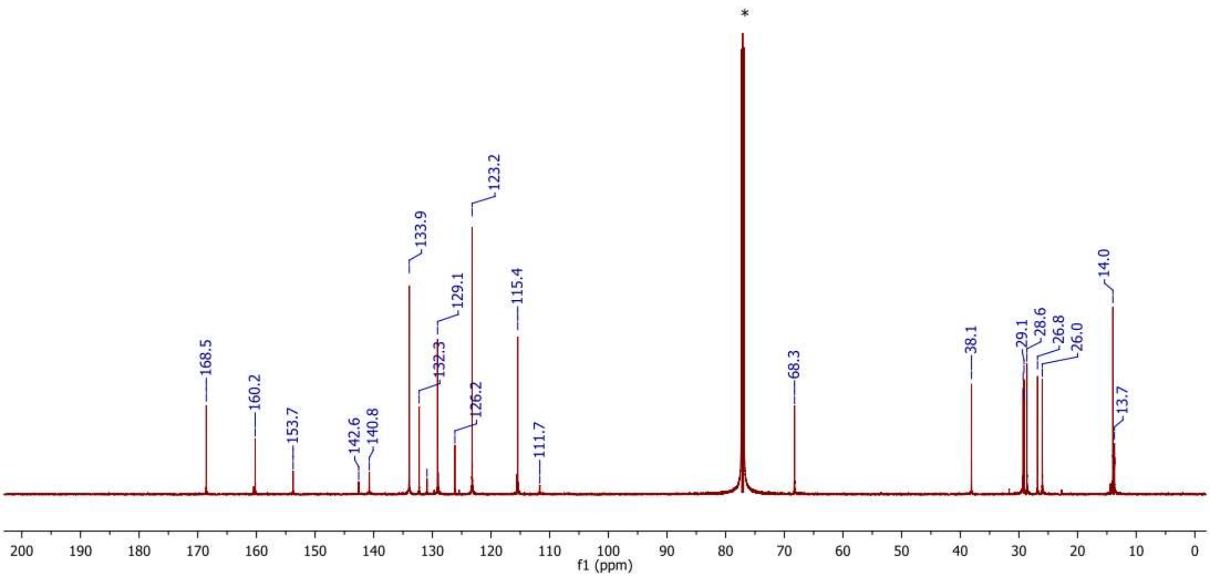

^{13}C NMR spectrum of **4b** (151 MHz, $CDCl_3$). The symbol * indicates a chloroform residual peak.

Table S8. ^{1}H and ^{13}C NMR data obtained for **4b** including key correlations determined from ^{1}H-^{1}H COSY, ^{1}H-^{13}C HSQC and ^{1}H-^{13}C HMBC spectra.

δ_H [ppm]	Multiplicity (J_{H-H} w Hz)	^{1}H-^{1}H COSY δ_H [ppm]	^{1}H-^{13}C HSQC δ_C [ppm]	^{1}H-^{13}C HMBC δ_C [ppm]
1.39	s	1.51-1.46, 1.72-1.66	26.8, 29.2/29.3	26.0, 28.6, 29.1, 38.1
1.42	s	2.60	14.0	111.7, 130.9, 140.8, 153.7
1.51-1.46	m	1.39, 1.85-1.79	26.0	26.8, 29.1, 29.2/29.3, 68.3
1.72-1.66	m	1.39. 3.69	28.6	26.8, 29.2/29.3, 38.1
1.85-1.79	m	1.51-1.46. 4.01	29.1	26.0, 29.2/29.3, 68.3
2.60	s	1.42	13.7	111.7, 130.9, 140.8, 153.7
3.69	t (7)	1.72-1.66	38.1	26.8, 28.6, 168.5
4.01	t (7)	1.85-1.79	68.3	26.0, 29.1, 160.2
7.01	d (9)	7.11	115.4	126.2, 129.1, 142.6, 160.2
7.11	d (9)	7.01	129.1	115.4, 142.6. 160.2
7.70	dd (3. 5)	7.84	133.9	123.2, 132.3, 133.9
7.84	dd (3. 5)	7.70	123.2	132.3, 133.9, 168.5
δC [ppm] from 13C NMR: 168.5; 160.2; 153.7; 142.6; 140.8; 133.9; 132.3; 130.9; 129.1; 126.2; 123.2; 115.4; 111.7; 68.3; 38.1; 29.3; 29.2; 29.1; 28.6; 26.8; 26.0; 14.0; 13.7.				

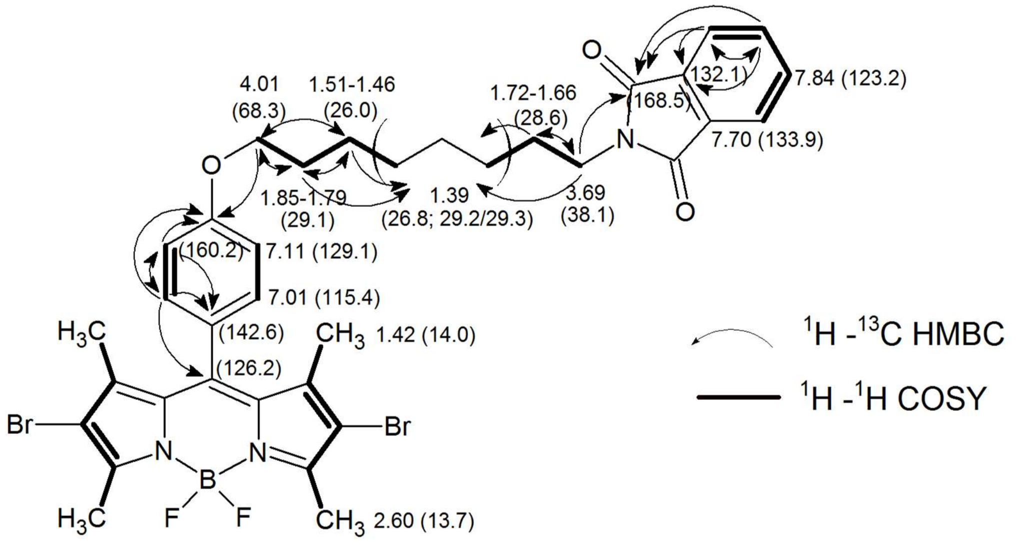

Fig. S8. ^{1}H and (^{13}C) chemical shift values [ppm] and key correlations observed in NMR spectra. Bold lines: ^{1}H-^{1}H COSY, Arrows: ^{1}H-^{13}C HMBC.

1,3,5,7-Tetramethyl-8-[4-(5-aminopentyloxy)phenyl]-4,4-difluoro-4-bora-3a,4a-diaza-s-indacene (**5a**)

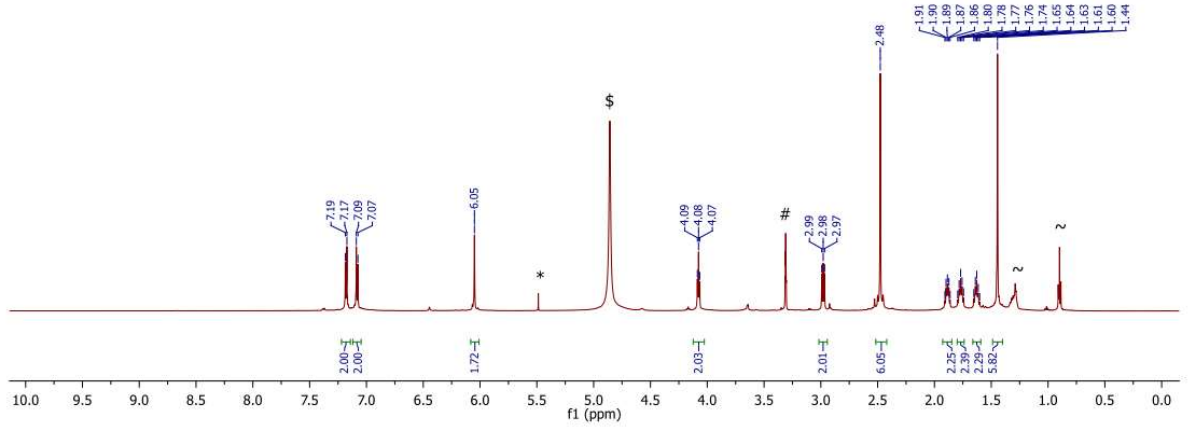

^{1}H NMR spectrum of **5a** (600 MHz, CD_3OD). The symbols *, $, # and ~ indicate residual peaks of dichloromethane, water, methanol, and hexane, respectively.

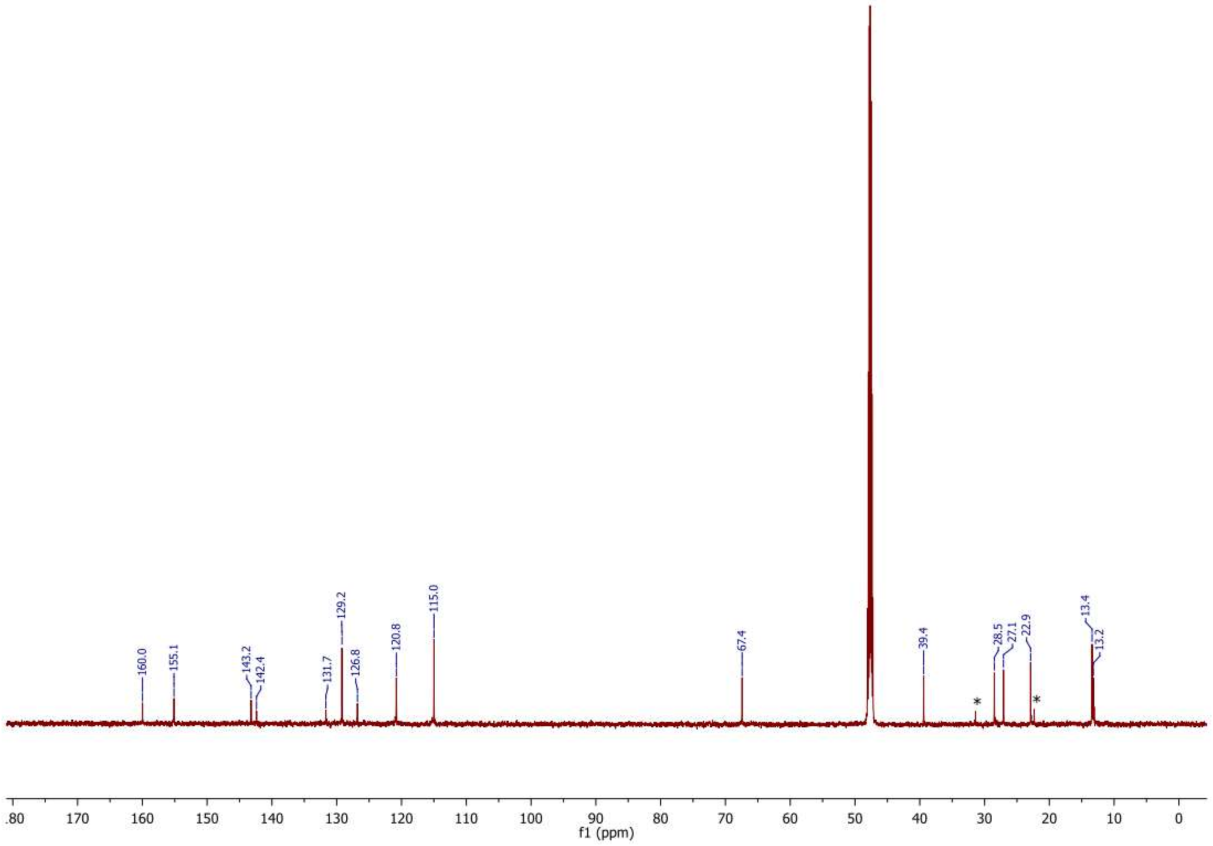

^{13}C NMR spectrum of **5a** (151 MHz, MeOD). The symbol * indicates hexane residual peaks.

Table S9. ^{1}H and ^{13}C NMR data obtained for **5a** including key correlations determined from ^{1}H-^{1}H COSY, ^{1}H-^{13}C HSQC and ^{1}H-^{13}C HMBC spectra.

δ_H [ppm]	Multiplicity (J_{H-H} w Hz)	^{1}H-^{1}H COSY δ_H [ppm]	^{1}H-^{13}C HSQC δ_C [ppm]	^{1}H-^{13}C HMBC δ_C [ppm]
1.44	s	2.48, 6.05	13.4	120.8, 131.7, 143.2, 155.1
1.66-1.60	m	1.80-1.74, 1.91-1.86	22.9	28.5, 39.4, 67.4
1.80-1.74	m	1.66-1.60, 3.01-2.95.	27.1	22.9, 28.5, 39.4
1.91-1.86	m	1.66-1.60, 4.08	28.5	22.9, 27.1, 67.4
2.48	s	1.44, 6.05	13.2	120.8, 143.2, 155.1
3.01-2.95	m	1.80-1.74	39.4	22.9, 27.1
4.08	t (6)	1.91-1.86	67.4	22.9, 28.5. 160.0
6.05	s	1.44, 2.48	120.8	13.2, 13.4, 131.7, 143.2, 155.1
7.08	d (9)	7.18	115.0	126.8, 160.0
7.18	d (9)	7.08	129.2	142.4, 160.0
δ_C [ppm] from ^{13}C NMR: 160.0, 155.1, 143.2, 142.4, 131.7, 129.2, 126.8, 120.8, 115.0, 67.4, 39.4, 28.5, 27.1, 22.9,13.4, 13.2.				

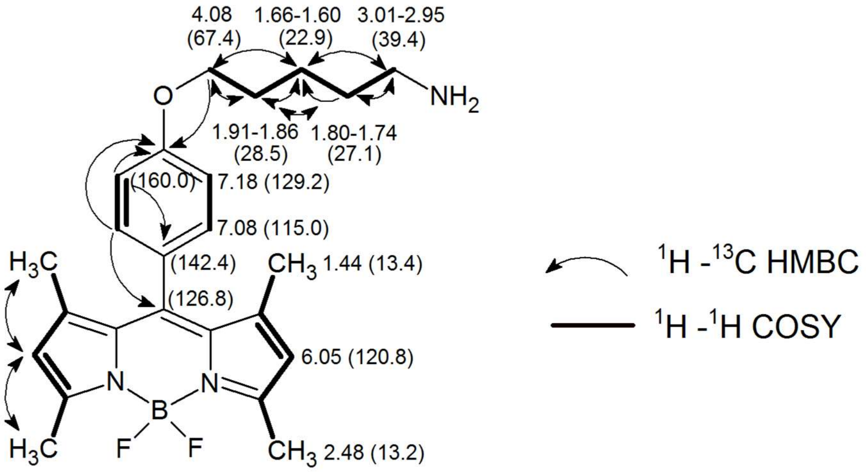

Fig. S9. ^{1}H and (^{13}C) chemical shift values [ppm] and key correlations observed in NMR spectra. Bold lines: ^{1}H-^{1}H COSY. Arrows: ^{1}H-^{13}C HMBC.

1,3,5,7-Tetramethyl-8-[4-(8-aminooctyloxy)phenyl]-4,4-difluoro-4-bora-3a,4a-diaza-s-indacene (**5b**)

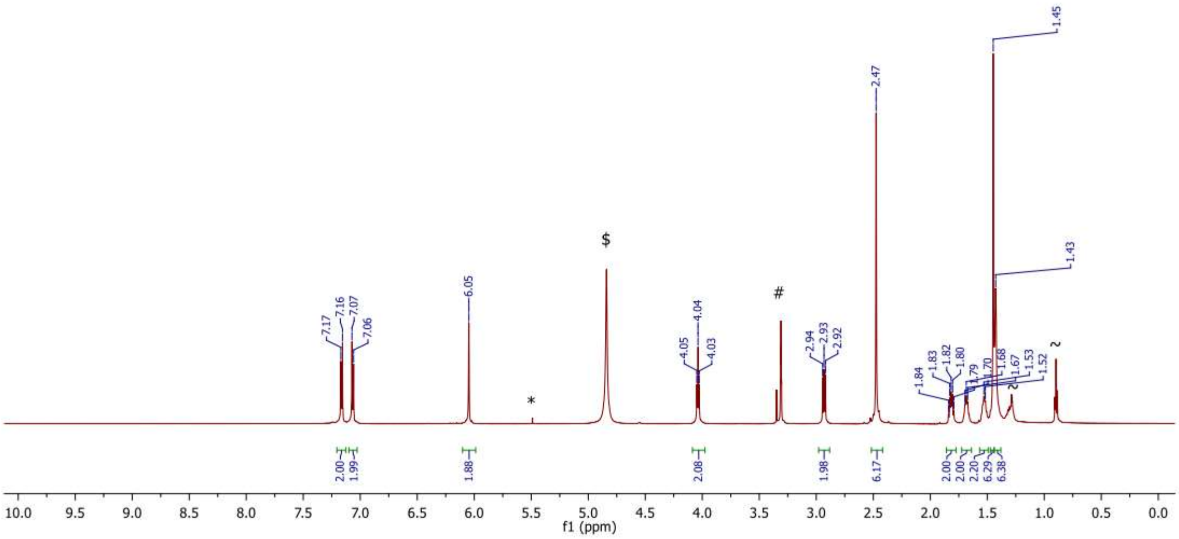

^{1}H NMR spectrum of **5b** (600 MHz, MeOD). The symbols *, $, # and ~ indicate residual peaks of dichloromethane, water, methanol, and hexane, respectively.

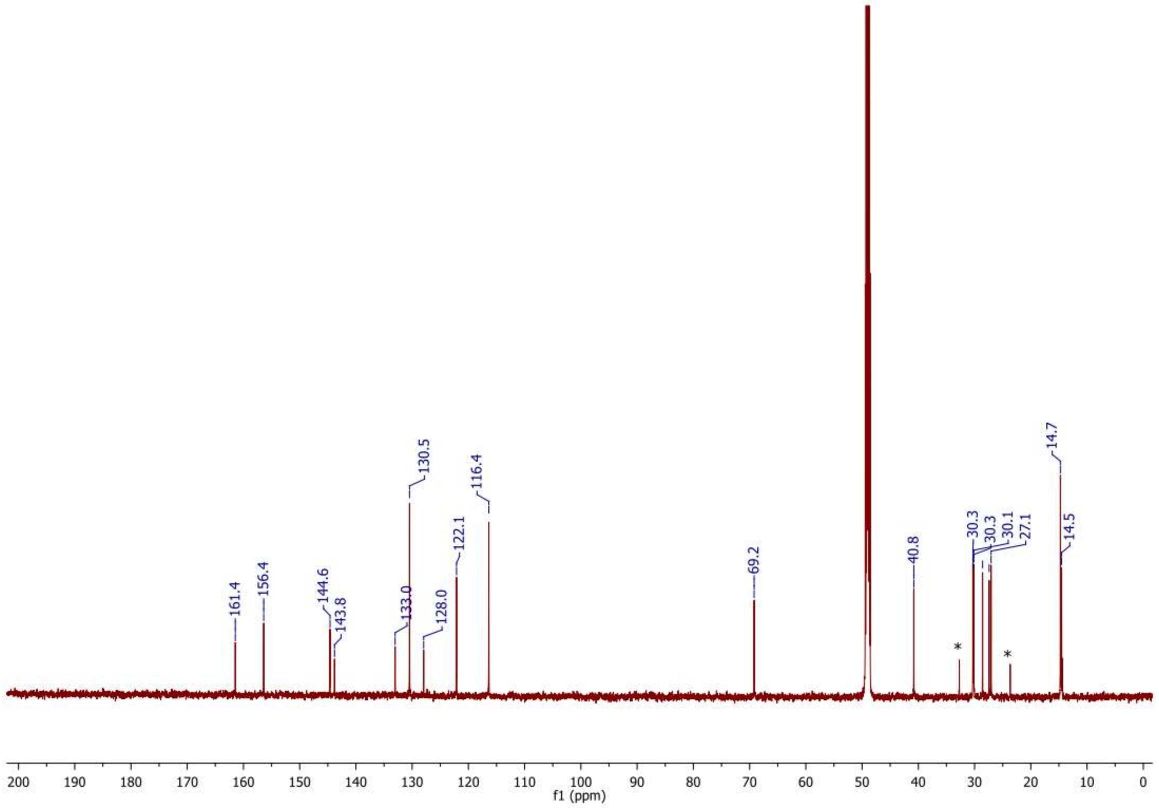

^{13}C NMR spectrum of **5b** (151 MHz, MeOD). The symbol * indicates hexane residual peaks.

Table S10. ^{1}H and ^{13}C NMR data obtained for **5b** including key correlations determined from ^{1}H-^{1}H COSY, ^{1}H-^{13}C HSQC and ^{1}H-^{13}C HMBC spectra.

δ_H [ppm]	Multiplicity ($J_{H\text{-}H}$ w Hz)	^{1}H-^{13}C HSQC δ_C [ppm]	^{1}H-^{13}C HMBC δ_C [ppm]
1.43	s	27.4, 30.1	27.1, 28.6
1.45	s	14.7	122.1, 133.0, 143.8, 144.6, 156.4
1.56-1.49	m	27.1	30.1, 30.3
1.71-1.66	m	28.6	27.4, 40.8
1.85-1.78	m	30.3	27.1, 69.2
2.47	s	14.5	122.1, 133.0, 143.8, 144.6, 156.4
2.93	d (8)	40.8	27.4, 28.6
4.04	t (6)	69.2	27.1, 30.3, 161.4
6.05	s	122.1	14.5, 133.0, 143.8,144.6,156.5
7.06	d (9)	116.4	128.0, 130.5, 144.6, 161.4,
7.16	d (9)	130.5	116.4, 144.6, 161.4,
δ_C [ppm] from ^{13}C NMR: 161.4, 156.4, 144.6, 143.8, 133.0, 130.5, 128.0, 122.1, 116.4, 69.2, 40.8, 30.3, 30.1, 28.6, 27.4, 27.1, 14.7, 14.5			

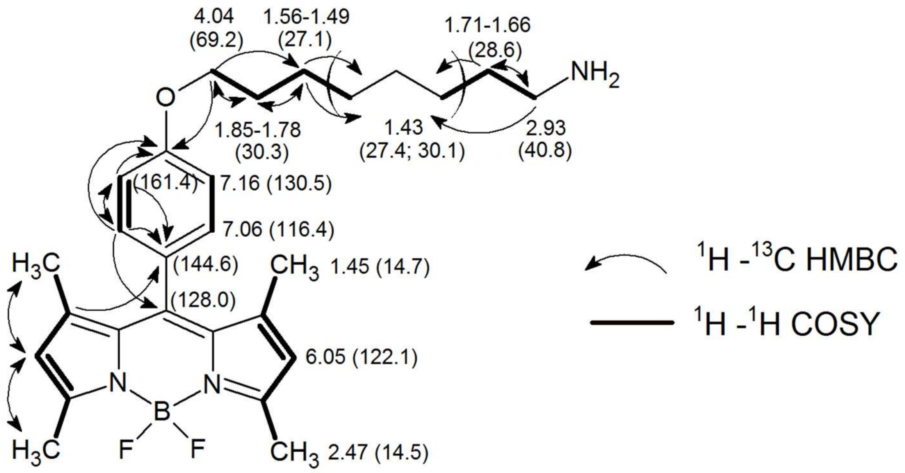

Fig.S10. ^{1}H and (^{13}C) chemical shift values [ppm] and key correlations observed in NMR spectra. Bold lines: ^{1}H-^{1}H COSY. Arrows: ^{1}H-^{13}C HMBC.

HRMS spectra

4-[5-(1,3-Dihydro-1,3-dioxo-2*H*-isoindol-2-yl)pentyloxy]benzaldehyde (**1a**)

Acquisition Parameter

Source Type	ESI	Ion Polarity	Positive	Set Nebulizer	0.4 Bar
Focus	Active	Set Capillary	4000 V	Set Dry Heater	200 °C
Scan Begin	50 m/z	Set End Plate Offset	-500 V	Set Dry Gas	4.0 l/min
Scan End	1000 m/z	Set Charging Voltage	2000 V	Set Divert Valve	Source
		Set Corona	0 nA	Set APCI Heater	0 °C

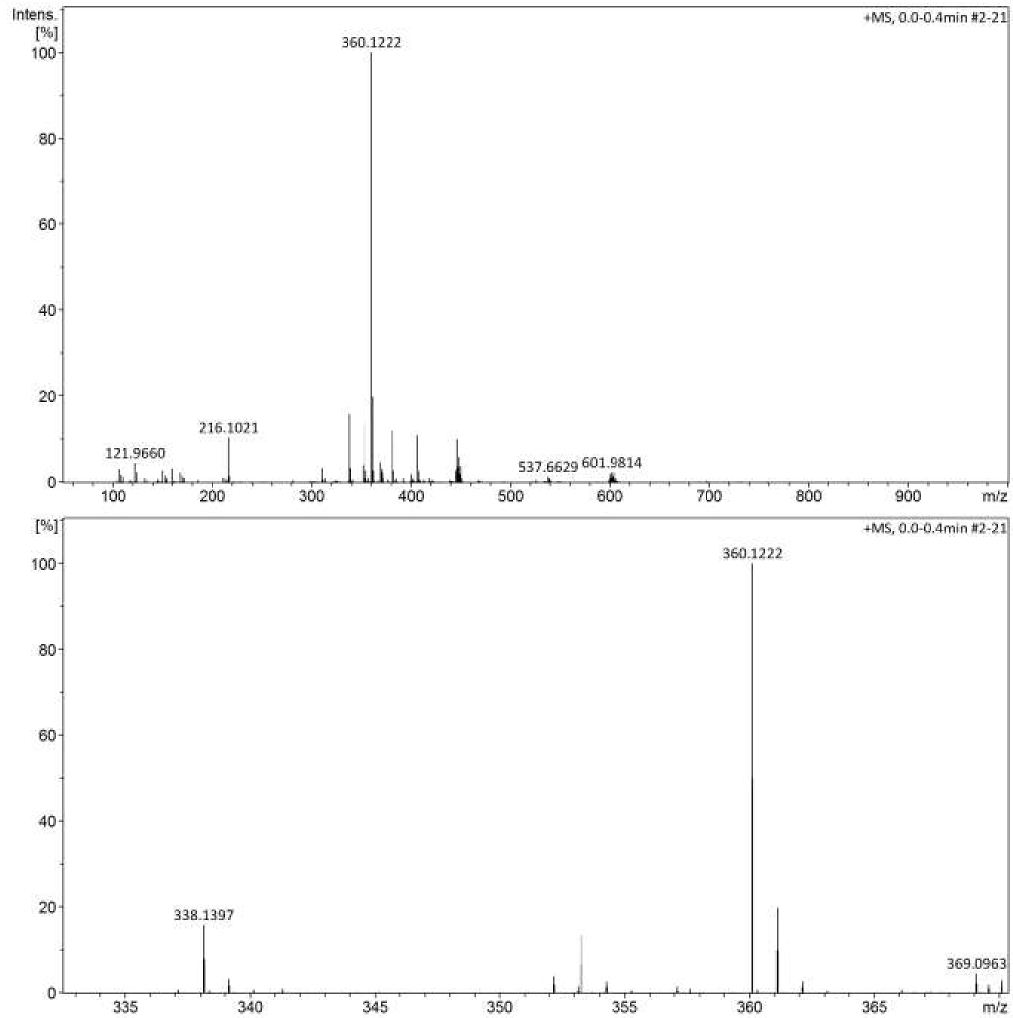

4-[8-(1,3-Dihydro-1,3-dioxo-2*H*-isoindol-2-yl)octyloxy]benzaldehyde (**1b**)

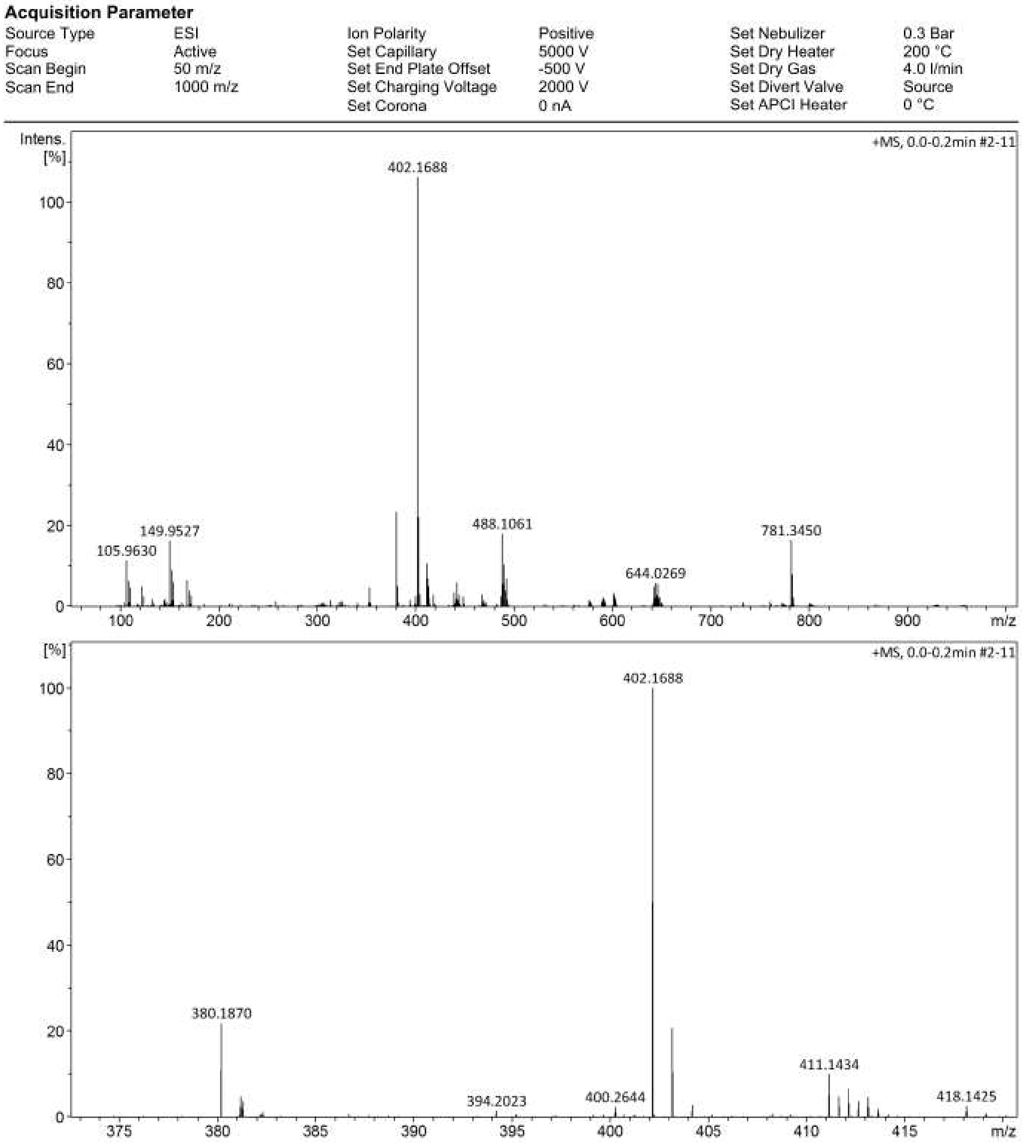

1,3,5,7-Tetramethyl-8-{4-[5-(phthalimidyl)pentyloxy]phenyl}-4,4-difluoro-4-bora-3a,4a-diaza-s-indacene (**2a**)

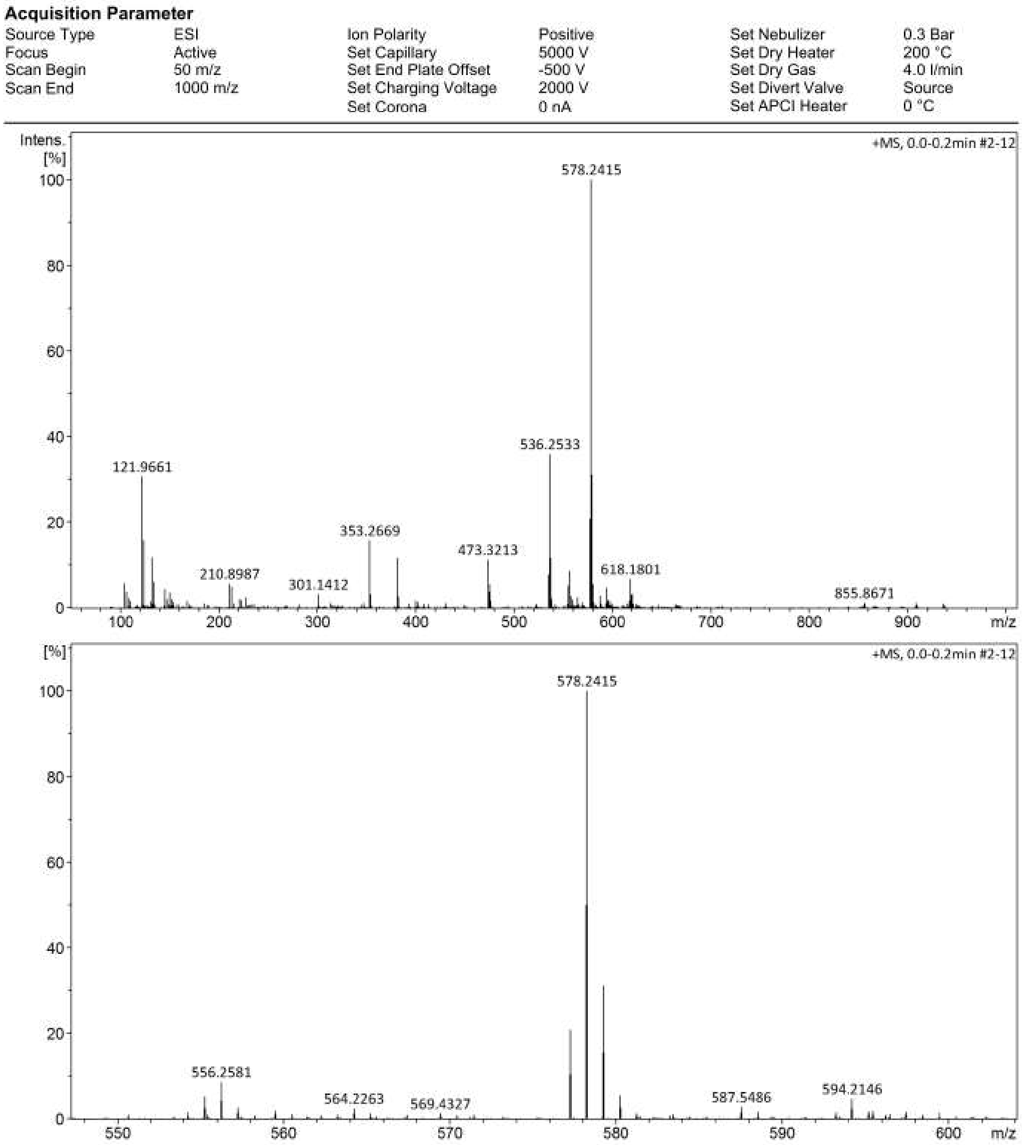

1,3,5,7-Tetramethyl-8-{4-[8-(phthalimidyl)octyloxy]phenyl}-4,4-difluoro-4-bora-3a,4a-diaza-s-indacene (**2b**)

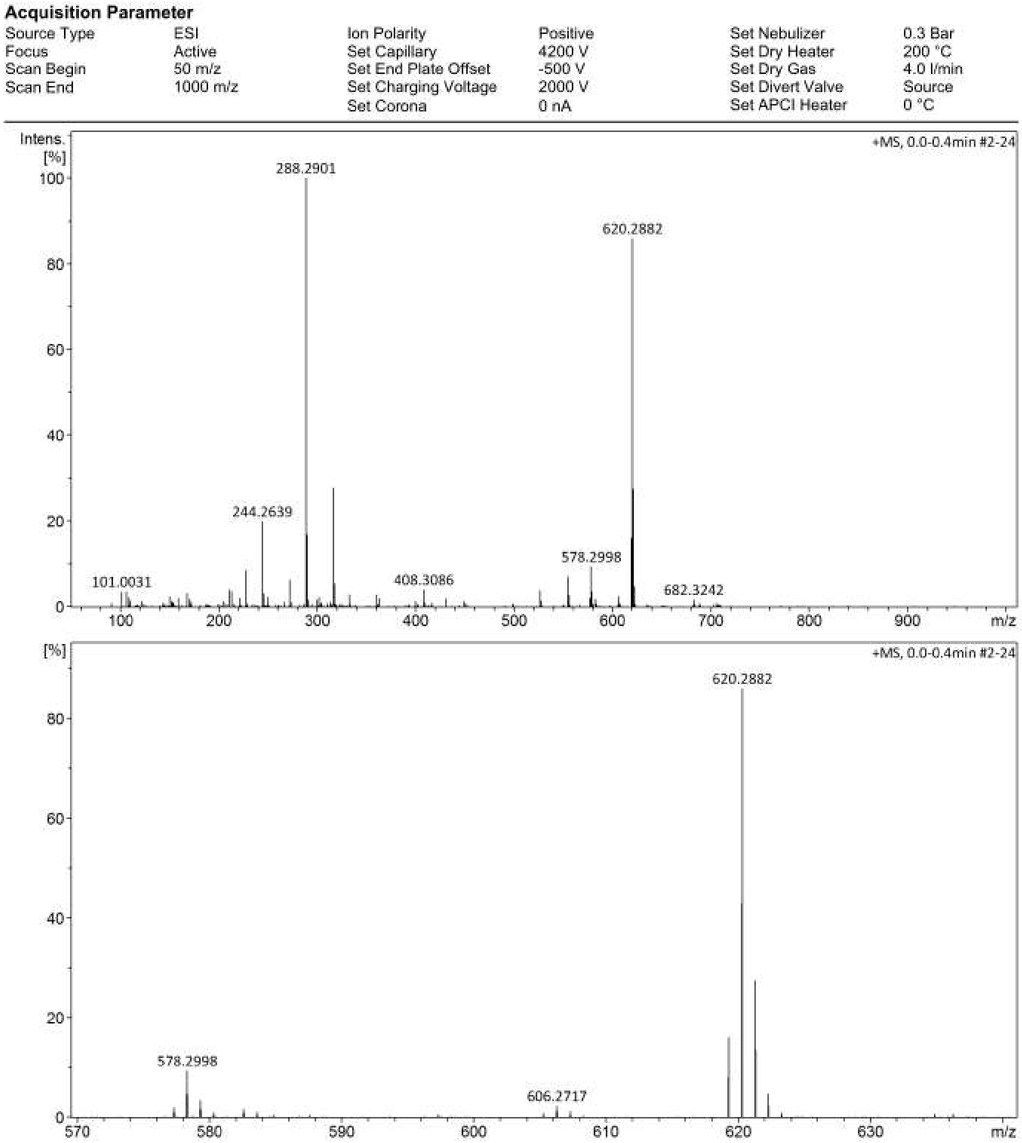

2,6-Diiodo-1,3,5,7-tetramethyl-8-{4-[5-(phthalimidyl)pentyloxy]phenyl}-4,4-difluoro-4-bora-3a,4a-diaza-s-indacene (**3a**)

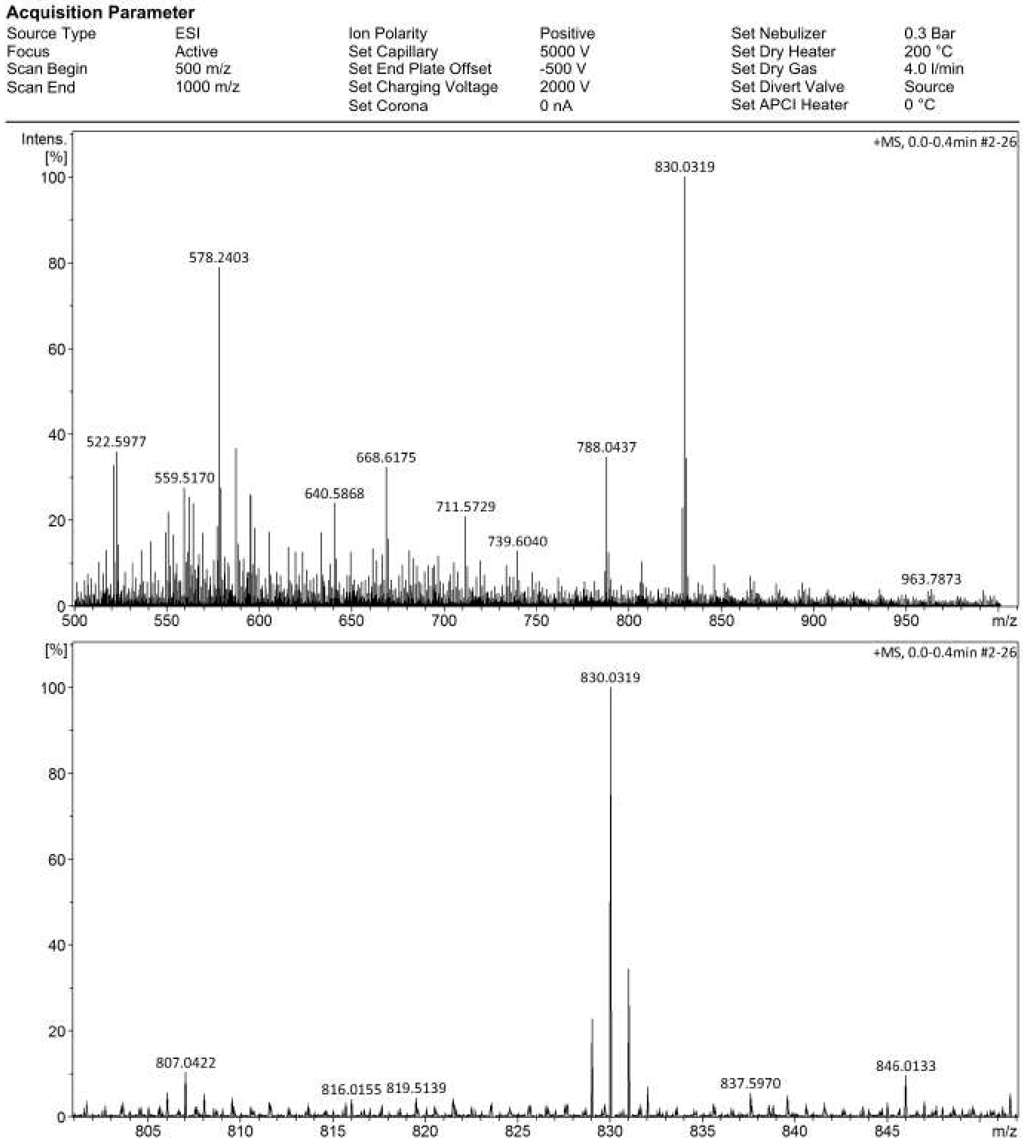

2,6-Diiodo-1,3,5,7-tetramethyl-8-{4-[8-(phthalimidyl)octyloxy]phenyl}-4,4-difluoro-4-bora-3a,4a-diaza-s-indacene (**3b**)

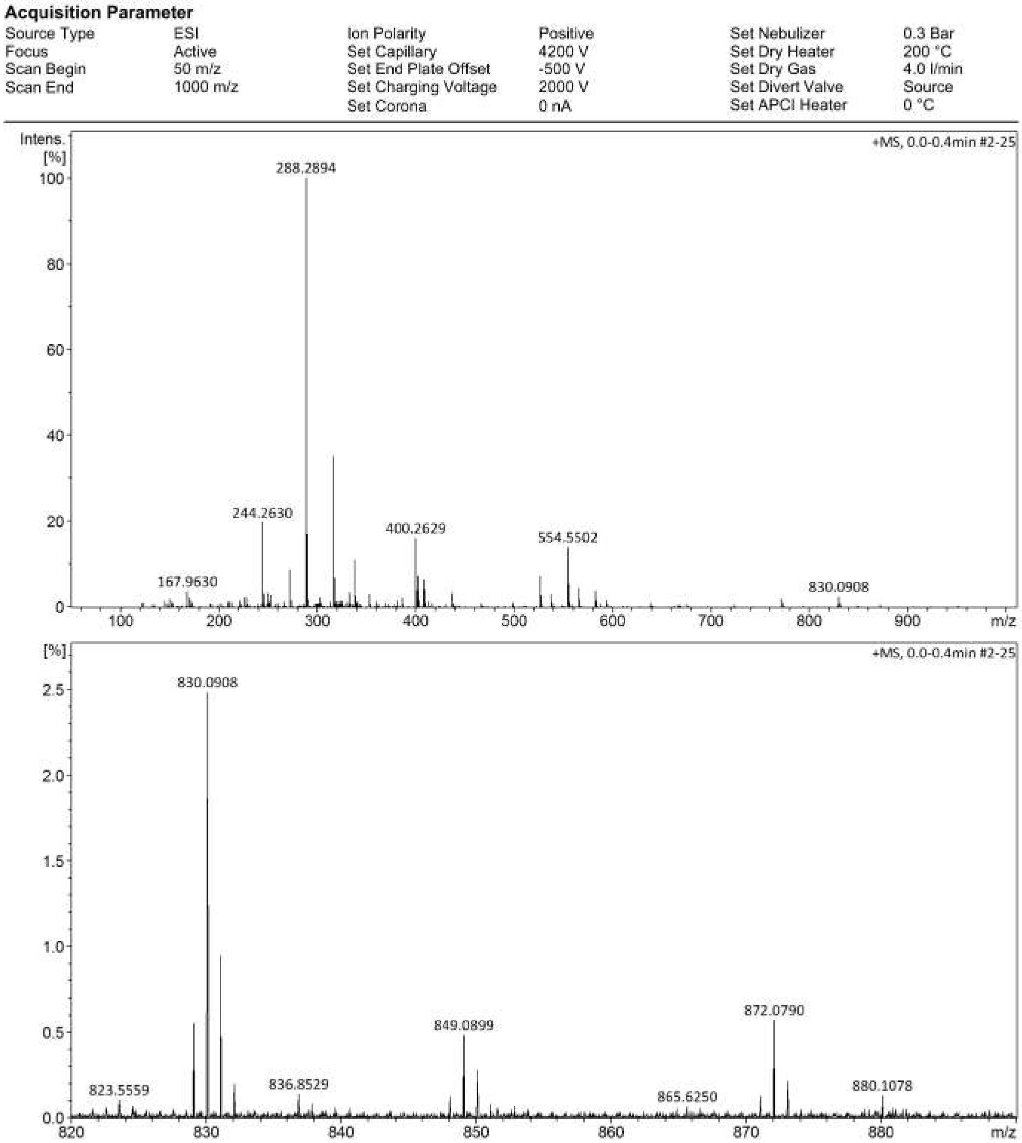

2,6-Dibromo-1,3,5,7-tetramethyl-8-{4-[5-(phthalimidyl)pentyloxy]phenyl}-4,4-difluoro-4-bora-3a,4a-diaza-s-indacene (**4a**)

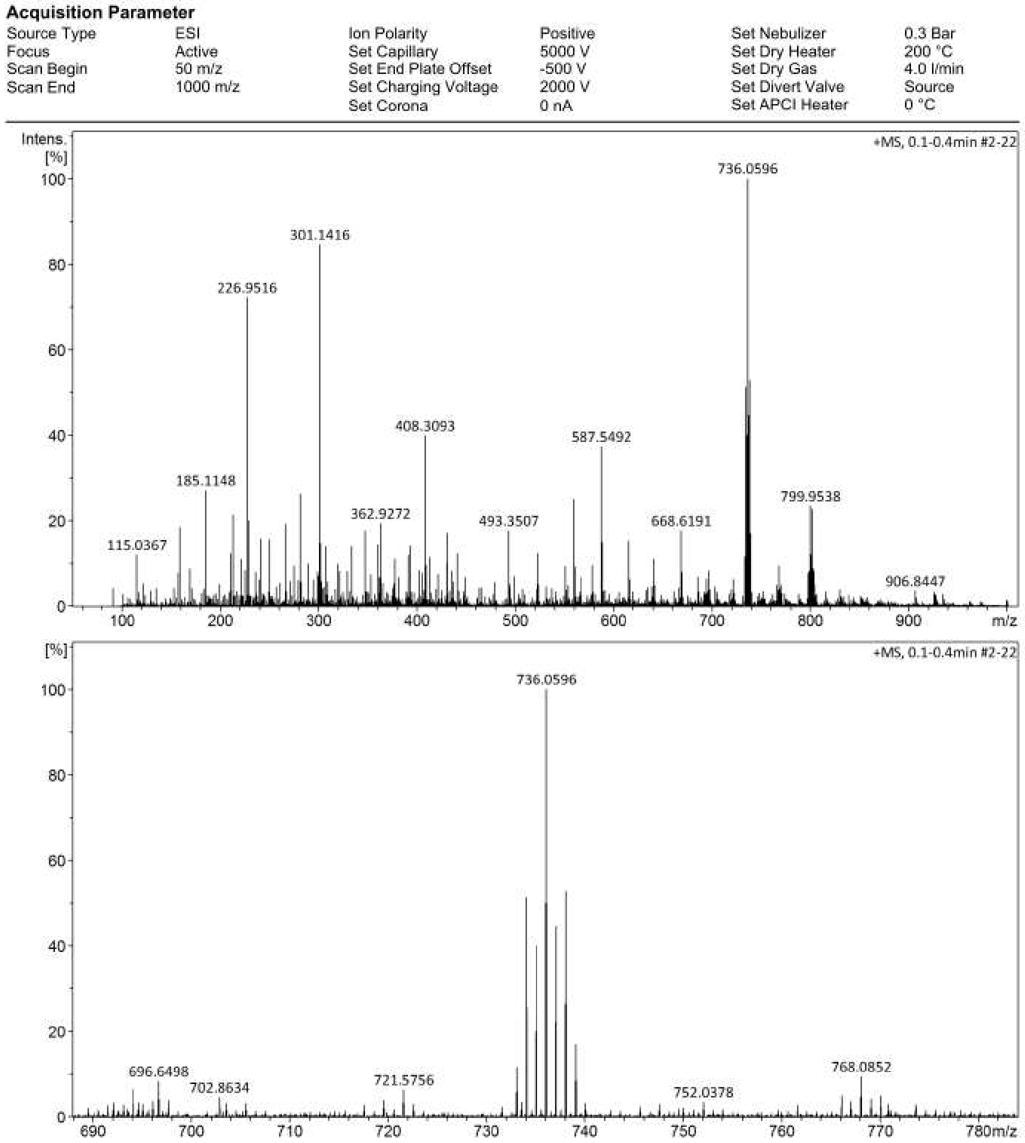

2,6-Dibromo-1,3,5,7-tetramethyl-8-{4-[8-(phthalimidyl)octyloxy]phenyl}-4,4-difluoro-4-bora-3a,4a-diaza-s-indacene (**4b**)

Acquisition Parameter

Source Type	ESI	Ion Polarity	Positive	Set Nebulizer	0.3 Bar
Focus	Active	Set Capillary	4200 V	Set Dry Heater	200 °C
Scan Begin	50 m/z	Set End Plate Offset	-500 V	Set Dry Gas	4.0 l/min
Scan End	1000 m/z	Set Charging Voltage	2000 V	Set Divert Valve	Source
		Set Corona	0 nA	Set APCI Heater	0 °C

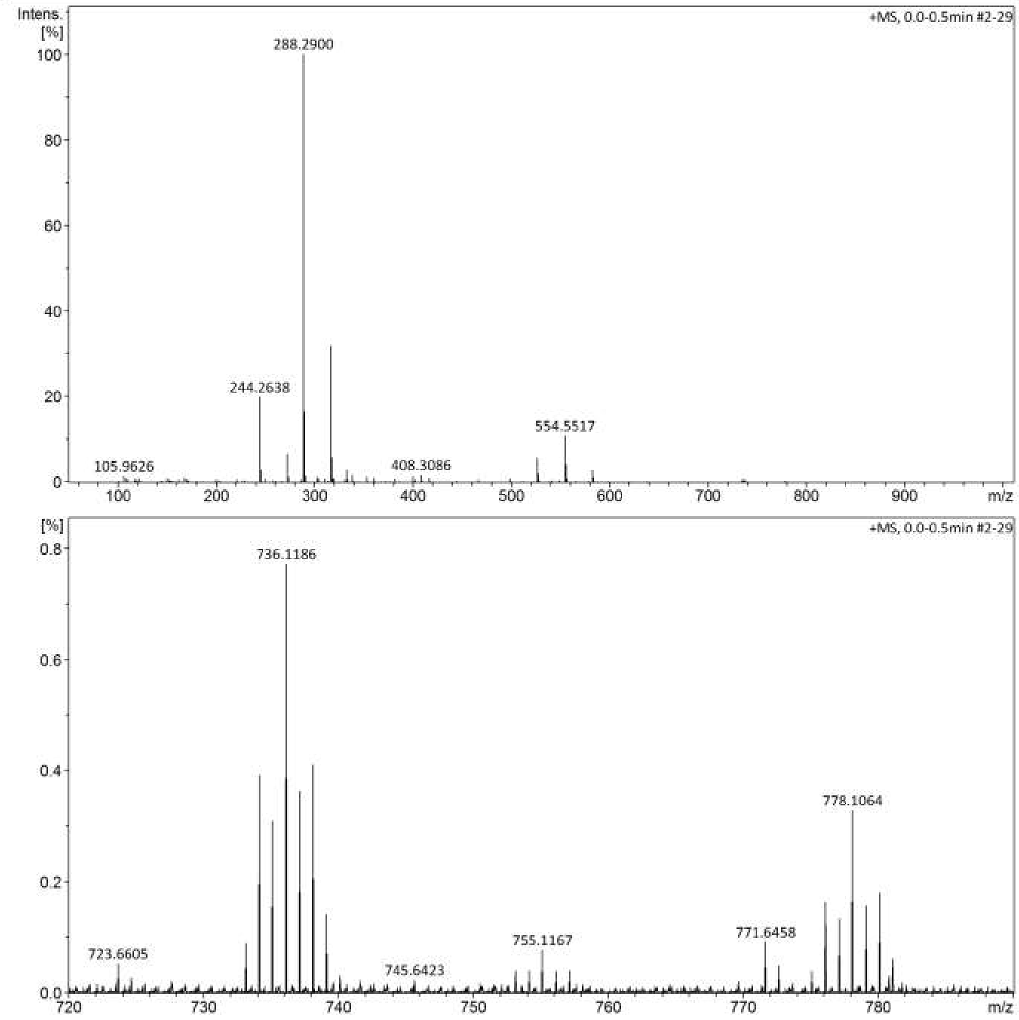

1,3,5,7-Tetramethyl-8-[4-(5-aminopentyloxy)phenyl]-4,4-difluoro-4-bora-3a,4a-diaza-s-indacene (**5a**)

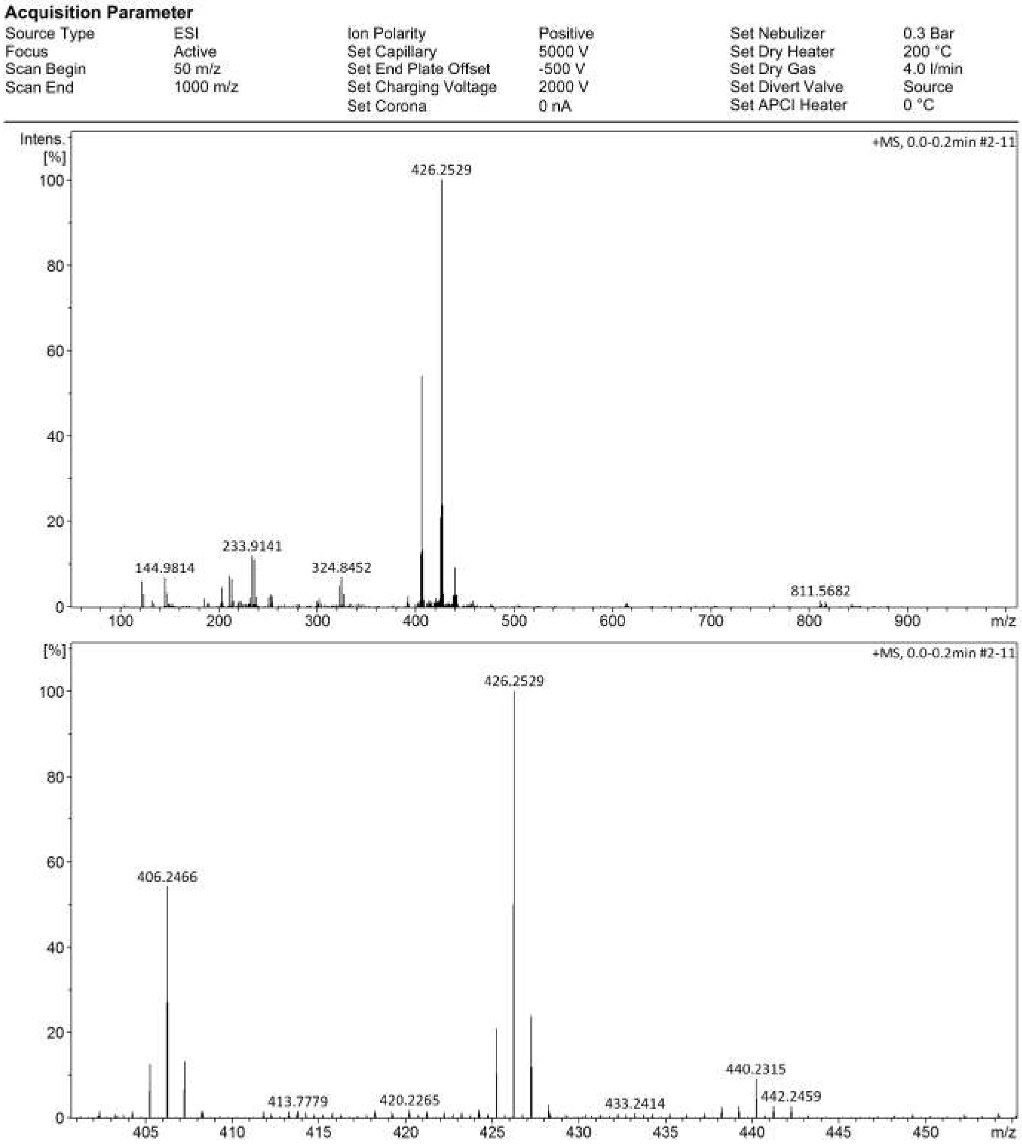

1,3,5,7-Tetramethyl-8-[4-(8-aminooctyloxy)phenyl]-4,4-difluoro-4-bora-3a,4a-diaza-s-indacene (**5b**)

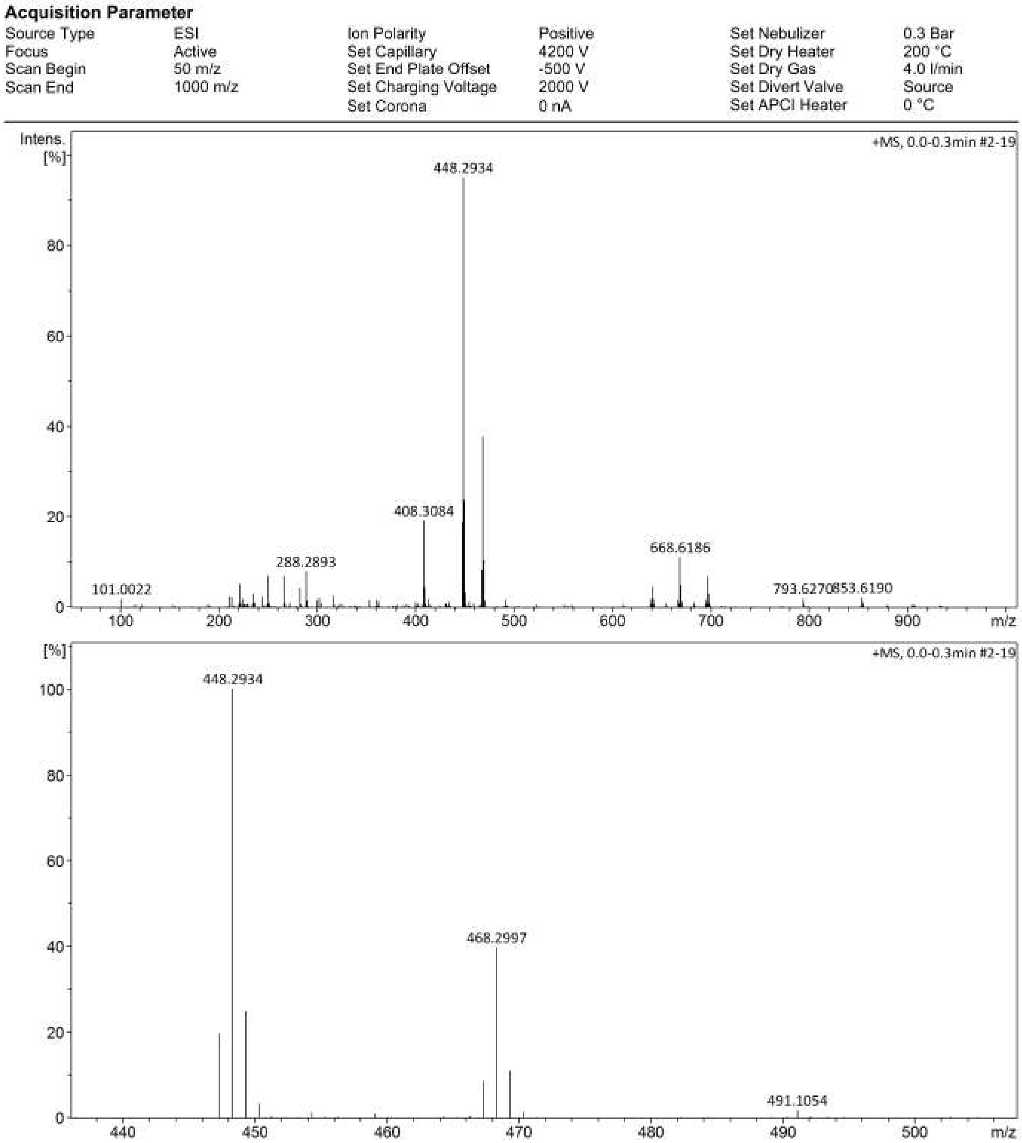

Absorption, emission, excitation spectra

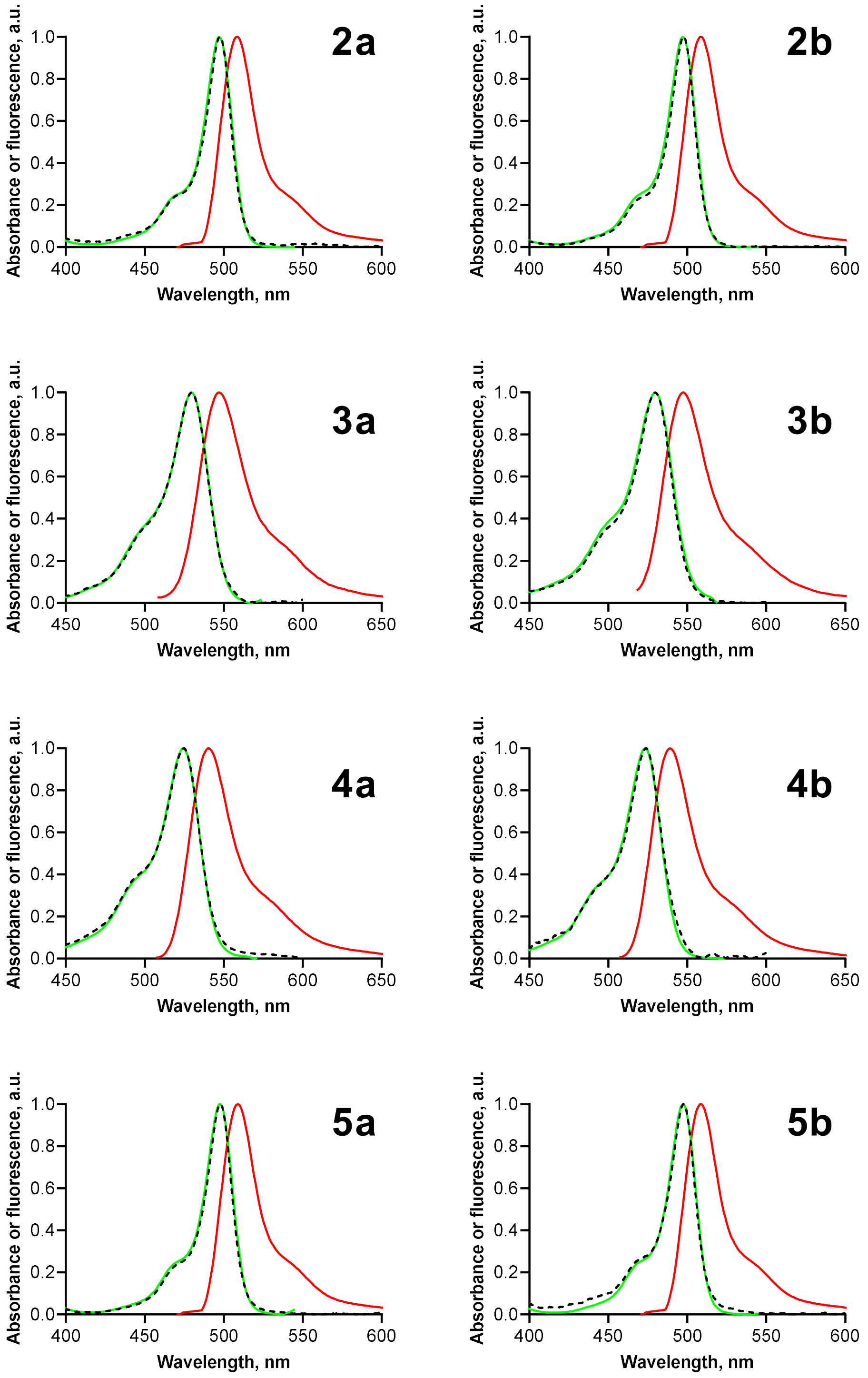

Fig. S11. Normalized absorption (black, dashed), emission (red), and excitation (green) spectra of **2a,b-5a,b** in MeOH (c = 1 μM).

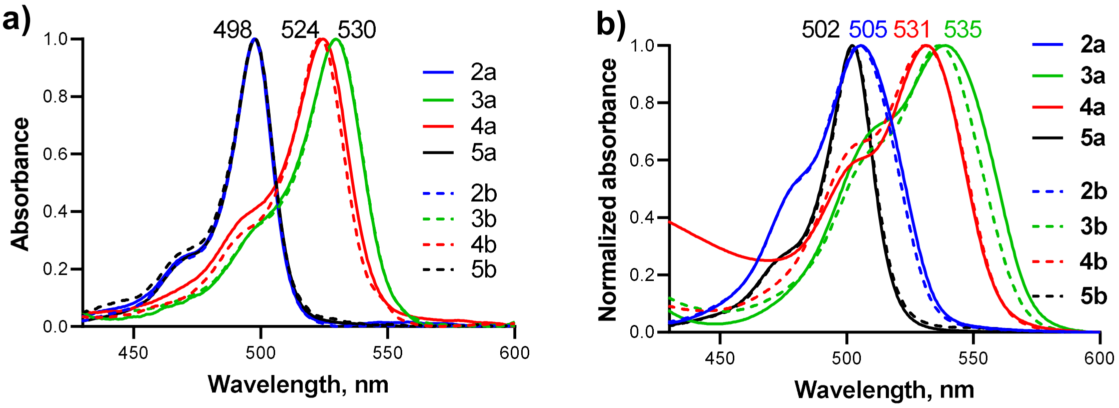

Fig. S12. Normalized absorption spectra of studied compounds in MeOH (a) and serum-containing cell culture medium (b).

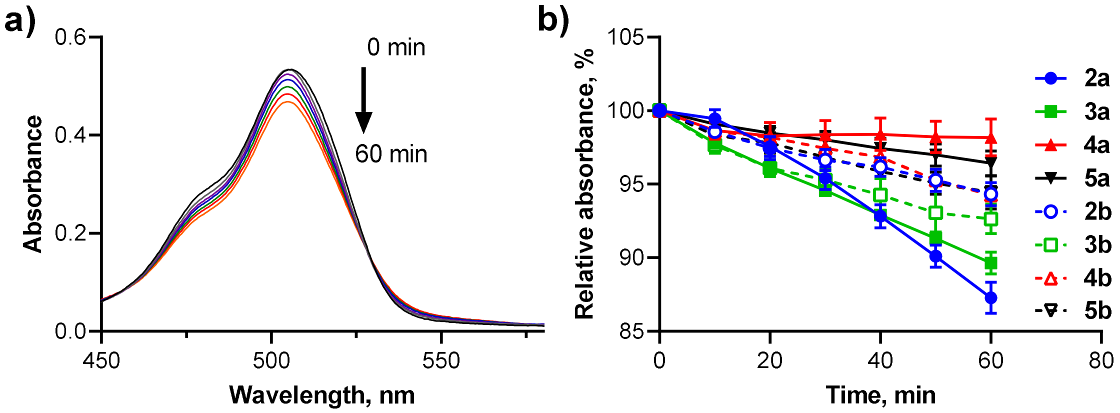

Fig. S13. a) Typical changes of absorption spectra of studied BODIPYs in time (example for **2a**) during photobleaching experiment in serum-containing cell culture medium. b) Time-dependent changes in absorbance (relative to the non-irradiated sample) in absorption maximum of studied compounds (mean and standard deviation of three independent experiments are drawn).

Assessment of biological activity

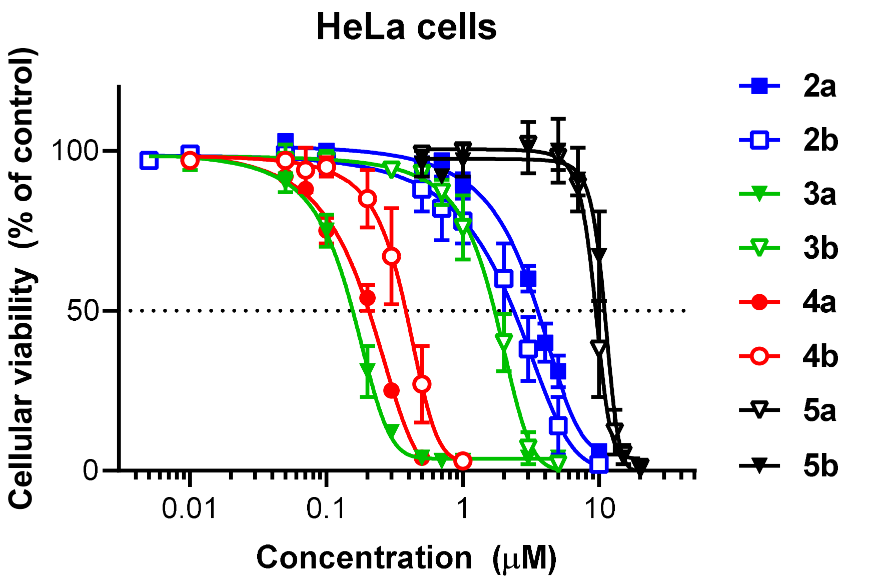

Figure S14. Photodynamic activity of studied compounds on HeLa cell line. At least four independent experiments each in triplicate were performed. Irradiation conditions: λ > 455 nm, 15.3 mW cm^{-2}, 15 min, 13.7 Jcm^{-2}.

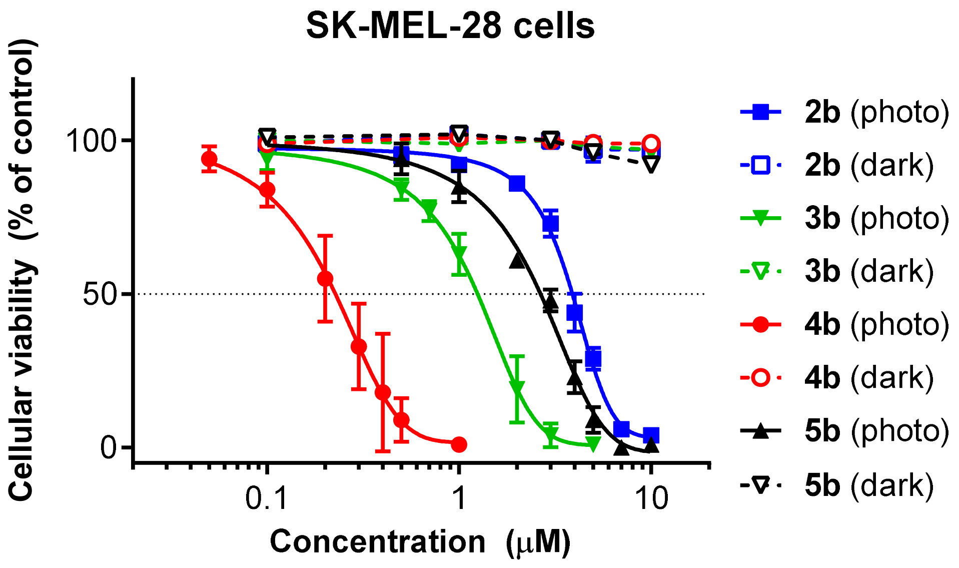

Figure S15. Photodynamic activity (full lines, full symbols) and dark toxicity (dashed lines, empty symbols) of **2b-5b** on SK-MEL-28 cell line. At least four independent experiments each in triplicate were performed. Irradiation conditions: λ > 455 nm, 15.3 mW cm^{-2}, 15 min, 13.7 Jcm^{-2}.

Subcellular localization

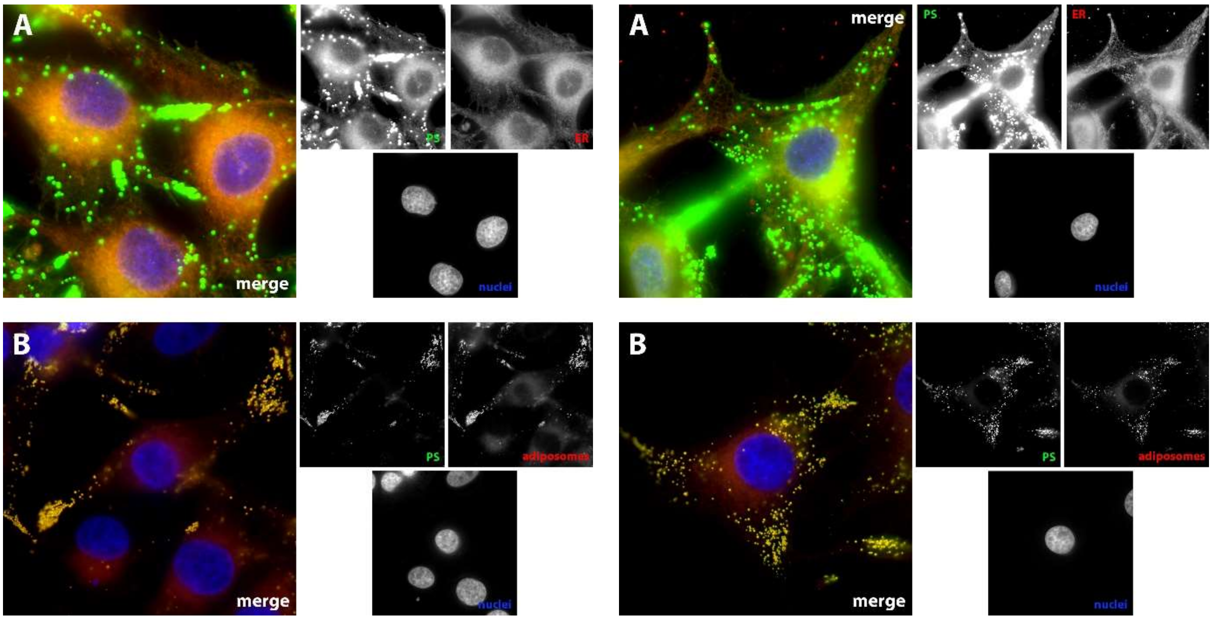

Figure S16. Subcellular localization of compounds **2a** (left) and **2b** (right). A) Intravital staining of nuclei (blue), endoplasmic reticulum (red - mesh) and **2a,b** (green - mesh). Colocalization of **2a,b** with lysosomes represents yellow/orange color in merge image. B) fixed specimen with **2a,b** (green - spots) stained for nuclei (blue) and adiposomes (red - spots). Colocalization of **2a,b** with adiposomes represents yellow/orange color in merge image. PS – photosensitizer (studied BODIPY); ER – endoplasmic reticulum

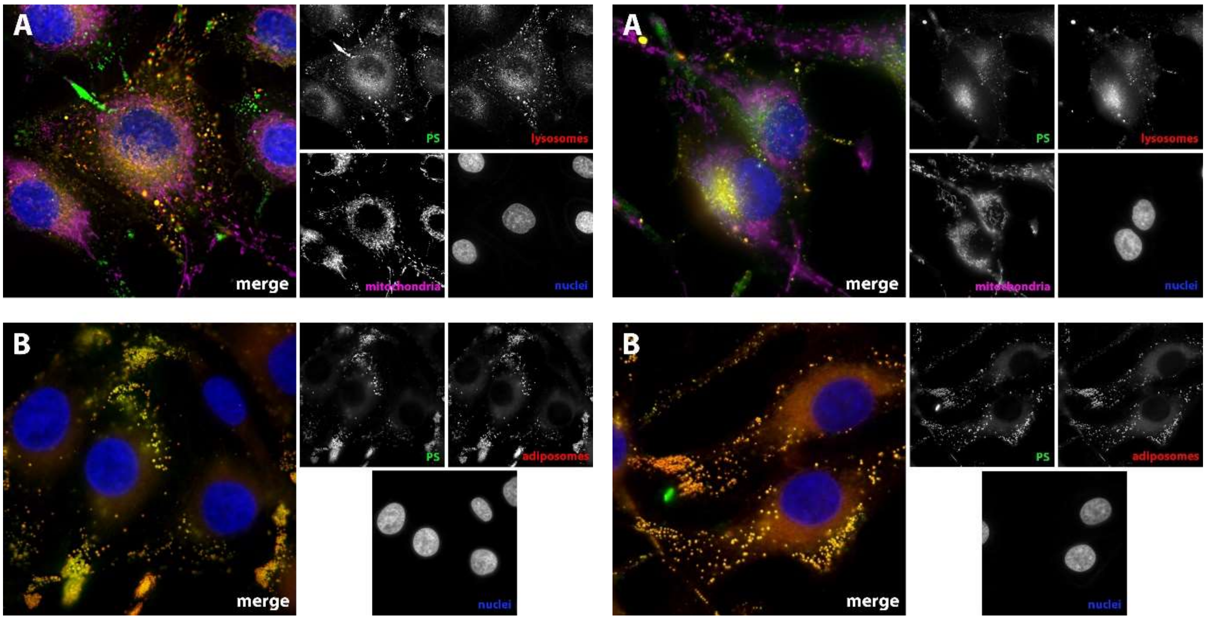

Figure S17 – Subcellular localization of compounds **3a** (left) and **3b** (right). A) Intravital staining of nuclei (blue), mitochondria (pink – rod-like shapes), lysosomes (red – dots) and **3a,b** (green – dots). Colocalization of **3a,b** with lysosomes represents yellow/orange color in merge image. B) fixed specimen with **3a,b** (green – spots) stained for nuclei (blue) and adiposomes (red – spots). Colocalization of **3a,b** with adiposomes represents yellow/orange color in merge image. PS – photosensitizer (studied BODIPY).

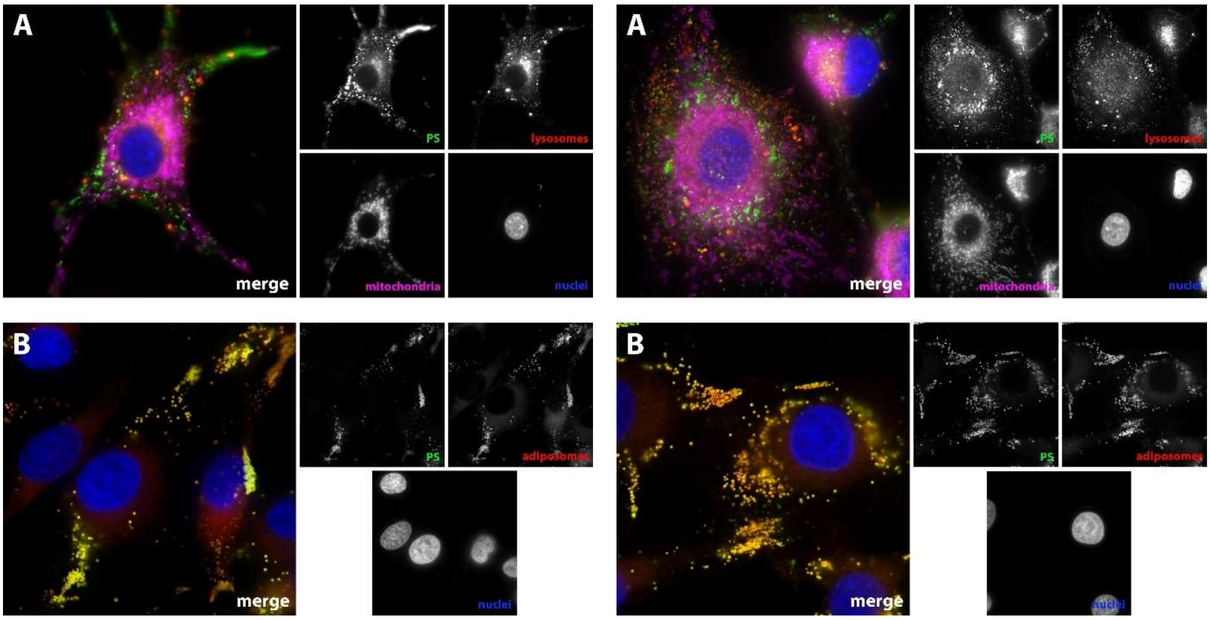

Figure S18 – Subcellular localization of compounds **4a** (left) and **4b** (right). A) Intravital staining of nuclei (blue), mitochondria (pink – rod-like shapes), lysosomes (red – dots) and **4a,b** (green – dots). Colocalization of **4a,b** with lysosomes represents yellow/orange color in merge image. B) fixed specimen with **4a,b** (green – spots) stained for nuclei (blue) and adiposomes (red – spots). Colocalization of **4a,b** with adiposomes represents yellow/orange color in merge image. PS – photosensitizer (studied BODIPY).

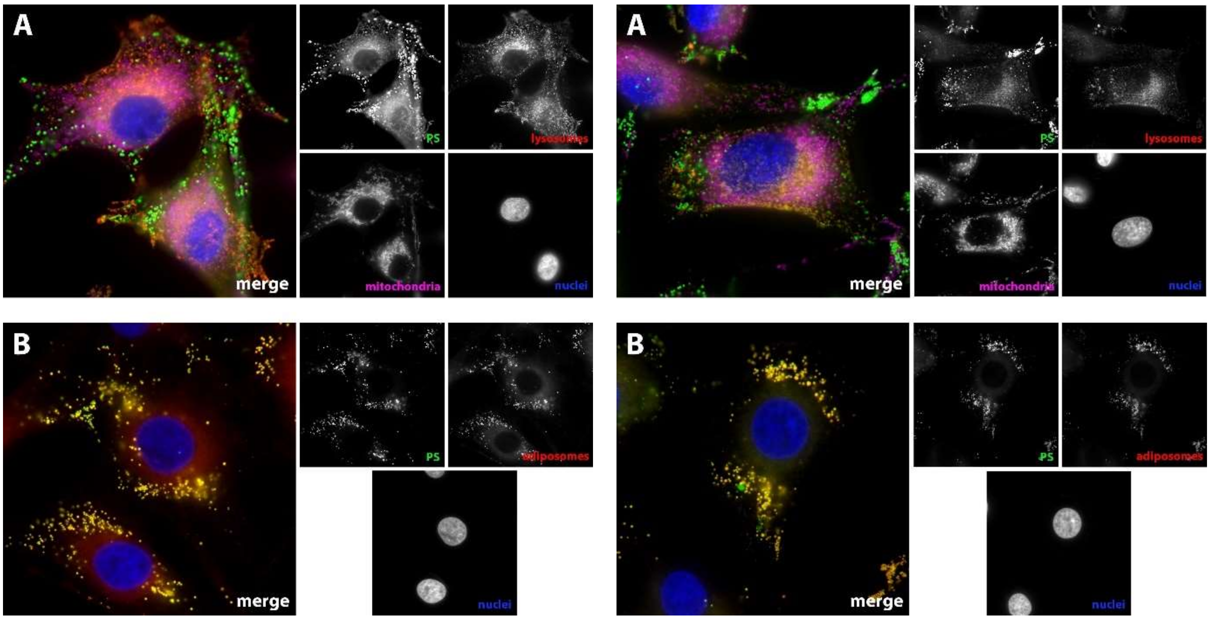

Figure S19 – Subcellular localization of compounds **5a** (left) and **5b** (right). A) Intravital staining of nuclei (blue), mitochondria (pink – rod-like shapes), lysosomes (red – dots) and **5a,b** (green – dots). Colocalization of **5a,b** with lysosomes represents yellow/orange color in merge image. B) fixed specimen with **5a,b** (green – spots) stained for nuclei (blue) and adiposomes (red – spots). Colocalization of **5a,b** with adiposomes represents yellow/orange color in merge image. PS – photosensitizer (studied BODIPY).

Assessment of cell death

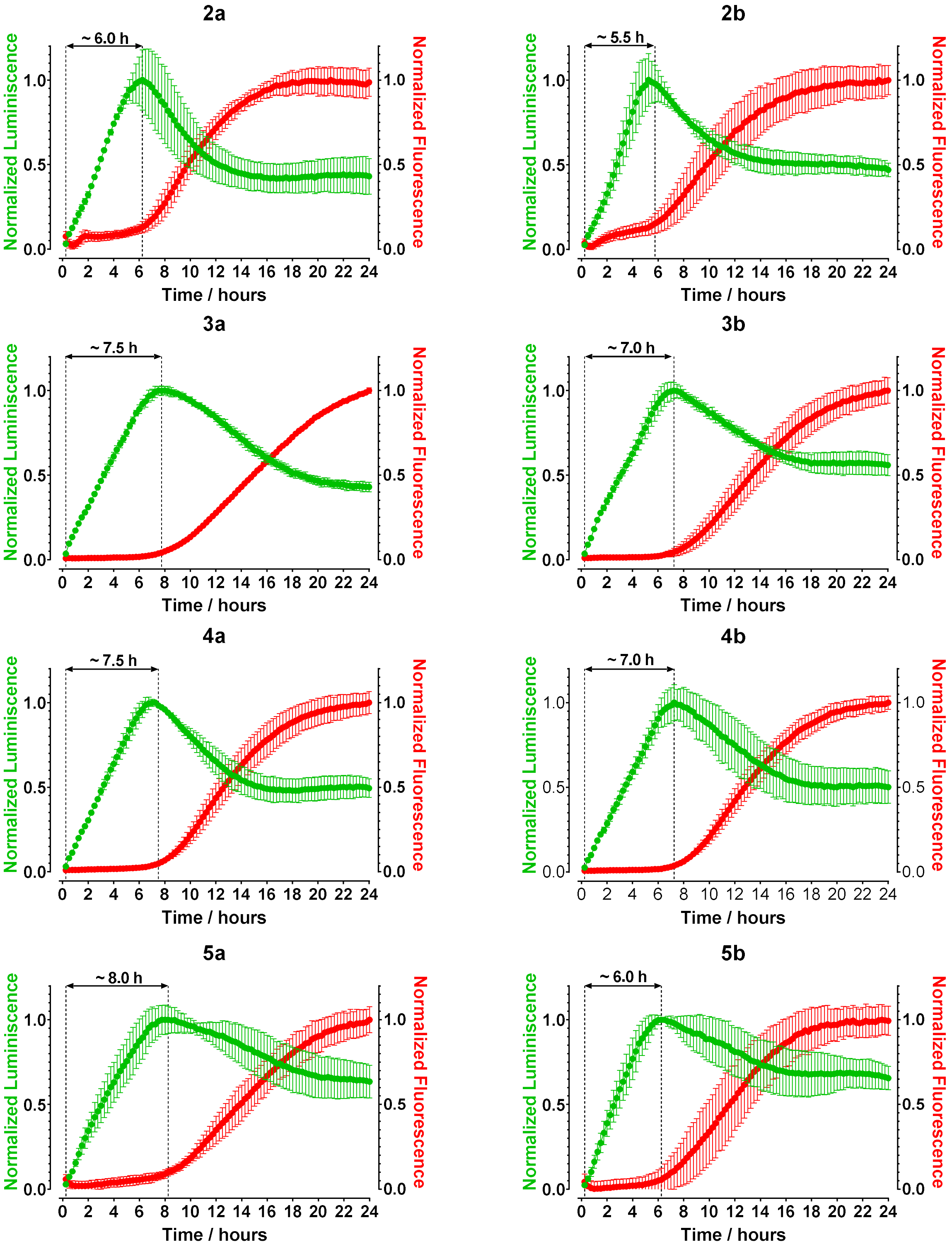

Figure S20. Cell death determination. Annexin V (green) and DNA-binding probe (red) binding profiles for all invetsigated compounds at their EC_{85}.

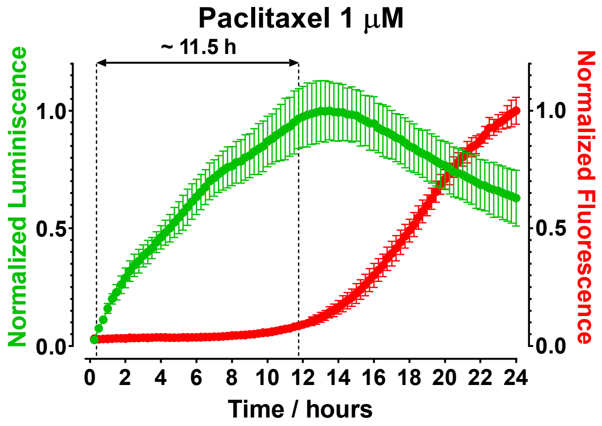

Figure S21. Cell death determination. Annexin V (green) and DNA-binding probe (red) binding profiles for paclitaxel (inducer of intrinsic-pathway apoptosis).
